# Astroglial calcium signaling and homeostasis in tuberous sclerosis complex

**DOI:** 10.1007/s00401-024-02711-3

**Published:** 2024-02-28

**Authors:** Alessia Romagnolo, Giulia Dematteis, Mirte Scheper, Mark J. Luinenburg, Angelika Mühlebner, Wim Van Hecke, Marcello Manfredi, Veronica De Giorgis, Simone Reano, Nicoletta Filigheddu, Valeria Bortolotto, Laura Tapella, Jasper J. Anink, Liesbeth François, Stefanie Dedeurwaerdere, James D. Mills, Armando A. Genazzani, Dmitry Lim, Eleonora Aronica

**Affiliations:** 1 Department of (Neuro) Pathology, Amsterdam UMC, University of Amsterdam, Amsterdam Neuroscience, Amsterdam, The Netherlands; 2 Department of Pharmaceutical Sciences, Università del Piemonte Orientale “Amedeo Avogadro”, Novara, Italy; 3 Department of Pathology, University Medical Center Utrecht, Utrecht, The Netherlands; 4 Center on Autoimmune and Allergic Diseases (CAAD), UPO, Novara, Italy; 5 Department of Translational Medicine, UPO, Novara, Italy; 6 Neurosciences Therapeutic Area, UCB Pharma, Braine-L’Alleud, Belgium; 7 Department of Clinical and Experimental Epilepsy, UCL, London, UK; 8 Chalfont Centre for Epilepsy, Chalfont St Peter, UK; 9 Stichting Epilepsie Instellingen Nederland (SEIN), Heemstede, The Netherlands

**Keywords:** Tuberous sclerosis complex, mTOR, Epilepsy, Calcium signaling, Mitochondria, Astrocytes

## Abstract

Tuberous Sclerosis Complex (TSC) is a multisystem genetic disorder characterized by the development of benign tumors in various organs, including the brain, and is often accompanied by epilepsy, neurodevelopmental comorbidities including intellectual disability and autism. A key hallmark of TSC is the hyperactivation of the mechanistic target of rapamycin (mTOR) signaling pathway, which induces alterations in cortical development and metabolic processes in astrocytes, among other cellular functions. These changes could modulate seizure susceptibility, contributing to the progression of epilepsy and its associated comorbidities. Epilepsy is characterized by dysregulation of calcium (Ca^2+^) channels and intracellular Ca^2+^ dynamics. These factors contribute to hyperexcitability, disrupted synaptogenesis, and altered synchronization of neuronal networks, all of which contribute to seizure activity. This study investigates the intricate interplay between altered Ca^2+^ dynamics, mTOR pathway dysregulation, and cellular metabolism in astrocytes. The transcriptional profile of TSC patients revealed significant alterations in pathways associated with cellular respiration, ER and mitochondria, and Ca^2+^ regulation. TSC astrocytes exhibited lack of responsiveness to various stimuli, compromised oxygen consumption rate and reserve respiratory capacity underscoring their reduced capacity to react to environmental changes or cellular stress. Furthermore, our study revealed significant reduction of store operated calcium entry (SOCE) along with strong decrease of basal mitochondrial Ca^2+^ concentration and Ca^2+^ influx in TSC astrocytes. In addition, we observed alteration in mitochondrial membrane potential, characterized by increased depolarization in TSC astrocytes. Lastly, we provide initial evidence of structural abnormalities in mitochondria within TSC patient-derived astrocytes, suggesting a potential link between disrupted Ca^2+^ signaling and mitochondrial dysfunction. Our findings underscore the complexity of the relationship between Ca^2+^ signaling, mitochondria dynamics, apoptosis, and mTOR hyperactivation. Further exploration is required to shed light on the pathophysiology of TSC and on TSC associated neuropsychiatric disorders offering further potential avenues for therapeutic development.

**Supplementary Information:**

The online version contains supplementary material available at 10.1007/s00401-024-02711-3.

## Introduction

Tuberous Sclerosis Complex (TSC) is a multisystem genetic disorder, characterized by the development of benign tumors throughout the body, including the brain, eyes, skin, kidneys, heart, and lungs [73]. Epilepsy represents the most prevalent neurologic manifestation in TSC patients, affecting approximately 80–90% of individuals [27, 73, 95]. Neurodevelopmental comorbidities including intellectual disability (ID) and autism spectrum disorder (ASD) are also common in TSC [27, 95]. Cortical tubers are unique focal developmental lesions integrated into the intricate pathologic cellular network of TSC brain that drives both epileptogenesis and co-occurring neurodevelopmental disorders [7, 27]. TSC is caused by loss of function mutations in the *TSC1* and *TSC2* genes, which lead to overactivation of the mechanistic target of rapamycin (mTOR) signaling pathway [27]. Dysregulation of the mTOR pathway in the brain leads to altered development of human cortical structure and organization, protein synthesis, autophagy, and metabolism [26, 71, 100]. Our understanding of the intricate cellular and molecular consequences of mTOR complex 1 (mTORC1) overactivation, which contribute to the epileptogenic process in individuals with TSC is advancing rapidly [7]. A recent comprehensive transcriptomic study has identified several key hallmarks of TSC, which encompass mechanisms related to neurotransmission and synaptic plasticity, immune response and neuroinflammation, brain extracellular matrix, energy metabolism, neuronal support, and myelination [2]. These dysregulated pathways involve different cell types however the direct roles of astrocytes in modulating seizure susceptibility and the progression of epilepsy in TSC has been reported in multiple studies [12, 102, 106, 116].

Within the brain and specifically in the astrocytes, calcium (Ca^2+^) plays a pivotal role including regulation of signal transduction, neurotransmitter release and uptake, gene expression, cell metabolism and synaptic pruning during development [89]. Cytosolic Ca^2+^ levels ([Ca^2+^]_c_) are finely regulated and kept at ~ 50–100 nM resting [Ca^2+^], while in the endoplasmic reticulum (ER) lumen ([Ca^2+^]_ER_) reaches 0.1–1 mM. In the mitochondrial matrix mitochondrial calcium ([Ca^2+^]_m_) is kept at sub-micromolar values during stimulation, Ca^2+^ transients can reach ≤ 100 μM. The maintenance of [Ca^2+^]_ER_ is crucial for different physiologic processes, such as protein folding, and it is tightly regulated by the SOCE [80]. Reduction of luminal [Ca^2+^]_ER_ is sensed by the stromal interaction molecule (STIM) proteins, STIM1 and STIM2 [59, 87] which oligomerize and migrate to the ER-plasma membrane contact sites. Upon activation, STIM proteins interact with Orai Ca^2+^-permeable channels, Orai1, Orai2 and Orai3 [37, 45], to allow Ca^2+^ influx into the cytosol from the extracellular space and replenish Ca^2+^ stores in the ER via the sarcoplasmic/ER Ca^2+^ ATPases (SERCAs). In addition, Ca^2+^ influx in the cytosol is also mediated by transient receptor potential channels (TRPCs). Upon specific stimuli, that activates metabotropic or ionotropic responses, Ca^2+^ is released from the ER via ryanodine receptors (RyRs) and inositol 1,4,5-triphosphate receptors (Ins(1,4,5)P_3_Rs) [11, 23]. ER Ca^2+^ release induces mitochondrial Ca^2+^ uptake too, via IP_3_R-VDAC1 complex. This complex allows the formation of high Ca^2+^ hotspots in the correspondence of Mitochondrial Ca^2+^ Uniporter (MCU), the low affinity channel that allows the passage of Ca^2+^ across the inner mitochondrial membrane (IMM). Furthermore, an alternative mechanism for mitochondrial Ca^2+^ uptake occurs when Ca^2+^ ions might be efficiently transported directly to mitochondria situated in proximity to the cell membrane during SOCE. This alternative mechanism facilitates the uptake of excess Ca^2+^ and prevents cytosolic Ca^2+^ overload coordinating the cellular bioenergetics and maintaining the mitochondrial retrograde signaling pathways [89].

Several studies have demonstrated that dysregulation of Ca^2+^ signaling in astrocytes is associated with various neurodegenerative diseases [i.e., Alzheimer’s disease (AD), Parkinson’s disease (PD), and Huntington’s disease (HD)], epilepsy, mood disorders [i.e., ASD and ID], and neurodevelopmental disorders [11, 50, 56, 69, 93, 119]. This dysregulation contributes to altered neuronal excitability, synaptic dysfunction, and neuronal degeneration [28, 91]. In the context of epilepsy, it is known that dysregulation of Ca^2+^ channels and intracellular Ca^2+^ dynamics with [Ca^2+^]_m_ overload can lead to hyperexcitability, disrupt synaptogenesis and altered synchronization of neuronal networks, which contribute to seizure activity [8, 42, 64, 98]. However, the intricate relationship between Ca^2+^ signaling, mTOR pathway dysregulation, and epileptogenesis requires further investigation.

The mTOR signaling pathway is known to be sensitive to intracellular [Ca^2+^] and mobilization. Although the specific molecular mechanisms underlying the regulation of mTOR signaling by Ca^2+^ are not fully understood, recent studies have shed light on possible regulatory mechanisms [4, 25, 43, 44, 55]. For example, recent findings revealed that lysosomal Ca^2+^ and calmodulin (CaM) play essential regulatory roles in the mTORC1 signaling pathway and stimulate its kinase activity inducing the transcription of the pro-inflammatory secretome (SASP) [55]. In addition, the AKT/mTOR signaling pathway can in turn regulate ER stress via KCa3.1 during reactive astrogliosis [111]. Furthermore, Ca^2+^-dependent activation of the kinase CaMKKβ can lead to mTOR pathway activation and regulate downstream cellular processes such as autophagy [60].

While extensive literature is available on the impact of mTOR hyperactivation on intracellular Ca^2+^ dynamics and mitochondria biogenesis in neurons, there is a notable gap in studies focusing on other cell types within the context of TSC. However, the pronounced gliosis observed in TSC tubers suggests the importance of exploring non-neuron centric molecular mechanisms [116]. We hypothesize that mTOR hyperactivation in astrocytes could lead to alterations in intracellular Ca^2+^ levels through the activity of CaM thereby further activating mTOR and increasing gliotransmitter release. This could establish a positive feedback loop, promoting neuronal hyperexcitability. Moreover, the elevated levels of reactive oxygen species (ROS) in TSC astrocytes imply dysregulation of ER and mitochondrial functionality. In our disease model, this dysregulation may lead to decreased Ca^2+^ concentration in the ER, subsequently increasing Ca^2+^ release into the mitochondria, thereby inducing ROS generation in the mitochondrial respiratory chain and establishing a positive feedback loop. In addition, mTOR’s regulation of mitochondria biogenesis may further elevate ROS production, potentially affecting cytosolic Ca^2+^ levels and Ca^2+^ storage in organelles.

Taken together, the interplay between the mTOR pathway and Ca^2+^ signaling in astrocytes suggests a complex regulatory mechanism, wherein Ca^2+^ signals could influence mTOR signaling, and conversely, mTOR signaling might reciprocally affect Ca^2+^ signals. In this study, we explore the intricate relationship between the alterations in Ca^2+^ dynamics, cell metabolism, and mTOR pathway dysregulation. Understanding this cross-talk is essential for unraveling the role of Ca^2+^ in astrocytes, providing valuable insights in the pathophysiology of mTOR-related epileptogenesis of TSC and may pave the way for potential targeted therapeutic interventions.

## Materials and methods

### Subjects

Surgical and post-mortem tissues included in this study were obtained from the archives of the Departments of Neuropathology of the Amsterdam UMC, (Amsterdam, the Netherlands) and the UMC Utrecht (Utrecht, the Netherlands). Cortical brain samples were obtained from patients undergoing surgery for intractable epilepsy and diagnosed with TSC cortical tubers (bulk RNA sequencing: *n* = 21, single-cell RNA sequencing: *n* = 5). In addition, age- and tissue-matched autopsy control samples were collected (bulk RNA sequencing: *n* = 15, single-cell RNA sequencing: *n* = 3) from individuals without a history of seizures or other neurologic disease. Clinical details were reported in Online Resource 1.

All cases were reviewed independently by two neuropathologists (AE and AM). Patients who underwent implantation of strip and/or grid electrodes for chronic subdural invasive monitoring before resection and patients who underwent previous respective epilepsy surgery were excluded from this study. All patients with cortical tubers fulfilled the diagnostic criteria for TSC cortical tubers (including genetic analysis for the detection of germline mutations) [73].

### RNA isolation

RNA isolation from human frozen brain tissue and cell culture material was done using the miRNeasy Mini kit (Qiagen Benelux, Venlo, the Netherlands) according to the manufacturer’s instructions. The concentration and purity of RNA were determined using a Nanodrop 2000 spectrophotometer (ThermoFisher Scientific, Wilmington, DE, USA). The RNA was stored at − 80 °C until use.

### Bulk RNA sequencing

All library preparation and sequencing were performed at GenomeScan (Leiden, the Netherlands). The NEBNext Ultra II Directional RNA Library Prep Kit for Illumina (New England Biolabs, Ipswich, MA, USA) was used for sample processing. Sample preparation was performed according to the protocol “NEBNext Ultra II Directional RNA Library prep Kit for Illumina” (NEB#E7760S/L). Briefly, mRNA was isolated from total RNA using oligo-dT magnetic beads. After fragmentation of mRNA, cDNA synthesis was performed. Next, sequencing adapters were ligated to the cDNA fragments followed by PCR amplification. Clustering and DNA-sequencing was performed using the NovaSeq6000 (Illumina, Foster City, CA, USA) in accordance with manufacturers’ guidelines. All samples underwent paired-end sequencing of 150 nucleotides in length, the mean read depth per a sample was 47 million reads, of which ~ 84% mapped uniquely and concordantly to the human reference genome GRCh38.

Differential expression analysis compared 21 TSC patients and 14 age-matched control cortices. The gene counts were than normalized using the weighted trimmed mean of M-values (TMM) method using the R package edgeR [22, 86, 86]. The normalized counts were log2 transformed using the voom function from the R package limma. The impact of the diagnosed pathology and the surgical area of resection on gene expression was assessed and visualized by Principal Component Analysis (PCA) and if required a batch correction was performed. Subsequently, differential expression analysis was performed using limma. Briefly, a linear model was fit for each gene and moderated t-statistic was calculated after applying an empirical Bayes smoothing the standard errors [84, 84]. Those genes with a Benjamini-Hochberg (BH) adjusted *p* value < 0.05 (p.adj < 0.05) were considered differentially expressed. Pathway enrichment analysis were performed using the R package clusterProfiler [109, 110, 109] providing a statistical analysis and visualization of GO functional profiles of genes.

### Single-cell RNA sequencing

Single-cell RNA sequencing was performed at Single Cell Discoveries (https://www.scdiscoveries.com/) according to the 10 × genomics Chromium Single Cell Gene Expression Flex protocol. Prior to loading the samples, the frontal cortex tissue was cut into slices, which were fixed, and cells were extracted. Cells were counted to ensure quality control. For each sample, 8000 cells were loaded, and the resulting sequencing libraries were prepared following a standard 10 × Genomics protocol.

Sample reads were aligned to the human genome GRCh38 using Cell Ranger[114]. Filtering of empty barcodes was done in Cell Ranger. The data from all samples were loaded in R (version 4.3.1) and processed using the Seurat package (version 5.0.1) [47]. More specifically, cells with at least 1000 UMIs per cell and less than 5% mitochondrial gene content were retained for analysis. The data of all 10 × libraries was merged and processed together. The merged dataset was normalized for sequencing depth per cell and log transformed. After filtering, datasets were integrated using reciprocal principal component analysis (RPCA). The integrated data was subsequently scaled, and dimensionality reductions were performed. Clustering was computed using the FindNeighbors function (dims = 1:20), and FindClusters at a resolution of 0.5. To identify the cell types in separate clusters, marker genes for each cell type were used (Online Resource 2). To perform differential expression analysis between control and TSC samples, we performed pseudo-bulk analysis. This approach involves aggregating cells within each biologic sample to create ‘pseudobulks’. Differential expression analysis was performed using the R package DESeq2 [61]. To control the false discovery rate, we applied the Benjamini–Hochberg correction, considering gene expression changes with an adjusted *p* value < 0.05 as statistically significant.

### Control and TSC primary cultures

Astrocytes were isolated using a papain dissection method (Worthington Biochemical, Lakewood, NJ, USA) from surgically resected TSC patients between the age of 3 and 26 years, while for control material, astrocytes were isolated from human control brain tissue derived from abortions occurred between gestational week 12 and 16. All tissue was collected with written consent and according to the declaration of Helsinki as well as the Amsterdam research code of the medical ethics committee. In details, control and TSC brain tissues were collected in DMEM/F12 (Gibco, Thermo Fisher Scientific, Walthman, MA, USA) + 10% Fetal Calf Serum (10% FCS) + 100 ug/ml penicillin and 100 ug/ml streptomycin (1% P/S) media. Before the dissociation, the tissue was washed and collected into 13 mm plates containing tissue cutting buffer (dPBS, 10 µM Y-27632). It was mechanically homogenized and it was added to a papain solution (1 × HBSS, 0.46% glucose, 25 mM HEPES pH7.5, 1.7 mM L-cysteine, 10 µM Y-27632) with 7.5 units/mL papain for control and 20 units/mL for TSC tissue. The tissue was incubated for 40–60 min at 34 °C with intermittent mixing. The protease was deactivated with inhibitor solution (DMEM/F12, 1% FCS, 0.0005% DNase) and gently broken up by repeated pipetting through a serological pipet, moving the supernatant with single cells into a fresh tube. Cells were spun down and suspended in complete astrocyte media (DMEM/F10 1:1, 10% FCS, 1 mM glutamine, 100 µg/ml penicillin, 100 µg/ml streptomycin) and filtered with a cell strainer (70 µM). Flasks were incubated at 37 °C under a humidified 5% CO_2_-containing atmosphere. All experiments were performed with cells passage 3–7.

### Proteomics

Cells were collected, washed, lysed with RIPA buffer and sonicated. Proteins were then precipitated with cold acetone and resuspended. Proteins were then reduced in 25 µL of 100 mM NH_4_HCO_3_ with 2.5 μL of 200 mM DTT (Merck) at 60 °C for 45 min and next alkylated with 10 μL 200 mM iodoacetamide (Merck) for 1 h at RT in dark conditions. Iodoacetamide excess was removed by the addition of 200 mM DTT. The digests were dried by Speed Vacuum and then desalted [63].

Digested peptides were analyzed with a UHPLC Vanquish system (Thermo Scientific, Rodano, Italy) coupled with an Orbitrap Q-Exactive Plus (Thermo Scientific, Rodano, Italy). Peptides were separated by a reverse phase column (Accucore^™^ RP-MS 100 × 2.1 mm, particle size 2.6 µm). The column was maintained at a constant temperature of 40 °C at a flow rate of 0.200 mL/min. Mobile phase A and B were water and acetonitrile, respectively, both acidified with 0.1% formic acid. The analysis was performed using the following gradient: 0–5 min from 2 to 5% B; 5–55 min from 5 to 30% B; 55–61 from 30 to 90% B and hold for 1 minute, at 62.1 min the percentage of B was set to the initial condition of the run at 2% and hold for about 8 min to re-equilibrate the column, for a total run time of 70 min. The mass spectrometry analysis was performed in positive ion mode. The ESI source was used with a voltage of 2.8 kV. The capillary temperature, sheath gas flow, auxiliary gas and spare gas flow were set at 325 °C, 45 arb, 10 arb and 2, respectively. S-lens was set at 70 rf. For the acquisition of spectra, a data-dependent (ddMS2) top 10 scan mode was used. Survey full-scan MS spectra (mass range m/z 381 to 1581) were acquired with resolution *R* = 70,000 and AGC target 3 × 106. MS/MS fragmentation was performed using high-energy c-trap dissociation (HCD) with resolution *R* = 35,000 and AGC target 1 × 106. The normalized collision energy (NCE) was set to 30. The injection volume was 3 μL. The acquired raw MS data files were processed and analyzed using Proteome Discoverer (v3.0.0.757, Thermo Fisher Scientific). SequestHT was used as a search engine and the following parameters were chosen. Database: Homo sapiens (Uniprot, downloaded on 01-02-2022); enzyme: trypsin; max. missed cleavage sites: 2; static modifications: carbamidomethyl (C); dynamic modifications: oxidation (M); precursor mass tolerance: 10 ppm; fragment mass tolerance: 0.02 Da. Only peptides and proteins with FDR value < 0.01 were reported. Abundance of identified peptides was determined by label-free quantification (LFQ) using match between runs. Statistical analyses and t test were performed on protein abundances using MetaboAnalyst software (https://www.metaboanalyst.ca/). Modulated proteins were analyzed through IPA (Ingenuity Pathway Analysis, QIAGEN).

### Fura-2 Ca^2+^ imaging

Control and TSC astrocytes, grown onto 24 mm round coverslips (3 × 10^4^ cell/coverslip), were loaded with 2.5 μM Fura-2/AM (Cat. No. F1201, Life Technologies, Milan, Italy) in the presence of 0.005% Pluronic F-127 (Cat. No. P6867, Life Technologies) and 10 μM sulfinpyrazone (Cat. S9509, Sigma-Aldrich, St. Louis, MO, USA) in complete astrocytes media. After loading (30 min in the dark) cells were washed once with KRB solution (125 mM NaCl, 5 mM KCl, 1 mM Na_3_PO_4_, 1 mM MgSO_4_, 5.5 mM glucose, 20 mM HEPES, pH 7.4) and allowed to de-esterify for 30 min. After this, the coverslips were mounted in an acquisition chamber and placed on the stage of a Leica DM6000B epifluorescence microscope equipped with a S Fluor 40 × /1.3 objective. Cells were alternatively excited at 340/380 nm by the monochromator Polichrome V (Till Photonics, Munich, Germany) and the fluorescent signal was collected by a Hamamatsu cooled CCD camera through bandpass 510/20 nm filter. The fluorescent signals were captured by MetaFluor software (Molecular Devices, Sunnyvale, CA, USA). The cells were stimulated with 200 μM ATP, 200 μM (S)-3,5-Dihydroxyphenylglycine (DHPG) and 200 μM Glutamate to detect cytosolic Ca^2+^. Separate experiments were performed to measure SOCE. Changes in cytosolic Ca^2+^ were monitored upon depletion of the intracellular Ca^2+^ stores. Experiments were carried out during exposure of the cells to the Ca^2+^-free solution. In the absence of Ca^2+^, the intracellular Ca^2+^ stores were depleted by tert-Butylhydroquinone (TBHQ, 50 µM; Sigma-Aldrich) treatment. Re-addition of 2 mM Ca^2+^ allowed assessment of the SOCE. Baseline values are expressed as mean ± SEM of 340/380 Fura-2 ratio values (referred to as “Fura ratio”). For comparison of Ca^2+^ dynamics, measured as an amplitude of Ca^2+^ increase from the baseline level, Fura-2 ratio values were normalized using formula (F_i_–F_0_)/F_0_ (referred to as “Normalized (Norm.) Fura Ratio”).

### Primary cultures transfection with mitochondrial Ca^2+^ indicator

Mitochondrial Ca^2+^ dynamics was monitored with 4mtD3cpv, a genetically encoded Ca^2+^ indicator belonging to the class of cameleons, in which Ca^2+^-responsive elements, such as CaM, alter the efficiency of fluorescence resonance energy transfer (FRET) between two fluorescent proteins [70, 75]. The transfection mixture consisted of Lipofectamine 2000 (Thermo Fisher Scientific, Cat. 11,668-019) and the 4mtD3cpv in 1:1 ratio, diluted in 1 ml Opti-MEM (Gibco, Cat. 11,058-021). Prior to transfection, control and TSC astrocytes were cultured to 80% confluency, detached and resuspended in transfection media containing complete astrocyte media and transfection mixture at a 1:1 ratio. Cell suspension was then seeded onto 24 mm glass coverslips (3 × 10^4^ cells/coverslip) placed in 6-well plates. Following seeding, the cells were incubated with the transfection media for three hours at 37 °C and 5% CO_2_. Subsequently, the media was replaced with complete astrocyte medium.

### Mitochondria Ca^2+^ imaging

After 24 h, coverslips with control and TSC astrocytes were washed with KRB solution (125 mM NaCl, 5 mM KCl, 1 mM Na_3_PO_4_, 1 mM MgSO_4_, 5.5 mM glucose, 20 mM HEPES, pH 7.4) and transferred to a suitable imaging acquisition chamber and mounted on the stage of a Leica DM6000B microscope. Samples were illuminated at 420 nm using a Polychrome V monochromator (Till Photonics, Munich, Germany) and fluorescent images at 475 nm (donor, ECFP) and 530 nm (acceptor, circularly permuted (cp) Venus) were simultaneously acquired using a Photometrics DV2 dual imager (Teledyne Photometrics, Tucson, US) and a cooled CCD camera (Hamamatsu, Japan). CpVenus/ECFP ratio was calculated online using MetaFluor software (Molecular Devices). After acquisition of a basal Ca^2+^ levels (first 30 s of acquisition), the cells were stimulated subsequently with 200 μM ATP and 5 μM ionomycin to detect basal mitochondrial Ca^2+^ (first 30 s of acquisition), Ca^2+^ influx in the mitochondria after ATP stimulation and Ca^2+^ efflux from the mitochondria after ionomycin stimulation. Imaging was performed using a Leica epifluorescence microscope equipped with a S Fluor 40 × /1.3 objective. Regions of interest (ROIs) were defined around individual mitochondria to measure changes in fluorescence intensity representing Ca^2+^ transients.

### Mitochondrial membrane potential determination

JC-1 dye (JC-1; Cayman, Ann Arbor, Michigan, USA) was used according to manufacturer’s instructions to assess mitochondrial membrane potential (Δψm) [29, 36]. Control and TSC astrocytes were resuspended in complete astrocyte media to final cell density of 1 × 10^6^ cells. JC-1 dye was added to the cell suspension at a final concentration of 1 µg/µl and incubated for 15 min at 37 °C and 5% CO_2_ to better discriminate the different sub populations. A negative control sample was prepared by adding an equal volume of vehicle control to the cell suspension. After washing with warm PBS, the sample was immediately loaded on flow cytometer (Accuri C6 Plus BD 660517). The fluorescence was measured with excitation wavelength at 485 nm, dual emission filters at 529 and 590 nm, and cut-off at 515 nm. 10,000 events were acquired for each sample using the following parameters: forward scatter (FSC), side scatter (SSC), PE (red, JC-1 aggregate) and FITC (green, JC-1 monomer) fluorescence channels. Gating strategies were applied to exclude cell debris and doublets based on FSC and SSC properties. The mitochondrial membrane potential was determined by the ratio of JC-1 aggregates (red fluorescence) to monomers (green fluorescence). Flow cytometry data analysis was performed using FlowJo software v10. Gated events were plotted on a bivariate dot plot to analyze the distribution of JC-1 fluorescence.

### High-resolution respirometry—OROBOROS

Cellular respiration rates in real-time of control and TSC astrocyte was measured using an Oroboros oxygraph-2 K high-resolution respirometer (Oroboros Instruments, Innsbruck, Austria). The “substrate, uncoupler, inhibitor, titration” (SUIT) protocol, specifically SUIT-003_O2_ce_D012, was employed following the guidelines recommended by the Oroboros manufacturer, as previously described [82]. Control and TSC astrocytes were detached from the flask using trypsin–EDTA (Gibco, Cat. 25,200,056), counted using a hemocytometer, and resuspended in pre-warmed respiration medium MiR05 (0.5 mM EGTA, 3.0 mM MgCl_2_, 60 mM potassium lactobionate, 20 mM taurine, 10 mM KH_2_PO_4_, 20 mM HEPES, 110 mM sucrose, 1 g/L bovine serum albumin, pH 7.1) to achieve a final cell density of 1 × 10^6^ cells/ml. Control astrocytes and TSC astrocytes were analyzed simultaneously in the neighboring chambers and oxygen concentration and flux were recorded using DatLab software (Oroboros). Baseline oxygen consumption rates were identified for each chamber during the “Routine” phase without and with pyruvate (5 mM) stimulation, used to sustain TCA-linked respiration in MiR05 medium. Subsequently, oligomycin (5 nM) was added to inhibit ATP synthase and assess uncoupled respiration (“Leak” phase). The protonophore carbonyl cyanide p-(trifluoro- methoxy) phenylhydrazone (FCCP) was then titrated (0.05 μM increments) until peak oxygen flux was achieved, indicative of maximal respiration [“Electron transport (ET)” phase]. Finally, 1 μl each of rotenone (0.5 μM) and antimycin A (2.5 μM) were sequentially added to inhibit ETC complexes I and III, respectively, and identify ET-independent respiration (ROX phase). Rates of O_2_ consumption (flux) were normalized to total protein content. Briefly, at the end of the experimental procedure, the cellular suspension from the two chambers was centrifuged at 1,000 × g for 5 min. The cellular pellet was lysed in 200 µL of lysis buffer (10 mM HEPES, 60 mM KCl, 1 mM EDTA, 0.075% NP40, 1 mM DTT) and then centrifuged at 15,000 × g for 15 min at 4 °C. The concentration of the protein in the supernatant was measured with Bradford Reagent.

### Electron microscopy (EM)

Frozen tissues were fixed by immersion in a mixture of 4% paraformaldehyde and 0.5% glutaraldehyde in 0.1 M phosphate buffer (PB) at pH 7.4 and stored at 4 °C. For electron microscopy, selected vibratome sections underwent a mild ethanol pre-treatment with concentrations of 10%, 25%, and 10% (5 min each) to enhance the penetration of immunoreagents. These unreacted sections were then postfixed for 10 min in 2.5% glutaraldehyde in PB, washed in PB, and postfixed for 1 h in 1% OsO_4_. All sections were dehydrated and cleared in propylene oxide, followed by flat-embedding in Epon-Spurr447R between acetate sheets (Aclar; Ted Pella, Redding, CA). The embedded sections were polymerized at 60 °C for 36 h. After polymerization, the embedded sections were examined under a dissecting microscope, and areas of interest were excised using razorblades and affixed to cured resin blocks. Semithin (1 mm-thick) sections were cut with a Reichert ultramicrotome and collected on glass slides with or without toluidine blue counterstaining for light microscopic inspection. Ultrathin sections from unreacted samples were counterstained with uranyl acetate and lead citrate, while sections from immunoreacted samples were counterstained with lead citrate only or left unstained. All sections were examined using a Zeiss 902 electron microscope. Identification of profiles was based on established morphologic criteria. This methodology was employed to prepare and examine the specimens for electron microscopy, allowing for the detailed characterization and quantification of mitochondria based on their morphologic features. Temporal Lobe Epilepsy (TLE) tissue, with healthy-appearing astrocytes and neurons, was utilized as a control to enable a comprehensive qualitative and quantitative assessment of mitochondrial morphology in comparison to TSC tissue and to avoid post-mortem artifacts. The clinical details of the specimens utilized for EM are reported in Online Resource 1.

### Analysis

Statistical analysis was performed with GraphPad Prism software (Graphpad software Inc., La Jolla, CA) using the non-parametric Mann–Whitney *U* test or, for multiple groups, the non-parametric Kruskal–Wallis test followed by Mann–Whitney *U* test. An *p* value < 0.05 was considered statistically significant.

## Results

### Differential expression of genes related to Ca^2+^ signal pathways in TSC

#### Section 1: quality control of RNA sequencing of TSC brain tissue

In the present study, we conducted RNA-Seq analysis of datasets consisting of tissue samples from TSC cortical tuber (*n* = 21) and age- and tissue-matched control cortex tissues (*n* = 15) to elucidate differentially expressed genes, enriched pathways, and putative molecular mechanisms associated with TSC.

A PCA was performed to explore the underlying dimensions on which most variance is observed within the dataset, specifically examining whether the variance of the data was driven by the diagnosed pathology and/or the area of resection. The scatter plots of the samples based on the scores of PC1 and PC2 for pathology diagnosed (Online Resource 3a), and area of resection (Online Resource 3b) did not exhibit distinct divergent clustering patterns.

#### Section 2: differential expression and GO pathway analysis

Exploring the transcriptional profile of TSC through differential expression analysis revealed significant alterations in gene expression between TSC and control cortex. A total of 3772 genes exhibited statistically significant differential expression (Log_2_FC ≥  ≤  ± 0.5; p.adj < 0.05), including 2104 upregulated genes and 981 downregulated genes (Online Resource 3c).

**Fig. 1:**
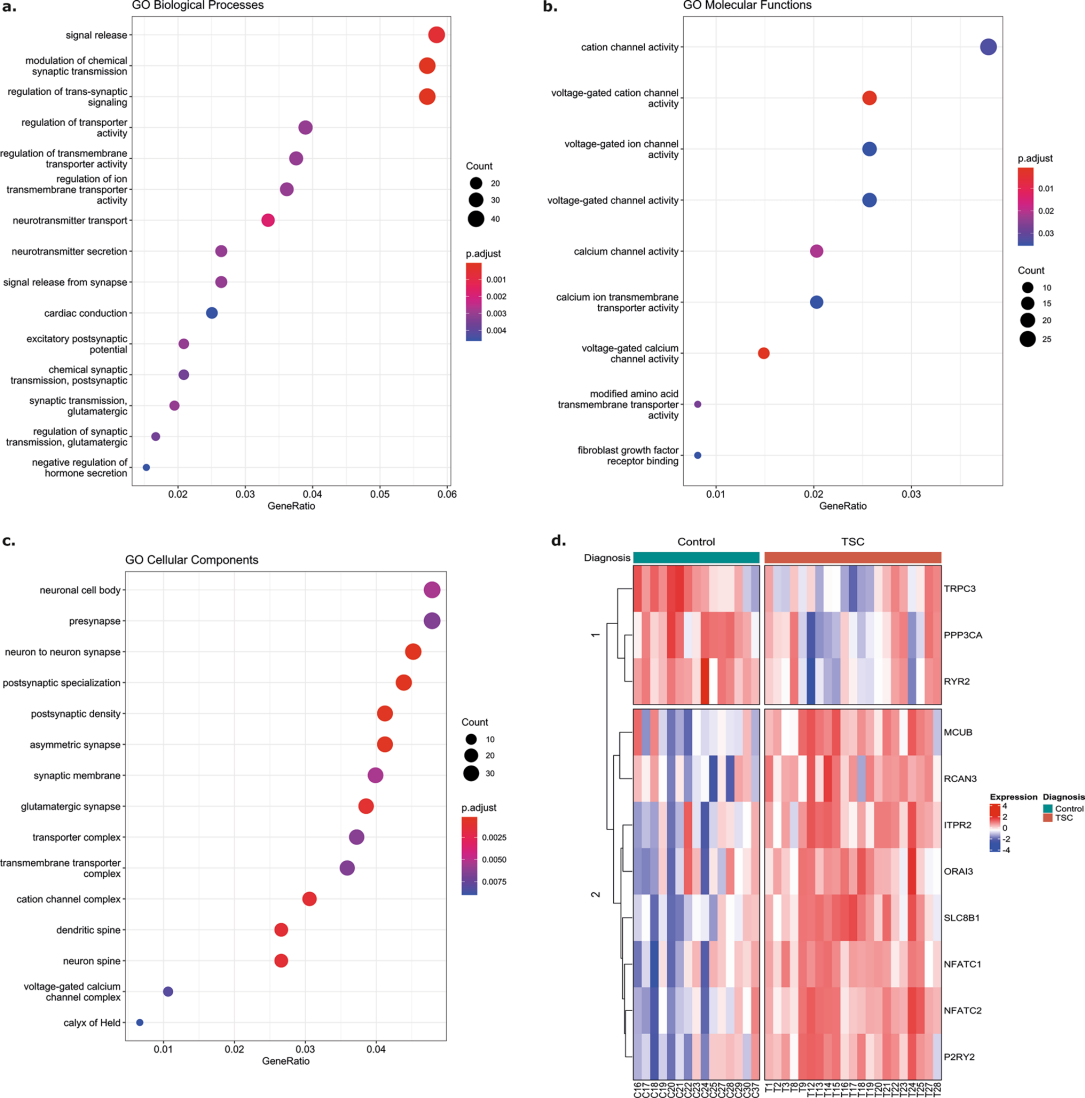
Bulk RNASeq analysis of control cortex and TSC cohort. **a**. Visualization of the significantly enriched GO Biological Processes (BP) pathways (p.adj < 0.05) among the 981 downregulated genes. The size of each point is proportional to the number of differentially expressed genes present in the pathway, while the intensity of the color indicates the p.adj of the pathway enrichment. **b.** Visualization of the significantly enriched GO Molecular Function (MF) pathways (p.adj < 0.05) among the 981 downregulated genes. The size of each point is proportional to the number of differentially expressed genes present in the pathway, while the intensity of the color indicates the p.adj of the pathway enrichment. **c.** Visualization of the significantly enriched GO Cellular Components (CC) pathways (p.adj < 0.05) among the 981 downregulated genes. The size of each point is proportional to the number of differentially expressed genes present in the pathway, while the intensity of the color indicates the p.adj of the pathway enrichment. **d.** Supervised clustering heatmap visualizing the expression of the significant Ca^2+^-related genes in control tissues and TSC cortical tubers. Adjusted *p* value: *p.adj ≤ 0.05; **p.adj ≤ 0.01; ** p.adj ≤ 0.001; ****p.adj ≤ 0.0001

To gain insights into the functional role of the 3772 differentially expressed genes identified in our study, a Gene Ontology (GO) pathway enrichment analysis was performed on the upregulated and downregulated sets of genes separately. Among the 2104 upregulated genes, 764 GO Biological Processes (GO_BP), 43 GO Molecular Function (GO_MF), and 91 GO Cellular Component (GO_CC) terms exhibited significant enrichment (Online Resource 4, 5 and 6). The analysis revealed that most significantly enriched pathways include neutrophil activation and degranulation involved in immune response, I-kappaB kinase/NF-kappaB signaling, positive regulation of cytokine production, T cell activation, gliogenesis and glial cell differentiation supporting the strong inflammatory phenotype and astrogliosis observed in TSC. Subsequently, a GO pathway enrichment analysis was performed on the 981 downregulated genes. Enriched terms within GO_BP included pathways involved in neurotransmitter secretion and transport, regulation of membrane potential, and regulation of cation channel activity (Fig. 1a, shows the top 20 enriched pathways). The enriched terms within GO_MF involved in voltage-gated Ca^2+^ channel activity, mitochondrial protein-containing complex, and neurotransmitter receptor complex (Fig. 1b shows the top nine enriched pathways). The enriched compartments (GO_CC) among the downregulated genes involved mitochondrial protein-containing complex, postsynaptic density, ion channel complex and Ca^2+^ channel complex (Fig. 1c shows the top 20 enriched pathways). The complete list of significantly enriched GO pathways is reported in Online Resource 7, 8, and 9.

#### Section 3: differential expression of genes related to Ca^2+^ signaling pathways

The GO pathway analysis of differentially expressed genes in TSC provided valuable insights into their functional implications, emphasizing the potential involvement of cellular respiration, mitochondria, Ca^2+^ regulation, and neurotransmitter signaling. To further investigate these findings, we examined the expression of specific genes related to Ca^2+^ homeostasis, including Ca^2+^ buffers, transducers, regulators, and channels, as well as genes involved in mitochondrial and ER Ca^2+^ homeostasis, Ca^2+^ regulated inflammation, and G-alpha-q/11 coupled GPCRs (Online Resource 10). These investigations aimed to gain a deeper understanding of the molecular mechanisms underlying the observed functional alterations in TSC (Fig. 1d; Online Resource 3d). None of the genes, involved in the general Ca^2+^ homeostasis, were differentially expressed in TSC compared to controls, however the expression of multiple genes encoding for proteins involved in Ca^2+^ release from the ER and SOCE were altered. *ITPR1* and *ITPR2* encode for two of the Inositol 1,4,5-Trisphosphate Receptor Type 1 and 2 (IP_3_R1 and IP_3_R2) and the latter was upregulated (Log_2_FC = 1.25; p.adj = 0.001). IP_3_Rs play a crucial role in mediating Ca^2+^ release from the ER in response to inositol-1,4,5-trisphosphate-mediated signaling, thereby generating cytoplasmic Ca^2+^ signals and facilitating Ca^2+^ influx into the mitochondrial matrix to regulate oxidative metabolism and cell survival [10, 33, 85]. Ca^2+^ release from the ER into the cytoplasm is mediated by Ryanodine Receptor 1–3 (RyR1-3), a Ca^2+^ channel encoded by *RYR1* (not differentially expressed), *RYR2* (Log_2_FC = − 0.65; p.adj = 0.015), and *RYR3* (Log_2_FC = 0.64; p.adj = 0.045)*.* In TSC, differential expression of these genes combined with the altered expression of IP_3_R, suggests an impairment in the regulatory mechanism controlling Ca^2+^ release from the ER [92].

The mechanism regulating Ca^2+^ entrance in the cytoplasm is mediated by the ORAI Calcium Release-Activated Calcium Modulator 1–3 (*ORAI1, ORAI2, ORAI3*), a membrane Ca^2+^ channel that is activated by the Ca^2+^ sensor STIM1-2 (*STIM1, STIM2*) when Ca^2+^ stores are depleted [58]. Furthermore, Ca^2+^ ions influx in the cytoplasm is also mediated by Transient Receptor Potential Cation Channel Subfamily C Member 1, 3, and 4 (*TRPC1*, *TRPC3*, *TRPC4*), a membrane protein that can form a non-selective channel permeable to Ca^2+^ [58]. From the differential expression analysis, the data suggest a mild alteration at transcriptional level of the mechanism of Ca^2+^ entry in the cytoplasm and subsequently in the ER as only *TRPC3* (Log_2_FC = -0.65; p.adj = 0.037) and *ORAI3* (Log_2_FC = 0.71; p.adj = 0.013) expression was impaired. In contrast to the possible alteration in the SOCE, genes involved in the [Ca^2+^]_ER_ homeostasis such as calreticulin (*CALR*) and calnexin (*CANX*) were not differentially expressed. In the mitochondria, Ca^2+^ uptake occurs through voltage-dependent anion-selective channel proteins (VDAC1) on the outer mitochondria membrane (OMM) and the mitochondrial Ca^2+^ uniporter (MCU, MICU1, MICU2, MICU3, MCUB) complex localized on IMM [41]. Subsequently, Ca^2+^ is released back from the mitochondrial matrix to the intermembrane space (IMS) via NCLX (*SLC8B1*) and Leucine zipper EF-hand- containing transmembrane protein 1 (LETM1). Our transcriptomic data showed the upregulation of *MCUB* (Log_2_FC = 1.38; p.adj = 0.001) and *SLC8B1* (Log_2_FC = 0.82; p.adj = 0.0001) suggesting a reduction in Ca^2+^ entry into the mitochondria with a potential increase in Ca^2+^ efflux, resulting in reduced-Ca^2+^ dynamics in the mitochondrial matrix.

Further, the expression of most relevant genes encoding for Ca^2+^ buffer regulators was compromised. Protein Phosphatase 3 Catalytic Subunit Alpha (*PPP3CA,* Log_2_FC = − 0.72; p.adj = 0.026) enables several functions, including ATPase binding activity, CaM binding activity, and CaM -dependent protein phosphatase activity, calcineurin-NFAT signaling cascade, peptidyl–serine dephosphorylation and response to Ca^2+^ ion. *RCAN3* (Log_2_FC = 0.73; p.adj = 0.004) encodes for RCAN Family Member 3 and is involved in Ca^2+^-mediated signaling, inhibiting calcineurin-dependent transcriptional responses by binding to the catalytic domain of calcineurin A [72, 76].

Given the established involvement of the immune response in the epileptogenesis of TSC corroborated by the significant enrichment of inflammatory response pathways in our study, the expression of genes associated with Ca^2+^ regulated inflammation was investigated. Nuclear Factor of Activated T Cells 1–4 (*NFATC1, NFATC2, NFATC3, NFATC4*) are DNA-binding transcription complexes which play a central role in inducible gene transcription during immune response. The upregulation of *NFATC1* (Log_2_FC = 1.66; p.adj = 0.001), *NFATC2* (Log_2_FC = 1.8; p.adj = 4.233e–05) suggests a possible interplay between the impairment of Ca^2+^ pathways and inflammatory response in TSC.

Purinergic Receptor P2Y2 (P2RY2) is a G protein-coupled receptor that triggers intracellular Ca^2+^ release and influx, leading to changes in cytosolic Ca^2+^ concentration. Activation of P2RY2 can trigger Ca^2+^ influx from the extracellular space, mediated by TRPC and further modulate Ca^2+^ entry pathways, such as SOCE [108]. In our TSC cohort, *P2RY2* was upregulated with a Log_2_FC = 3.35 and p.adj = 0.0004, suggesting increased intracellular Ca^2+^ mobilization.

In conclusion, the comprehensive transcriptional analysis of TSC unveiled substantial alterations in pathways associated with cellular respiration, ER and mitochondria, Ca^2+^ regulation, and neurotransmitter signaling, in addition to the well-established dysregulated immune response in TSC. Specifically, the expression of genes involved in general Ca^2+^ homeostasis indicated a potential impairment in Ca^2+^ handling. Furthermore, the dysregulation of SOCE revealed a disturbance in Ca^2+^ entry mechanisms, while the impairment in the regulatory mechanism controlling Ca^2+^ release from the ER suggested an imbalance in intracellular Ca^2+^ dynamics accompanied by alterations in the Ca^2+^ influx and efflux mechanisms in the mitochondria. In addition, our data indicated the possible interplay between disturbances in Ca^2+^ signaling and inflammatory response in TSC. Collectively, these findings contribute to our understanding of the complex Ca^2+^ dysregulation and its potential involvement in the pathophysiology of TSC.

#### Section 4: differential expression of genes related to Ca^2+^ signaling pathways in astrocytes

**Fig. 2:**
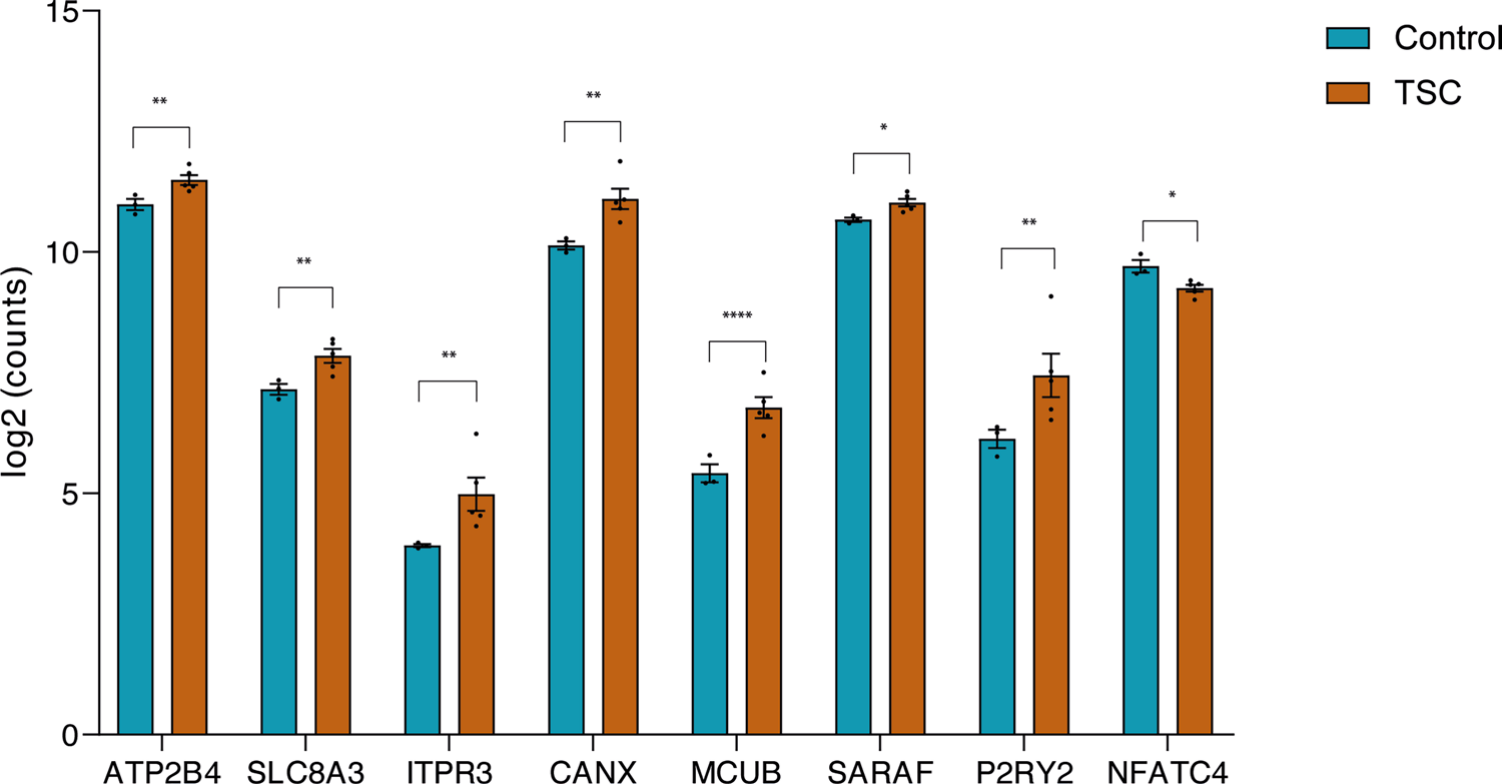
Differential expression of calcium related genes using single-cell RNA sequencing. Control and TSC tissues were sequenced using the 10 × genomics Chromium Single Cell Gene Expression Flex protocol. From all cells, astrocytes were extracted computationally, and differential expression analysis was performed using DESeq2 between control (n = 3) and TSC (n = 5) samples. Data are expressed as mean ± SEM. Adjusted *p* value: *p.adj ≤ 0.05; **p.adj ≤ 0.01; ****p.adj ≤ 0.0001)

In the field of epilepsy research, investigations into Ca^2+^ signaling dynamics have predominantly concentrated on neurons, providing insights into the complexities of neuronal dysfunction. Bulk sequencing approaches have been utilized in revealing alteration in Ca^2+^-related processes within these neuronal population and tissue of patients as mentioned in Sect. 3. However, to increase our knowledge on epilepsy and seizures, we should investigate beyond just neurons and consider a wider perspective. Astrocytes play an important role in maintaining brain homeostasis, but despite their significance, the exploration of Ca^2+^signaling in astrocytes has remained less explored in the context of epilepsy. Therefore, by investigating these glial cells, we aim to unveil more specific glial dysregulation of calcium signaling, shedding light on the contribution of astrocytes in epilepsy. Using single-cell RNA sequencing (scRNA-seq), we identified seven different cell types in our frontal cortex samples (Online Resource 11). To investigate the Ca^2+^signaling in astrocytes, we extracted the astrocytes from the dataset, and we looked at the expression profiles of Ca^2+^-related genes. By comparing control (*n* = 3) and TSC (*n* = 5) samples, we identified eight differentially expressed genes that were related to calcium signaling as mentioned in Sect. 3 (Fig. 2). *ATP2B4* (Log_2_FC = 0.789; p.adj = 0.0229), *SLC8A3* (Log_2_FC = 1.232; p.adj = 0.006), *ITPR3* (Log_2_FC = 3.641; p.adj = 0.003), *CANX* (Log_2_FC = 1.636; p.adj = 0.002), *MCUB* (Log_2_FC = 2.907; p.adj = 1,55E-05), *SARAF* (Log_2_FC = 0.568; p.adj = 0.037) and *P2RY2* (Log_2_FC = 2.897; p.adj = 0.005) were found to be upregulated, while *NFATC4* (Log_2_FC = − 0.699; p.adj = 0.022) was found to be downregulated in TSC.

### Proteomics

**Fig. 3:**
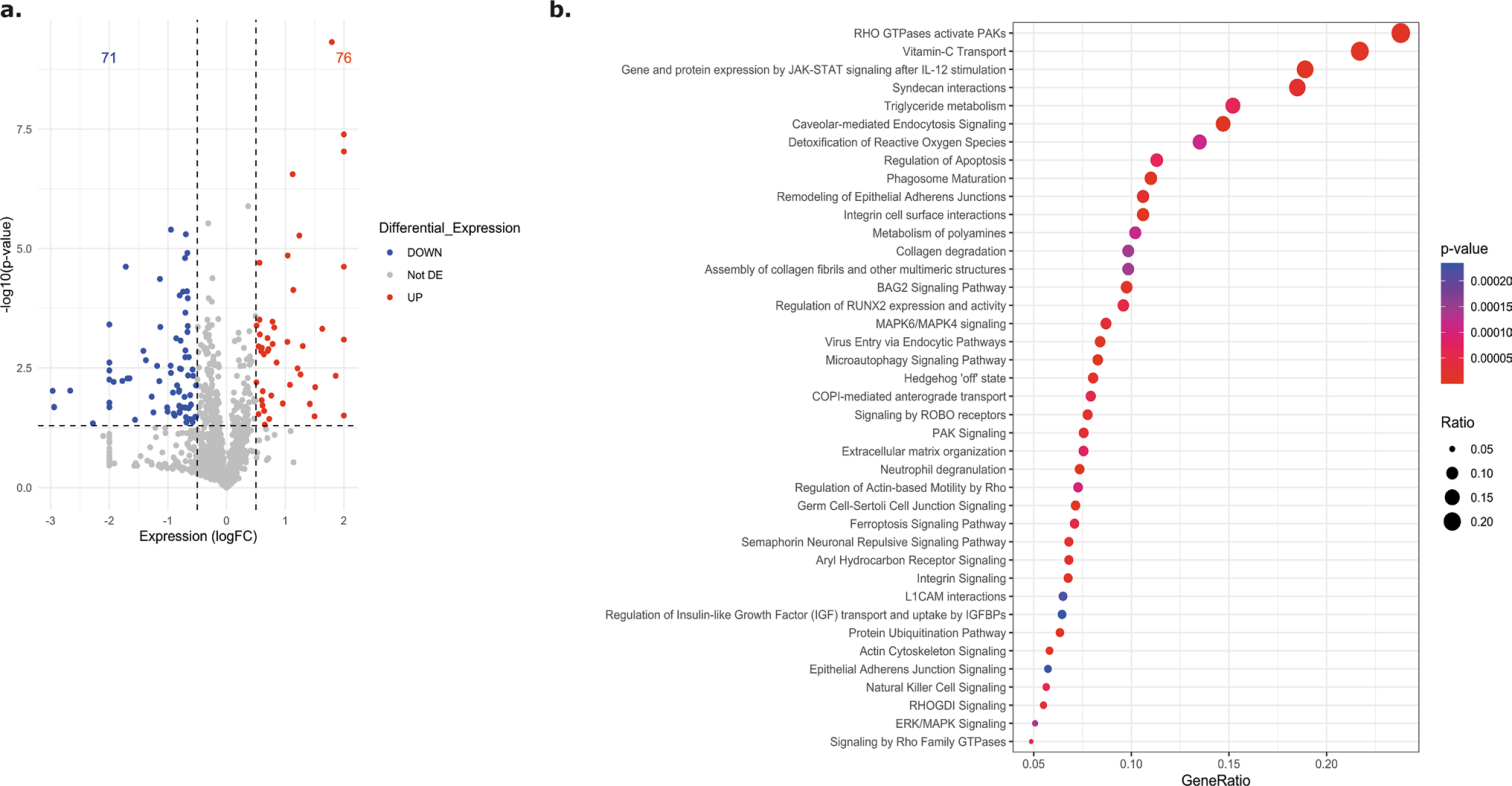
Ingenuity pathway analysis (IPA) on control and TSC primary astrocytes cultures. **a.** Volcano plot showing the differentially expressed proteins (DEPs) (*p* value ≤ 0.05) between control (*n* = 6) and TSC (*n* = 6) primary culture astrocytes. A total of 76 proteins were found to be upregulated and 71 downregulated in TSC compared to control astrocytes. **b.** Top-ranked 40 enriched canonical pathways predicted by IPA (*p* value ≤ 0.05). The size of each point is proportional to the number of differentially expressed proteins present in the pathway, while the intensity of the color indicates the *p* value of the predicted canonical signaling pathways

We conducted transcriptomic analysis at both bulk and single-cell levels; however, recognizing the potential disparities between transcriptomic and proteomic profiles, we aimed to complement our finding by performing proteomic analysis specifically in primary astrocyte cultures. Previous studies were conducted to verify the maturity of our control astrocytes. Despite being derived from younger individuals compared to the TSC astrocytes, they still exhibited a higher level of maturity[62] and similar levels of maturity to cultures used in previous studies obtained from adult individuals[5, 6]. Control and TSC primary astrocyte were cultured to conduct proteomic analysis to elucidate protein abundance and enriched pathways associated with TSC. LogFoldChange ≥  ≤  ± 0.5 and *p* value ≤ 0.05 were used as the screening criteria for significantly differentially expressed proteins (DEPs). A total of 1660 proteins were quantified with the mass spectrometry analysis (Online Resource 12) among which 147 were differentially expressed, 76 upregulated while 71 downregulated in TSC compared to controls (Fig. 3a). The functional significance of all identified DEPs in the TSC primary cultures was explored with the ingenuity pathway analysis (IPA) that predicted a total 705 canonical pathways (Online Resource 13) of which 209 were significantly enriched. Among these, ten were predicted activated (positive zScore), while 67 were predicted inhibited (negative zScore). The remaining pathways had zero activity pattern predicted (zScore = 0, *n* = 6) or the activity pattern was not available (*n* = 113). Of relevance to TSC, we found enriched pathways involved in the extracellular matrix organization (ECM), immune response and cytokines signaling, autophagy, ferroptosis signaling pathway, Golgi-to-ER traffic, regulation of mitotic cell cycle, Slit/Robo pathways and collagen biosynthesis pathways (Fig. 3b, shows the top 20 enriched pathways). Lastly, the 1660 quantified proteins were next imported into the STRING database (STRING 12.0) to perform network interaction analysis of protein–protein relationships. The GO pathway analysis revealed strong enrichment of pathways involved in the calcium signaling and ATP metabolism (Online Resource 14).

### Reduced response to environmental changes and stimuli in TSC astrocytes

**Fig. 4:**
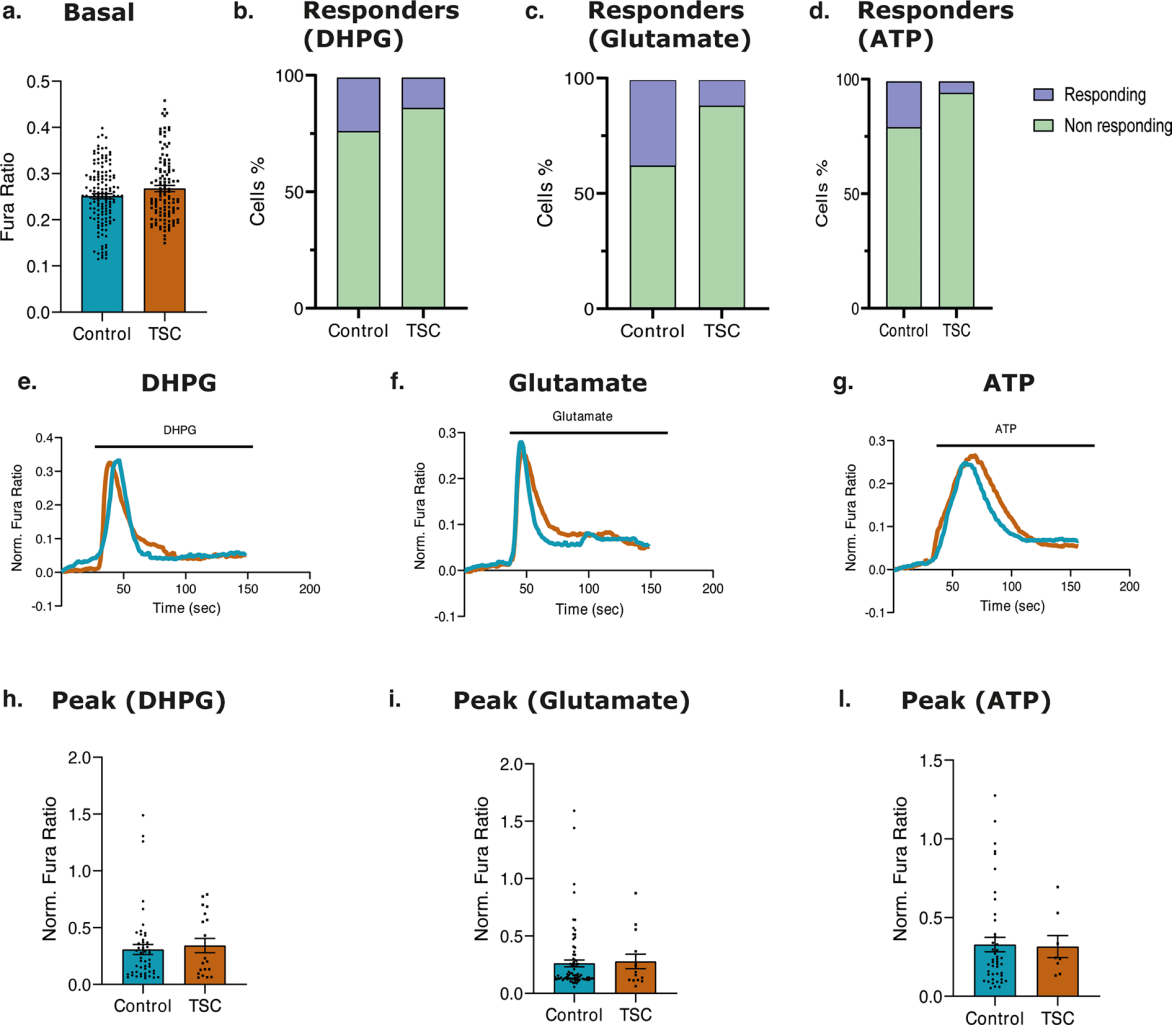
Ca^2+^ imaging in the cytoplasm of control and TSC astrocytes. Control and TSC astrocytes, previously loaded with Fura-2/AM, were stimulated with 200 μM DHPG, 200 μM Glutamate or 200 μM ATP in Ca^2+^-containing KRB solution (*n* = 216 control cells and *n* = 140 TSC cells, form 12 independent coverslip). **a.** No difference in basal Ca^2+^ level could be observed. **b. c. d.** The percentage of responding astrocytes (peak upon stimulation > 0.05), has been investigated, in both experimental group (light green: not responding cells; blue: responding cells). **e. f. g.** Representative curves of cytosolic Ca^2+^ responses upon the applied stimulus. The blue curve showed the Ca^2+^ response of control astrocytes, while the dark orange curve showed the Ca^2+^ response of TSC astrocytes. **h. i. l.** The amplitude of cytosolic Ca^2+^ response, upon DHPG, Glutamate and ATP stimulation has been measured, considering only responding cells in both control and TSC astrocytes. Data are expressed as mean ± SEM; *p* value: *p value ≤ 0.05; **p value ≤ 0.01; ***p value ≤ 0.001; ****p value ≤ 0.0001. Mann–Whitney *U* test

To explore the intracellular calcium dynamics in TSC we first explored the concentration of calcium in the cytosol of TSC primary astrocytes given the strong relationship between mTOR activation and CaM activity. Cytosolic Ca^2+^ responses upon stimulation were followed via Fura-2 probe, and no differences in basal [Ca^2+^]_c_ level were reported (Fig. 4a). Both control and TSC astrocytes have been challenged with: DHPG (200 µM), a selective agonist of group I mGluRs, glutamate (200 µM) and ATP (200 µM). Upon all the applied stimuli, TSC astrocytes displayed a reduced responsiveness compared to controls. Indeed, upon DHPG 28% of control astrocytes responded with increased [Ca^2+^]_c_ while the responding cells in TSC group were only the 20% (*p* value: 0.0354) (Fig. 4 b and e; Online Resource 15a). The difference in the percentage of responding cells is higher considering glutamate stimulation (31% control astrocytes; 11% TSC astrocytes, *p* value: 0.0135) (Fig. 4c, f; Online Resource 15b) and ATP stimulation (21% control astrocytes; 5% TSC astrocytes, *p* value: 0.0385) (Fig. 4d, 4g; Online Resource 15c). Taking into account only the responding cells in both the experimental groups, the [Ca^2+^]_c_ peak (Fig. 4h, i, l) was not significatively different upon the applied stimuli (DHPG; Glutamate and ATP), and neither the curve profiles displayed any difference (Fig. 4e–g).

Collectively, our findings suggest that TSC astrocytes exhibit a reduced ability to respond to stimuli, as evidenced by attenuated [Ca^2+^]_c_ responses, underscoring their reduced capacity to react to environmental changes.

Furthermore, TSC astrocytes exhibited a trend toward lower [Ca^2+^]_c_ compared to control astrocytes, although the difference did not reach statistical significance. These observations underscore the need for further investigations to elucidate the potential dysregulation of Ca^2+^ signaling pathways in TSC astrocytes and its implications in TSC pathophysiology.

### TSC astrocytes displayed dysregulation of ER Ca^2+^ release and SOCE impairment

SOCE is a fundamental cellular process that regulates Ca^2+^ influx from the extracellular space into the cytosol and subsequently into the ER upon [Ca^2+^]_ER_ depletion. During cellular homeostasis, the ER undergoes a continuous leakage of Ca^2+^ ions through numerous leak channels [53], a phenomenon consistently replenished by the activity of the SERCA protein [38, 66, 101]. In addition, the mTOR hyperactivation and the increased-ROS production in TSC impacts on the [Ca^2+^]_ER_, therefore in this study we explored ER Ca^2+^ handling and the ability to perform SOCE. To induce complete [Ca^2+^]_ER_ depletion, thus enabling assessment of its ability to empty, a specific SERCA inhibitor TBHQ was employed. This treatment led to concentration-dependent Ca^2+^ accumulation within the cytosol and [Ca^2+^]_ER_ stores depletion, and the consequent activation of STIM-ORAI protein interaction [87]. Subsequently, for quantifying ER replenishment capacity, Ca^2+^ stimulation was induced allowing the measurement of ER responsiveness to refilling demands.

**Fig. 5:**
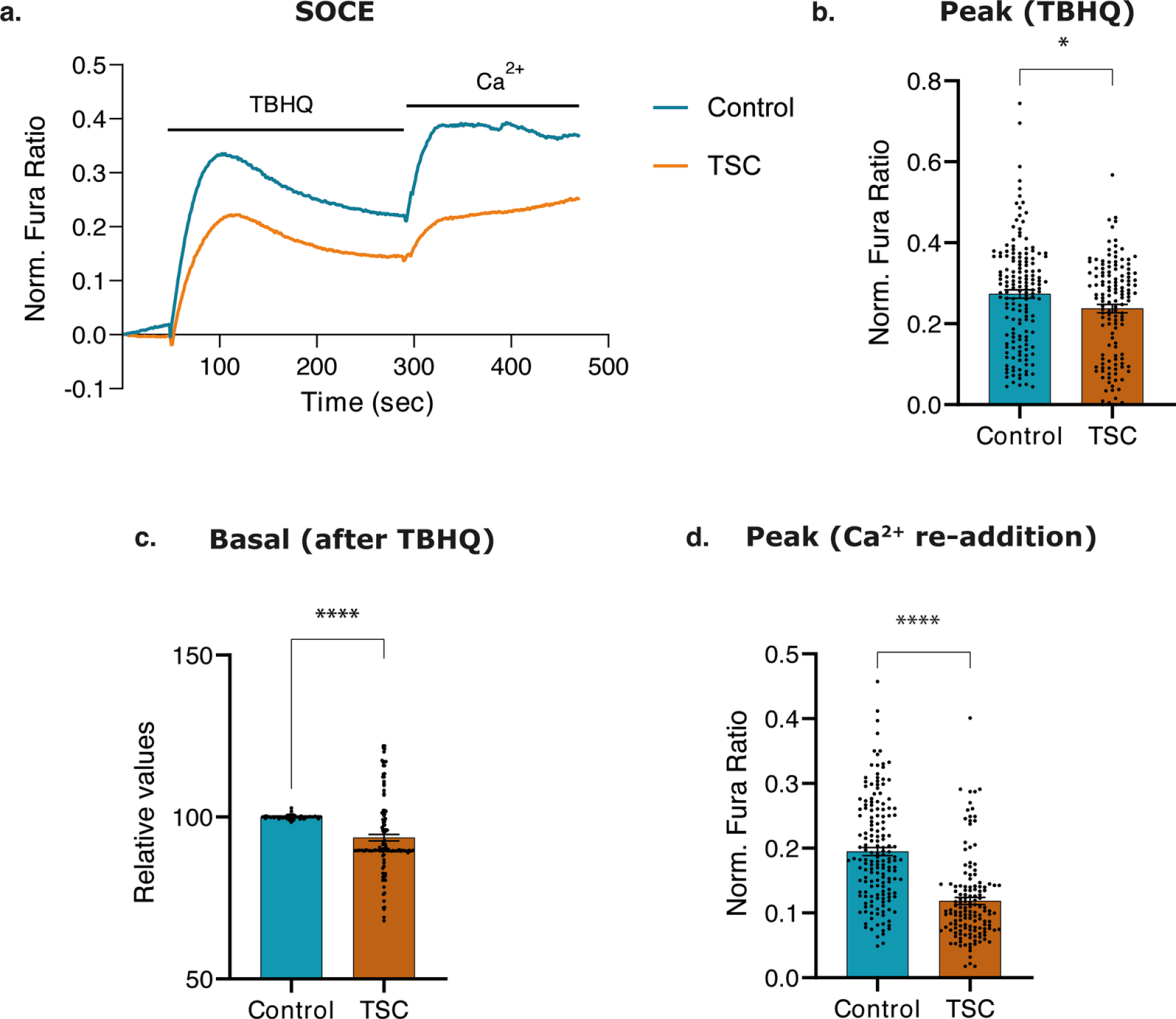
Ca^2+^ imaging in endoplasmic reticulum (ER) of control and TSC astrocytes. **a.** Control and TSC astrocytes, previously loaded with Fura-2/AM, were stimulated with TBHQ (50 s) and Ca^2+^ (300 s) in KRB solution. Representative curves of cytosolic Ca^2+^ responses upon the applied stimulus of three independent experiments. The blue curve showed the Ca^2+^ response of control astrocytes, while the dark orange curve showed the Ca^2+^ response of TSC astrocytes (*n* = 160 control cells and *n* = 136 TSC cells, from 14 independent coverslips). **b.** Bar plot visualizing significant reduction of Ca^2+^ concentration in the cytoplasm of TSC astrocytes compared to control astrocytes after blocking the SERCA protein with TBHQ stimuli (between 30 and 300 s). **c.** Bar plot visualizing basal cytosolic Ca^2+^ concentration in TSC astrocytes after TBHQ stimuli compared to controls. **d.** Bar plot visualizing significant reduction in the absolute concentration of Ca^2+^ in the ER of TSC astrocytes compared to control astrocytes after Ca^2+^ administration (at 300 s). Data are represented as mean ± SEM; *p* value: *p value ≤ 0.05; **p value ≤ 0.01; ***p value ≤ 0.001; ****p value ≤ 0.0001. Mann–Whitney *U* tests were used to evaluate significance

In this study, Fura-2 dye was used to measure [Ca^2+^]_c_, in zero-Ca^2+^ KRB solution (within the first 30 s), ER Ca^2+^ release ability (upon TBHQ stimulation, between 30 and 300 s) and assess SOCE (upon Ca^2+^ stimulation at 300 s) (Fig. 5a).

Upon treatment with 50 µM TBHQ, TSC astrocytes exhibited a significant reduction of Ca^2+^ release from the ER compared to control astrocytes (*p* value: 0.029 for the amplitude of [Ca^2+^]_c_, *p* value < 0,0001 for the baseline level after TBHQ treatment) (Fig. 5b, c), this suggests a reduction of [Ca^2+^]_ER_ due to reduced-Ca^2+^ transport into ER and/or an increase of [Ca^2+^]_ER_ leakage. Subsequently, cells were stimulated with Ca^2+^ to assess SOCE and TSC astrocytes displayed a decreased [Ca^2+^]_c_ influx (*p* value < 0.0001) (Fig. 5d), indicating an impairment of SOCE. Overall, the reduction in basal [Ca^2+^]_c_ levels, after SERCA blockade, and further Ca^2+^ stimulation suggest a dysregulation of release from the ER and SOCE in TSC astrocytes and might lead to a disruption in Ca^2+^ signaling and downstream Ca^2+^-dependent cellular mechanisms.

### Reduced response to stimuli and altered Ca^2+^ dynamics in TSC mitochondria

The alterations detected from the previous experiments in Ca^2+^ storage in the ER, suggest a potential impairment in the ability to store Ca^2+^ and its dynamics in the different organelles in TSC astrocytes. Control and TSC astrocytes were transfected with 4mtD3cpv Ca^2+^ indicator, enabling targeted Ca^2+^ imaging in the mitochondria matrix in single cell [75].

**Fig. 6:**
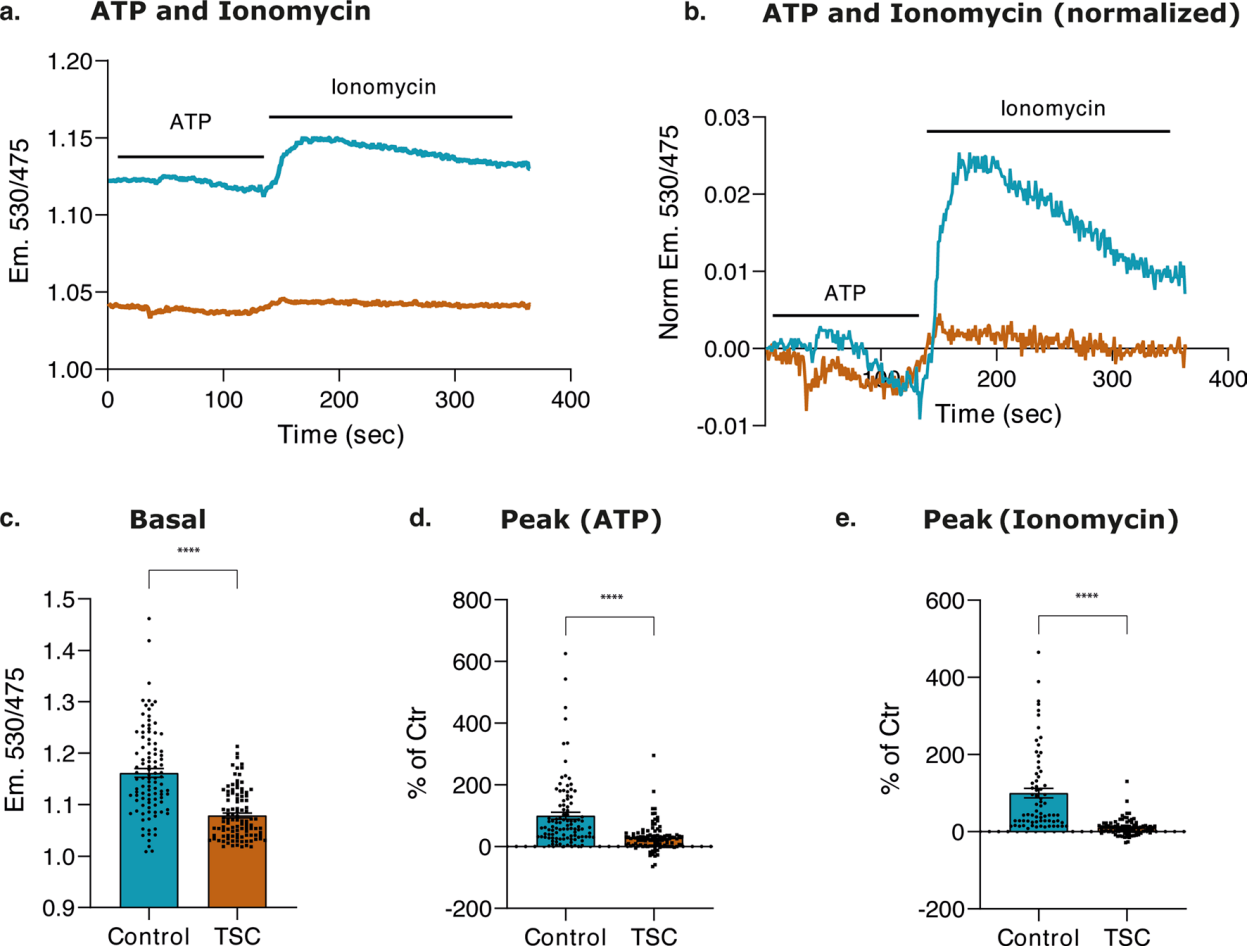
Mitochondrial Ca^2+^ imaging of D3-positively transfected mitochondria in control and TSC astrocytes. **a.** Representative curve of seven independent coverslips. Control and TSC astrocytes, previously transfected with D3-plasmid and stimulated with ATP and ionomycin in KRB solution. The blue curve showed the basal Ca^2+^ response of control astrocytes before and after ATP (30 s) and ionomycin (150 s) stimuli, while the dark orange curve showed the basal Ca^2+^ response of TSC astrocytes. **b.** Representative curves of seven independent coverslips. The basal Ca^2+^ levels were normalized. The blue curve showed the basal Ca^2+^ response of control astrocytes before and after ATP (30 s) and ionomycin (150 s) stimuli, while the dark orange curve showed the basal Ca^2+^ response of TSC astrocytes. **c.** The barplot showed the significant reduction concentration of mitochondrial Ca^2+^ in TSC astrocytes at homeostasis (at 50 s) compared to controls after ATP stimulation (*n* = 100 ROIs for control; *n* = 102 ROIs for TSC). **d.** The barplot showed the significant reduction of mitochondrial Ca^2+^ concentration in TSC astrocytes after ATP stimulation compared to control astrocytes (*n* = 100 ROIs for control; *n* = 102 ROIs for TSC). **e.** The barplot showed the significant reduction of mitochondrial Ca^2+^ concentration in TSC astrocytes after Ionomycin stimulation compared to control astrocytes (*n* = 70 ROIs for control n = 88 ROIs for TSC). Data are expressed as mean ± SEM; *p* value: *p value ≤ 0.05; **p value ≤ 0.01; ***p value ≤ 0.001; ****p value ≤ 0.0001. Mann–Whitney *U* tests were used to evaluate significance

A reduction in the basal [Ca^2+^]_m_ was observed in TSC astrocytes when compared to control astrocytes (*p* value < 0.0001) (Fig. 6a, b and c). Subsequently, stimulation with 200 μM ATP was used to assess the mitochondrial Ca^2+^ uptake. It was observed that mitochondria in TSC astrocytes exhibited a significant reduction in Ca^2+^ influx compared to control astrocytes (Fig. 6d). Because the reduced ER Ca^2+^ content (Fig. 6) might affect Ca^2+^ dynamics in mitochondria, we used a Ca^2+^ ionophore, ionomycin, to induce an artificial cytosolic Ca^2+^ overload to expose control and TSC mitochondria to the same Ca^2+^ concentrations, independent of the ER Ca^2+^ releasing capacity. Upon treatment with 5 µM ionomycin, we found a significantly reduced (*p* value < 0.0001) Ca^2+^ influx in the mitochondrial matrix of TSC compared with control astrocytes, suggesting an intrinsic impairment of mitochondrial Ca^2+^ transport system (Fig. 6e).

These findings are supported by the transcriptional profile of genes modulating mitochondrial Ca^2+^ homeostasis. The substantial reduction of Ca^2+^ influx in mitochondrial matrix may be attributed to the compromised activity of the mitochondrial Ca^2+^ uniporter, potentially influenced by the elevated expression of MCUB, dominant Negative Subunit of MCU complex. Furthermore, the diminished [Ca^2+^]_m_ may also stem from heightened Ca^2+^ efflux driven by the upregulation of NCLX localized on the IMM.

### Significant depolarization of mitochondria membrane potential (ΔΨ_m_) in TSC astrocytes

**Fig. 7:**
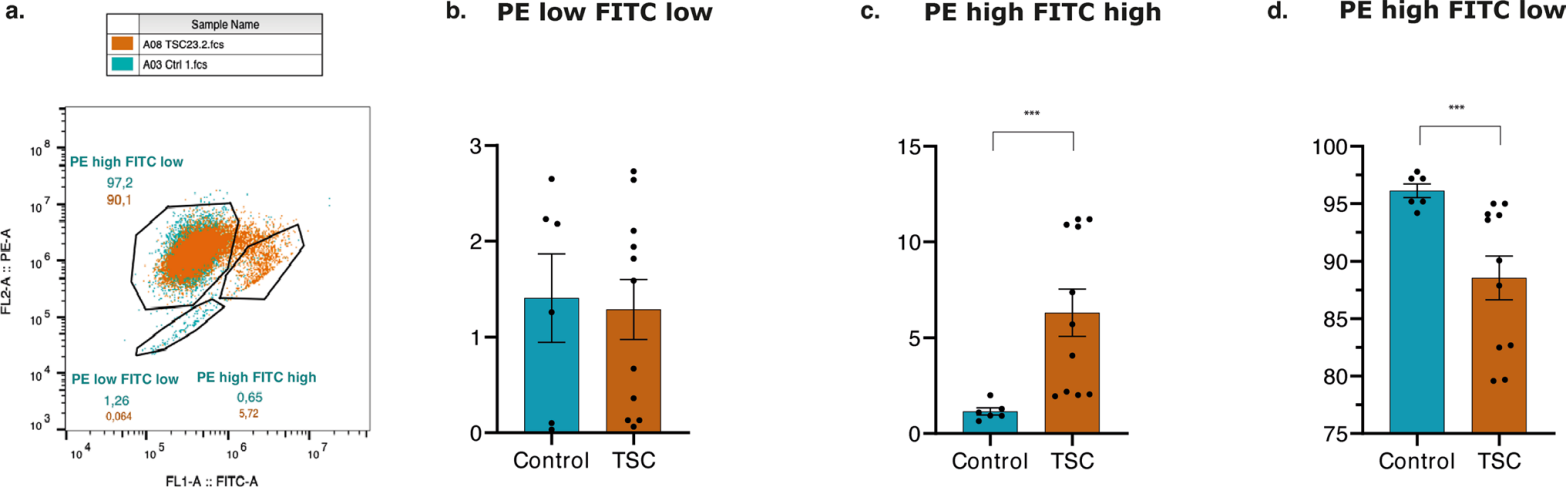
Flow cytometry of JC-1 staining. **a.** Representative figure of 10,000 separate events. Flow cytometry dot-plots of JC-1 fluorescence to measure ΔΨm in control astrocytes (blue dots) and TSC astrocytes (orange dots). Three subpopulations of cells were identified in both control and TSC astrocytes: ‘PE high FITC low’, ‘PE low FITC low’, and ‘PE high FITC high’ fluorescence. **b**–**d.** Barplots showing the percentage of control and TSC astrocytes with a positive JC-1 fluorescence intensity signal. No differences were identified between control and TSC astrocytes in ‘PE low FITC low’ fluorescence signal. TSC astrocytes showed a significant increase in PE high FITC high fluorescence intensity, while they showed a significant reduction in PE high FITC low fluorescence signal. Data are expressed as mean ± SEM; *p* value: *p value ≤ 0.05; **p value ≤ 0.01; ***p value ≤ 0.001; ****p value ≤ 0.0001. Mann–Whitney *U* test were used to evaluate significance

Ca^2+^ accumulation into mitochondria regulates its metabolism and the depolarization events of mitochondrial membrane potential. TSC astrocytes showed impairment in Ca^2+^ dynamics in mitochondria suggesting damages in mitochondrial membrane potential in TSC might occur. JC-1 staining was conducted to examine the ΔΨ_m_ in the experimental samples. Both control and TCS astrocytes exhibited 100% double positive staining for JC-1. The analysis of the experiments unveiled distinct subpopulations indicating different states of ΔΨ_m_ within each sample group based on the fluorescence intensity of the JC-1 dye: ‘PE low FITC low’, ‘PE high FITC high’ and ‘PE high FITC low’ (Fig. 7a). The ‘PE high FITC low’ population, constituting 97.2% and 90.1% of cells (control and TSC, respectively) were considered as cells with normally polarized mitochondria. Respectively, the ‘PE low FITC low’ and the ‘PE high FITC high’ population, which had a decreased PE/FITC (JC-1 aggregate/JC-1 monomer) ratio, were considered as cells with depolarized mitochondria. Notably, there was a significantly lower number of TSC cells in ‘PE high FITC low’ group compared to control cells (97.2%, *p* value: 0.0006) (Fig. 7d), while the number of TSC cells in the ‘PE high FITC high’ group was 8.8-fold higher compared to control cells (5.72%, *p* value: 0.0003) (Fig. 7b). No significant differences were found in ‘PE low FITC low’ group (Fig. 7c).

These results indicate that mitochondria in TSC astrocytes are significantly depolarized compared to the controls potentially contributing to mitochondrial dysfunction.

Collectively, these findings provide compelling evidence of an alteration in ΔΨ_m_, characterized by depolarization of the IMM, which suggests mitochondrial dysfunction in TSC astrocytes. The observed depolarization may indicate the presence of underlying cellular stress potentially damaging the components of the electron transport chain (ETC), impairing ATP synthesis or increased apoptosis leading to loss of mitochondria function. Therefore, we further explored the mitochondria respiratory capacity in TSC astrocytes.

### Reduced oxygen consumption rate and reserve respiratory capacity in TSC astrocytes

**Fig. 8:**
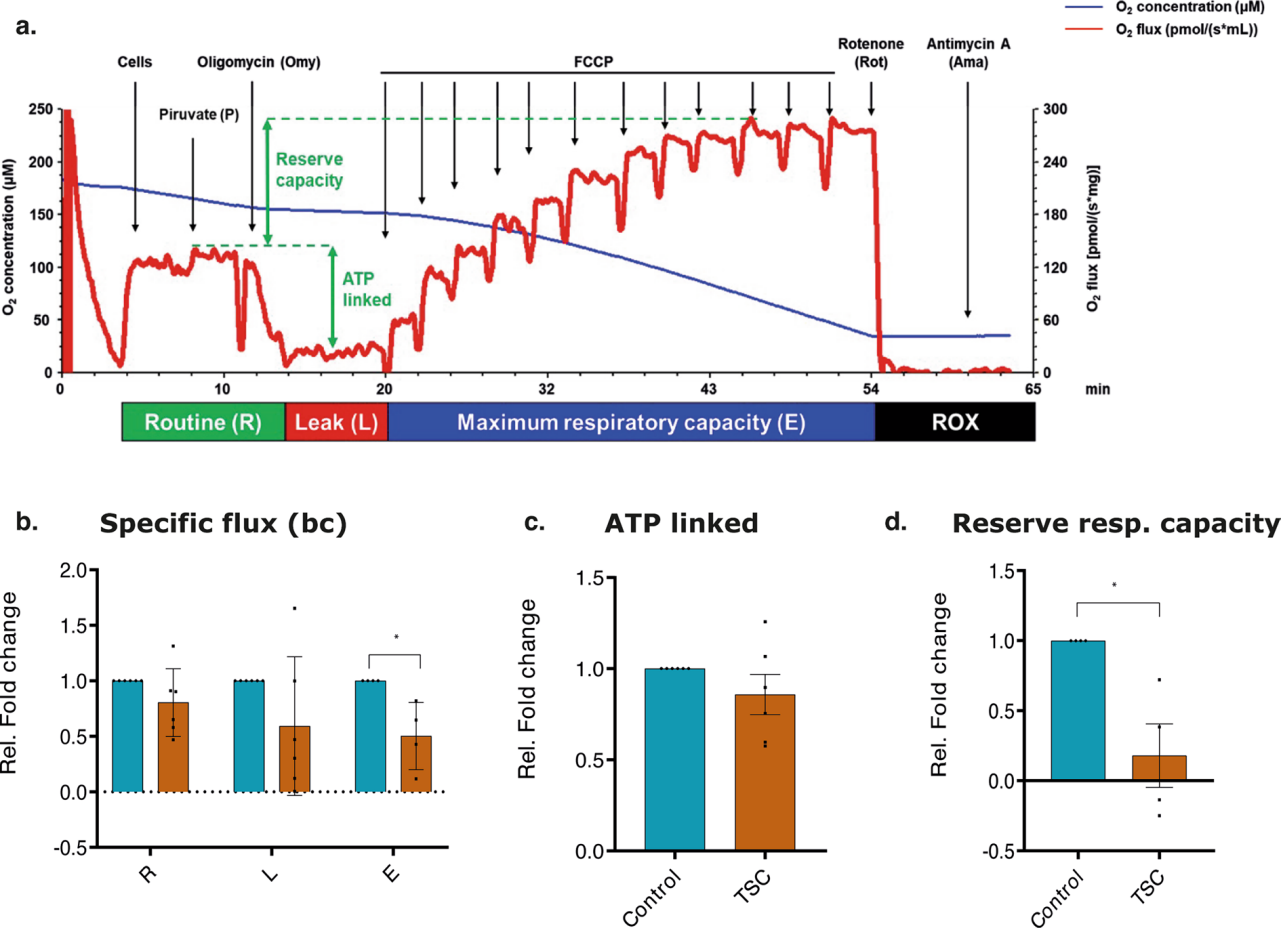
Oxygen consumption rate of control and TSC astrocytes normalized to protein content. **a.** Representative tracing of high-resolution respirometry to quantify intact cell respiration; blue line: oxygen concentration, red line: oxygen flux. **b.** Oxygen flux in the routine state (R); in the leakage state (L) after addition of oligomycin, an inhibitor of ATP synthetase; after the addition of FCCP, an uncoupler of oxidative phosphorylation to induce maximum respiratory capacity (E). All data are expressed as specific flux, i.e., oxygen consumption normalized to the sample protein content and after non-mitochondrial oxygen flux subtraction (ROX). **c.** Oxygen consumption linked to ATP production, i.e., oligomycin-sensitive respiration obtained by the subtraction of L from R. **d.** Reserve respiratory capacity obtained by the subtraction of R from E. Data are expressed as mean ± SEM of folds changes above the control; *p* value: *p value ≤ 0.05; **p value ≤ 0.01; ***p value ≤ 0.001; ****p value ≤ 0.0001. Mann–Whitney *U* test

The application of OROBOROS respirometry provided a comprehensive analysis of the respiration profiles of control and TSC astrocytes, enabling a deeper understanding of their mitochondrial function. This analysis performed in intact cells showed significant differences between control and TSC astrocytes in one of the three distinct phases of the mitochondrial respiration: ETC (*p* value: 0.016) (Fig. 8a and b). TSC astrocytes exhibited a reduction in basal oxygen consumption (R phase, Fig. 8b), indicating overall a lower energy demand compared to controls. The maximum capacity of the ETC was found to be significantly decreased (*p* value: 0.016) in TSC astrocytes when compared to control astrocytes (Fig. 8b). This reduction suggests a direct defect in the activity of the protein complexes involved in the ECT that led to an impaired ability of TSC astrocytes to generate ATP efficiently. While no difference was observed in ATP-linked respiration (Fig. 8c), TSC astrocytes showed a diminished reserve respiratory capacity (*p* value: 0.0286) compared to control astrocytes (Fig. 8d) further supporting the previous findings.

In conclusion, our findings reveal significant metabolic alterations in TSC astrocytes when compared to control astrocytes. The observed reductions in basal oxygen consumption, decreased ETC capacity, and diminished reserve respiratory capacity collectively suggest a compromised ability of TSC astrocytes to respond to heightened energy demands or cellular stress.

### EM-revealed alterations in mitochondrial ultrastructure in TSC astrocytes

**Fig. 9:**
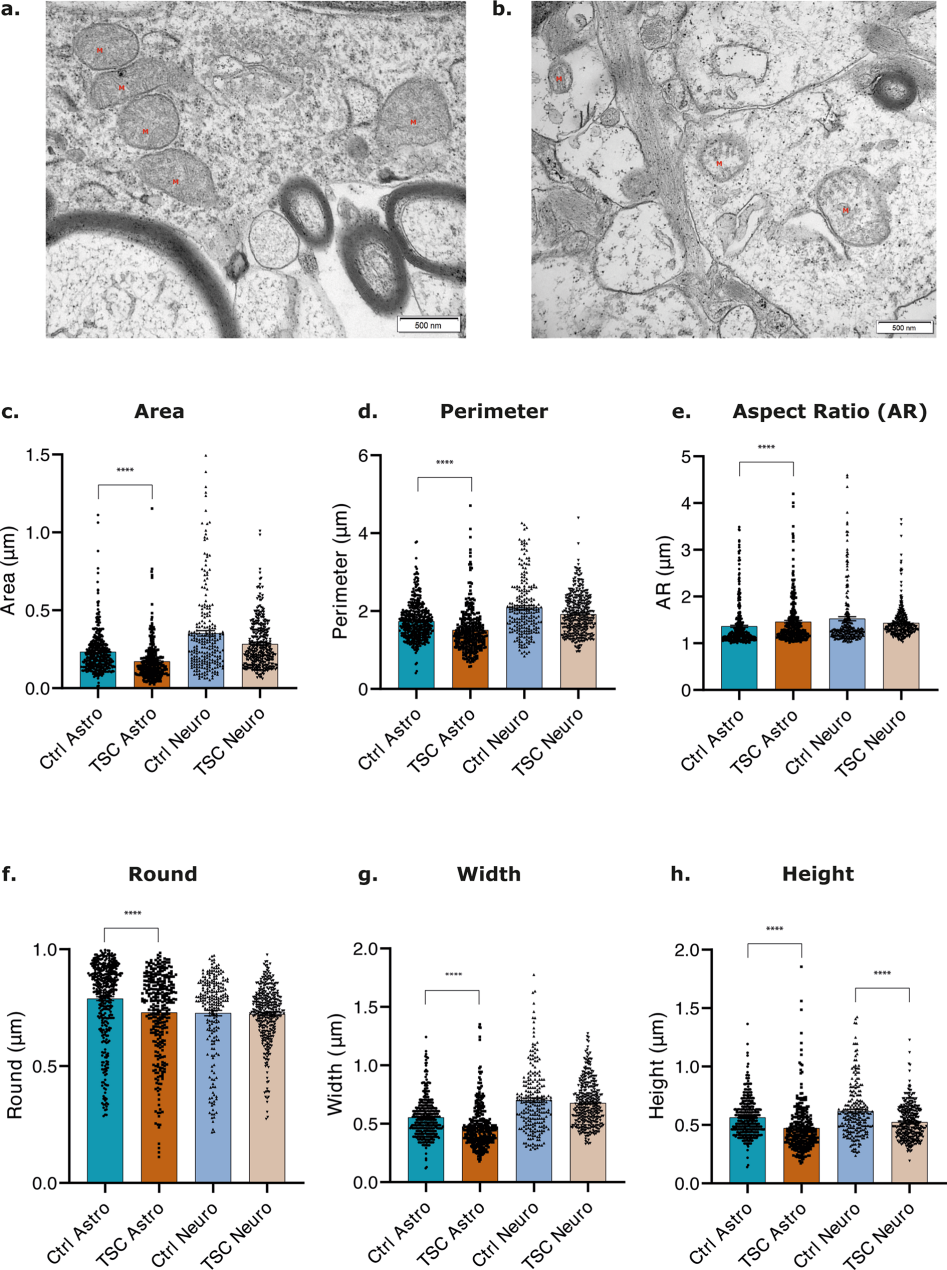
Electron microscopy analysis of mitochondria morphology in healthy-appearing astrocytes and neurons in TLE control tissue, astrocytes and dysmorphic neurons in TSC tissue. **a**, **b.** Electron microscopy of healthy-appearing astrocytes in TLE control tissue (a) and TSC tissue (b). Mitochondria were indicated by a red M. (scale bar: 500 nm**). c-h**. Multiple parameters were considered in the analysis to describe mitochondria morphology: area, perimeter, Aspect Ratio (AR), round, width, and height. TSC astrocytes showed significant reduction in area, perimeter, round, width and height compared to control astrocytes. **e.** A significant increase in the aspect ratio (AR) of mitochondria was identified in TSC astrocytes compared to control astrocytes. **h.** TSC neurons showed significant reduction in height compared to healthy-appearing TLE neurons. Data are expressed as mean ± SEM; Non-parametric Kruskal–Wallis H test was performed with correction for multiple comparisons. Significance is represented by *p* value: *p-value ≤ 0.05; **p value ≤ 0.01; ***p value ≤ 0.001; ****p value ≤ 0.0001

The observed alteration of mitochondrial calcium dynamics along with impairment in mitochondrial membrane depolarization and mitochondrial respiration might impact on their morphology and their ultrastructure in TSC astrocytes. EM was employed to examine the high-resolution morphology of mitochondria, enabling a detailed assessment of their structure, organization, and ultrastructural characteristics (Fig. 9a and b). Tissue samples obtained from individuals with TLE, and non-epileptogenic regions with histologically healthy-appearing neurons and astrocytes were selected as control specimens for comparative analysis with TSC tissues (Online Resource 1). Here, healthy-appearing astrocytes and neurons from individuals with TLE will be referred to as controls to evaluate mitochondrial ultrastructure. Several quantitative parameters of the mitochondria, including area, perimeter, aspect ratio (AR), roundness, circularity, Feret’s diameter, width, height, and integrated density, were measured to investigate potential alterations in mitochondrial ultrastructure in TSC astrocytes and neurons compared to the TLE control group. Mitochondria of TSC astrocytes showed significant alterations in their shape and elongation compared to control astrocytes. The observed significant reduction in perimeter and area (*p* value < 0.0001; *p* value < 0.0001, respectively), along with a significant increase in AR of mitochondria in TSC astrocytes (*p* value < 0.0001) (Fig. 9c, d and e), may indicate higher mitochondrial fragmentation associated with cellular dysfunction, increased fission/fusion, or changes in morphology due to cellular stress and/or changes in metabolic states [21]. Furthermore, TSC astrocytes exhibited a significant reduction in mitochondria circularity (*p* value < 0.0001), roundness (*p* value < 0.0001), and Feret’s diameter (*p* value < 0.0001*)* (Fig. 9f; Online Resource 16a and 16b) further corroborating the possibility of increased fragmentation, altered fission/fusion events, changes in the mitochondrial network, cristae structure, or remodeling of mitochondrial membranes. Lastly, the width (*p* value < 0.0001), the height (*p* value < 0.0001), and integrated density (*p* value < 0.0001) of mitochondria in TSC astrocytes were reduced (Fig. 9g and h; Online Resource 16c) suggesting a decrease in the size, impaired mitochondrial biogenesis, increased degradation, or dysfunction of the mitochondrial respiratory chain. These findings indicate potential alterations in the structural organization of mitochondria due to changes in mitochondrial dynamics, fusion, fission, or remodeling processes. A decrease in integrated density may also suggest mitochondrial depletion within the analyzed region.

No significant differences were found in mitochondria of TSC neurons compared to mitochondria of healthy-appearing TLE neurons in the majority of the parameters investigated except in their height (*p* value < 0.0001) (Fig. 9h).

## Discussion

This study aimed to explore the transcriptomic profiles of TSC brain-resected tissue and to investigate intracellular Ca^2+^ dynamics and signaling, along with their interrelation with cellular metabolism, in primary astrocytes isolated from TSC resected tissues. Further, we explored mitochondria ultrastructure via EM in TLE healthy-appearing astrocytes and TSC astrocytes. Through these investigations, we seek to shed light on the complex interplay between Ca^2+^ signaling, cellular metabolism, and structural changes in astrocytes, contributing to our understanding of the pathophysiological mechanisms underlying TSC and its associated conditions.

**Fig. 10:**
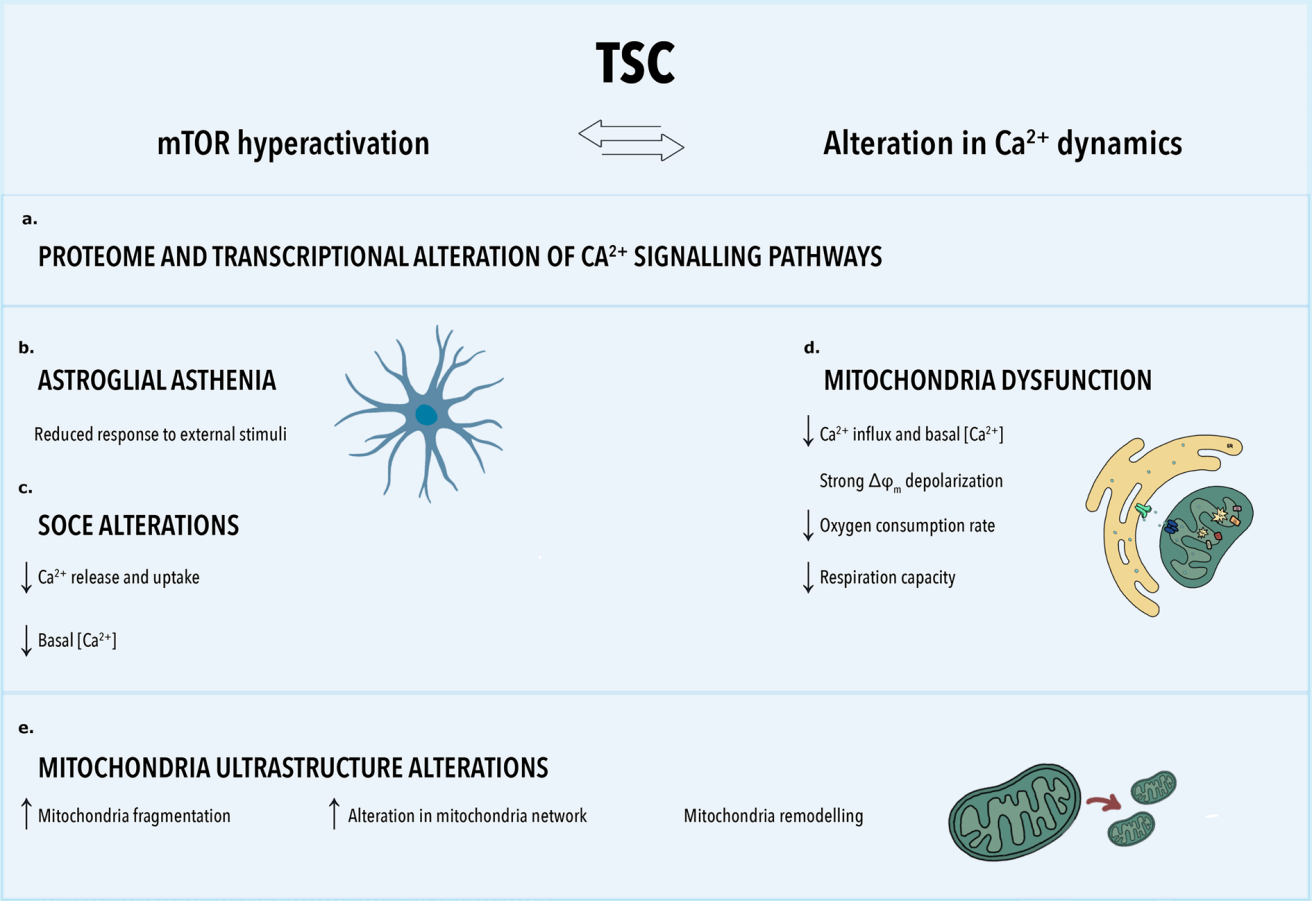
The mTOR hyperactivation in TSC patients results in strong alteration of calcium dynamics within the cells and multiple organelles. **a.** Proteomics analysis of TSC primary astrocytes culture and bulk RNASeq analysis and single-cell RNASeq of TSC patients derived material showed substantial alterations in pathways associated with cellular respiration, ER and mitochondria, Ca^2+^ regulation, and calcium-dependant neurotransmitter signaling, in addition to the well-established dysregulated immune response in TSC. **b.** TSC primary astrocytes culture showed a reduced response to external stimuli. **c.** TSC primary astrocytes culture showed dysfunction of store operated calcium entry (SOCE). A reduction in basal [Ca^2+^]_c_ levels was observed after SERCA blockade, and further Ca^2+^ stimulation suggest a dysregulation of release from the ER and SOCE in TSC astrocytes. **d.** TSC primary astrocytes culture showed mitochondrial dysfunction due to reduced calcium influx and efflux, strong depolarization of mitochondrial membrane potential (Δφ_m_), reduction in oxygen consumption rate and respiratory capacity **e.** Mitochondria ultrastructure alteration identified in astrocytes via electron microscopy analysis in TSC tissue. Astrocytes showed an alteration of relevant morphologic parameters which suggest increased mitochondria fragmentation, alteration in mitochondria network and structural remodeling. *TSC* tuberous sclerosis complex, *mTOR* mammalian target of rapamycin, *SOCE* store-operated calcium entry

The TSC transcriptional profile on a bulk RNA-Seq level revealed notable deficiencies in crucial molecular mechanisms encompassing Ca^2+^ intracellular influx and efflux processes, as well as compromised mobilization from storage organelles such as the ER and mitochondria (Fig. 10a). The differential expression of genes regulating Ca^2+^ uptake from the extracellular space, such as *TRPC* and *ORAI,* suggest a potential alteration in SOCE mechanism. Furthermore, genes responsible for ER Ca^2+^ uptake during SOCE were correlated with neuroinflammatory responses both in vitro and in vivo [17], and accumulation of unfolded or misfolded proteins in several neurodegenerative diseases potentially contributing to the impairment [15, 16, 77].

On a single-cell RNA-seq transcriptional level, we next identified eight genes associated with Ca^2+^ signaling that showed notable changes in astrocytes. The upregulation of genes such as MCUB and ITPR3 suggests an enhanced sensitivity to calcium signaling in astrocytes, potentially influencing their role in epileptogenesis [51, 97]. Conversely, the downregulation of NFATC4 points toward a complex interplay between astrocytic signaling and neuronal dysfunction [40, 65, 81].

Exploration of the proteomic signatures of TSC with IPA highlighted the activation of immune response and the positive regulation of cytokine production further confirmed by the pathway analysis of the transcriptome. Furthermore, the IPA revealed impairment in the Slit/Robo pathways and collagen biosynthesis pathways associated with epilepsy, glioma and ASD [3, 31, 46]. Moreover, IPA analysis of the TSC primary cultures proteome and the pathway enrichment analysis of the transcriptome predicted significant modulation of pathways involved in the immune response, ECM and regulation of mitotic cell cycle supporting the immature phenotype as well the strong neuroinflammation and alteration of brain ECM in the epileptogenic network of TSC[7, 62].

In line with these transcriptomic data, in this study, we describe a reduction of Ca^2+^ uptake from the extracellular space as well as impaired Ca^2+^ efflux from the ER further indicating an impairment of SOCE and a decrease in [Ca^2+^]_ER_ [96] (Fig. 10c). Impairment of Ca^2+^ dynamics have been linked to apoptosis, autophagy and alteration of cell cycle [13, 67, 83, 107]. Moreover, the reduced [Ca^2+^]_c_ and impaired SOCE disrupt the mitotic chromosome organization affecting G1/S cell cycle transition and consequently giving rise to an immature state and increased autophagy [9, 20, 24, 78].

Across all in vitro functional experiments, TSC astrocytes displayed a reduced ability to respond to stimuli (Fig. 10b). This astroglial asthenia, along with atrophy and loss of homeostatic and neuroprotective capabilities, has been well described in various neurodegenerative disorders such as AD, PD, and HD and neuropsychiatric disorders, such as schizophrenia and major depressive disorders [103–105]. Furthermore, it has been recently reported that TSC astrocytes show reduced maturity as well as the inability to clear excess glutamate and phagocytosis, chronic activation of anti‐oxidant pathways and iron accumulation [62, 117, 118]. Therefore, further characterization of its relevance to the epileptogenesis of mTORopathies could be of interest.

The genes regulating Ca^2+^ release from the ER, such as *IP*_*3*_*R* and *RYR*, were differentially expressed further confirming the altered cytoplasmic Ca^2+^ signaling and indicating impaired Ca^2+^ influx into the mitochondrial matrix. Altered *IP*_*3*_*R* expression has implications in conditions like secondary axonal degeneration and neuronal cell death [49, 54, 74], whilst RyR dysregulation is associated with neuronal vulnerability, synaptic dysfunction, and neurodegenerative diseases [1, 30, 32, 68, 115]. When mTOR is hyperactivated, the reduction in Ca^2+^ release via IP_3_R3 mediated by Akt is inhibited, suggesting increased apoptosis and immaturity, reduced cells growth and cellular energy metabolism [19, 48].

The decreased [Ca^2+^]_c_, along with a diminished capacity for Ca^2+^ reuptake via SOCE and consequently reduced [Ca^2+^]_ER_ storage could potentially impact the uptake and storage of Ca^2+^ within the mitochondria (Fig. 9d). This is attributed to the finely tuned interplay between the ER and mitochondria in the context of intracellular Ca^2+^ signaling. Our TSC transcriptome data showed the upregulation of *MCUB*, the negative regulator of mitochondrial Ca^2+^ uptake localized on the IMM. In addition, the data indicated a potential increase in Ca^2+^ efflux from the mitochondria due to the upregulation of *SLC8B1* encoding for NCLX, localized on the IMM. We further explored mitochondrial Ca^2+^ dynamics. The TSC astrocytes showed a reduction in basal [Ca^2+^]_m_ and reduced responsiveness to stimuli (ATP and ionomycin) (Fig. 10d). By employing ionomycin stimulation, we evaluated the functionality of MCU independently of the ER Ca^2+^ release capacity and the efficiency of the ER-mitochondria Ca^2+^ transfer as it is the only protein complex responsible for calcium transfer into the mitochondria after ionomycin stimulation [56]. TSC astrocytes did not exhibit a response to the stimulation displaying a further reduction in Ca^2+^ influx suggesting the upregulation of MCUB, expressed on the IMM, might be compromising their ability to uptake Ca^2+^ from the cytosol, possibly compromising OXPHOS and stimulating autophagy [18] (Fig. 10d).

Reduced-Ca^2+^ influx can diminish stimulation of OXPHOS, curbing ATP production. This decrease in energy production and potential impact on Ca^2+^-dependent enzyme reactions can intensify mitochondrial membrane depolarization. Furthermore, altered Ca^2+^ dynamics not only affect Δψ_m_ but they also affect the rate of Ca^2+^ efflux from the mitochondria, promoting superoxide generation and osmotic swelling of the mitochondrial matrix [14, 57]. Consistent with this, TSC astrocytes showed a significant depolarization of Δψ_m_ and significant reduction of ECT maximum capacity and reserve respiratory capacity potentially due to an accumulation of a proton gradient in mitochondrial intermembrane (Fig. 10d). These strong metabolic alterations and reduced basal oxygen consumption confirmed the compromised ability of TSC astrocytes to functionally respond to heightened energy demands or to cellular stress as well as to external stimuli (Fig. 10d). Lastly, these metabolic alterations are consistent with the enrichment in pro-oxidant and anti-oxidant pathways in our pathway analysis on TSC tissue and with other studies confirming upregulation of oxidative stress markers in various cell types, including dysmorphic neurons and glia, in TSC [117, 118].

Mitochondrial functional impairment is frequently associated with aberrant morphology [79] and, therefore, we explored mitochondria ultrastructure via EM in TLE healthy-appearing astrocytes and TSC astrocytes. Our study describes for the first time that TSC astrocytes exhibited significant reduction across all the EM parameters, indicating potential alterations in the structural organization of mitochondria due to changes in mitochondrial dynamics, fusion, fission, or remodeling processes. We showed a decrease in integrated density suggesting mitochondrial depletion within the analyzed region possibly due to increased mitophagy or reduced mitochondrial biogenesis (Fig. 10e). While these data support the link between functional and morphologic alterations of mitochondria in TSC astrocytes, TSC neurons did not exhibit altered mitochondria morphology compared to TLE healthy-appearing neurons. Furthermore, impairment of mitochondrial morphology and functionality have been reported to contribute to stress-induced inflammation and cellular senescence, involving heightened superoxide generation, elongated mitochondria, and impaired OXPHOS, leading to cell cycle arrest [34, 35, 99, 112]. However, further exploration on whether mitochondrial morphology transitions directly control cellular signals or vice versa is still required.

TSC comorbidities include neuropsychiatric problems with behavioral abnormalities and ASD is one of the most prominent conditions [27, 52, 95]. Several meta-analysis and biomarker studies revealed abnormalities in mitochondrial function in individuals with ASD appear to be much higher than the prevalence of those diagnosed with classical mitochondrial disease [88, 90, 113]. A relatively substantial proportion of children with ASD exhibit mitochondrial dysfunction, which may arise from genetic alterations, metabolic irregularities, or exposure to environmental toxins. Moreover, it has been proposed that neurodevelopmental regression could potentially serve as a hallmark of mitochondrial dysfunction in ASD [39, 94].

In conclusion, our study not only elucidated the distinct patterns of gene expression associated with Ca^2+^-signaling pathways within a cohort of TSC patients but also uncovered significant impairment in mitochondrial functionality in TSC primary astrocytes. In addition, understanding the functional consequences of the observed reduced responsiveness of TSC astrocytes to certain stimuli unveils a significant avenue for future exploration. Furthermore, the potential link between this reduced responsiveness and the astrocytes reactivity, which is commonly associated with TSC, warrants in-depth investigation to elucidate TSC pathophysiology. We provide the initial evidence pointing toward structural abnormalities in mitochondria within astrocytes derived from tissues of TSC patients. Investigating the alterations in Ca^2+^ dynamics within more complex biologic systems like cocultures, organoids or tissue sections would strengthen our study and provide valuable insights into the molecular mechanisms underlying astrocyte dysfunction in TSC in the presence of other nervous system components. Further exploration of the complex interplay between Ca^2+^ signaling, mitochondria dynamics, apoptosis, and mTOR hyperactivation will be required to improve our understanding on the pathophysiology of TSC and associated neuropsychiatric disorders and pave the way to potential targeted therapeutic interventions. In addition, the exploration of strategies aimed at enhancing mitochondrial function holds promise for achieving disease modification in TSC, offering further potential avenues for therapeutic development.

### Supplementary Information

Below is the link to the electronic supplementary material.

Supplementary file1 (PDF 1375 KB)

Supplementary file2 (TIF 30433 KB)

Supplementary file3 (TIF 8179 KB)

Supplementary file4 (TIF 9692 KB)

Supplementary file5 (TIF 12495 KB)

## Data Availability

The datasets generated and analyzed during the current study are available on the European Genome-phenome Archive (EGA) data repository. The EGA can be found at ega-archive.org.
