# Supplementary material for: Astroglial calcium signaling and homeostasis in tuberous sclerosis complex
Source: Acta Neuropathol. 2024 Feb 28;147(1):48. doi: 10.1007/s00401-024-02711-3 (PMC10901927; doi:10.1007/s00401-024-02711-3)
Supplement: Supplementary file 1 — Supplementary file1 (PDF 1375 KB) [file 401_2024_2711_MOESM1_ESM.pdf]

**Online resources (Acta Neuropathologica)**

# Astroglial calcium signalling and homeostasis in Tuberous Sclerosis Complex

Alessia Romagnolo[1]*, Giulia Dematteis[2]*, Mirte Scheper[1], Mark J. Luinenburg[1], Angelika Mühlebner[1,3], Wim Van Hecke[,3], Marcello Manfredi[4], Simone Reano[4], Nicoletta Filigheddu[5], Valeria Bortolotto[2], Laura Tapella[2], Jasper J. Anink[1], Liesbeth François[6], Stefanie Dedeurwaerdere[6], James D. Mills[1,7,8], Armando A Genazzani[2], Dmitry Lim[2]**, Eleonora Aronica[1,9]**

* Shared first authorship, ** Shared last authorship

Corresponding author:
Alessia Romagnolo
a.romagnolo@amsterdamumc.nl
+31 627598894

[1] Department of (Neuro)Pathology, Amsterdam UMC, University of Amsterdam, Amsterdam Neuroscience, Amsterdam, The Netherlands.
[2] Department of Pharmaceutical Sciences, Università del Piemonte Orientale "Amedeo Avogadro", Novara, Italy.
[3] Department of Pathology, University Medical Center Utrecht, Utrecht, The Netherlands.
[4] Center on Autoimmune and Allergic Diseases (CAAD), UPO; Novara
[5] Department of Translational Medicine, UPO, Novara
[6] Neurosciences Therapeutic Area, UCB Pharma, Braine-l'Alleud, Belgium.
[7] Department of Clinical and Experimental Epilepsy, UCL, London, UK.
[8] Chalfont Centre for Epilepsy, Chalfont St Peter, UK.
[9] Stichting Epilepsie Instellingen Nederland (SEIN), Heemstede, The Netherlands.

**Online Resource 1 Clinical informations on TSC (n=21) and control cortex (n=15) cohort.**

| | Diagnose | Gender | Seizure Onset (years) | Age at the time of operation (years) | Duration (years) | Area of Resection | Mutation |
|---|---|---|---|---|---|---|---|
| 1[a] | TSC | f | 0 | 13 | 13 | F | TSC2 |
| 2[a] | TSC | m | 0 | 2 | 2 | F | TSC2 |
| 3[a] | TSC | f | 20 | 21 | 1 | T | TSC1 |
| 4[a] | TSC | m | 6 | 14 | 8 | F | TSC1 |
| 5[a] | TSC | m | 0 | 4 | 4 | T | TSC1 |
| 6[a] | TSC | f | 0 | 2 | 2 | T | TSC1 |
| 7[a] | TSC | m | 0 | 2 | 2 | F | TSC2 |
| 8[a] | TSC | m | 0 | 2 | 2 | F | TSC2 |
| 9[a] | TSC | m | 0 | 1 | 1 | F | TSC2 |
| 10[a] | TSC | m | 0 | 0 | 0 | F | TSC2 |
| 11[a] | TSC | m | 0 | 5 | 5 | F | TSC2 |
| 12[a] | TSC | m | 0 | 0 | 0 | F | TSC2 |
| 13[a] | TSC | m | 2 | 3 | 1 | F | TSC1 |
| 14[a] | TSC | f | 0 | 8 | 8 | F | TSC2 |
| 15[a] | TSC | f | 8 | 9 | 1 | F | TSC2 |
| 16[a] | TSC | v | 0 | 0 | 0 | F | TSC2 |
| 17[a] | TSC | f | 26 | 30 | 4 | T | TSC1 |
| 18[a] | TSC | f | 0 | 0 | 0 | T | TSC2 |
| 19[a] | TSC | f | 0 | 17 | 17 | P | TSC2 |
| 20[a] | TSC | f | 5 | 11 | 6 | T | TSC2 |
| 21[a] | TSC | m | 0 | 4 | 4 | F | TSC2 |
| 22[a] | Control | f | NA | 0 | NA | C | NA |
| 23[a] | Control | m | NA | 10 | NA | C | NA |
| 24[a] | Control | f | NA | 0 | NA | C | NA |
| 25[a] | Control | f | NA | 2 | NA | C | NA |
| 26[a] | Control | m | NA | 15 | NA | C | NA |
| 27[a] | Control | m | NA | 13 | NA | C | NA |
| 28[a] | Control | f | NA | 0 | NA | C | NA |
| 29[a] | Control | m | NA | 10 | NA | C | NA |
| 30[a] | Control | f | NA | 17 | NA | C | NA |
| 31[a] | Control | f | NA | 17 | NA | C | NA |
| 32[a] | Control | f | NA | 61 | NA | C | NA |
| 33[a] | Control | f | NA | 44 | NA | C | NA |
| 34[a] | Control | m | NA | 60 | NA | C | NA |
| 35[a] | Control | f | NA | 39 | NA | C | NA |
| 36[a] | Control | m | NA | 49 | NA | C | NA |
| 37[b] | Control | m | NA | 12 | NA | C | NA |
| 38[b] | Control | f | NA | 16 | NA | C | NA |

| | | | | | | | |
|---|---|---|---|---|---|---|---|
| 39[b] | Control | f | NA | 5 | NA | C | NA |
| 40[b] | TSC | m | 0 | 2 | 2 | F | TSC2 |
| 41[b] | TSC | m | 0 | 5 | 5 | F | TSC2 |
| 42[b] | TSC | f | 0 | 8 | 8 | F | TSC2 |
| 43[b] | TSC | f | 0 | 0 | 0 | T | TSC2 |
| 44[b] | TSC | f | 0 | 17 | 17 | P | TSC2 |
| 45[c] | TLE-HS | m | 2 | 34 | 32 | T | HS2 |
| 46[c] | TSC | m | 8 | 13 | 5 | P | TSC2 |

a=patients involved in the BulkRNASeq; b= patients involved in the single-cell RNASeq; c=patients subjected to EM; m=male; f=female; TSC=Tuberous Sclerosis Complex; TLE-HS=Temporal Lobe Epilepsy with Hippocampal Sclerosis; HS2=Hippocampal Sclerosis type 2; F=Frontal; T=Temporal; P=Parietal; C=Cortex.

**Online Resource 2** Marker genes for scRNA-seq cell type clustering annotation

| **Cell type** | **Marker genes** |
|---|---|
| Glutamatergic neurons | SLC17A7, SATB2, SLC17A6, NMDAR1 |
| GABAergic neurons | GAD1, GAD2 |
| Astrocytes | GFAP, ALDH1L1, AQP4, GLT1, S100B |
| Microglia | PTPRC, ITGAM, P2RY12, TMEM119 |
| Oligodendrocytes | OLIG1, GLDN11, MOG, MBP |
| OPCs | PDGFRA, CSPG4 |
| Endothelial cells | CLDN5, FOXJ1, PDGFRB |

**Online Resource 3 Bulk RNASeq analysis of control cortex and TSC cohort. a-b.** Principal components analysis (PCA) of the whole transcriptome profile was performed for each sample in control and TSC cohort. Each point represented a different sample. PCA did not identify pathology or surgical area of resection as discriminating features in the cohort. X-axis: the first principal component (PC1); y-axis: the second principal component (PC2). **c.** Volcano plot showing the differentially expressed genes (DEGs) (p.adj < 0.05) between control cortex (n=15) and TSC (n=21) tissue. A total of 2766 mRNAs were found to be upregulated and 1780 downregulated in TSC compared to control tissue. **d.** Supervised clustering heatmap visualizing the expression of the relevant $Ca^{2+}$-related genes in control tissues and TSC cortical tubers. Adjusted p-value: * p.adj ≤ 0.05; ** p.adj ≤ 0.01; *** p.adj ≤ 0.001; **** p.adj ≤ 0.0001).

**Online Resource 4 Significantly enriched GO Biological processes (BP) pathways of upregulated genes in TSC patients' tissue**

| **ID** | **Description** | **pvalue** | **p.adjust** |
|---|---|---|---|
| GO:0042119 | neutrophil activation | 7.95356377776396e-25 | 4.71089582556959e-21 |
| GO:0002283 | neutrophil activation involved in immune response | 2.37831563027281e-24 | 7.04338173905292e-21 |

| | | | |
|---|---|---|---|
| GO:0043312 | neutrophil degranulation | 4.20064916883927e-24 | 8.29348167567833e-21 |
| GO:0002446 | neutrophil mediated immunity | 6.77615207245481e-24 | 1.00337871812875e-20 |
| GO:0070661 | leukocyte proliferation | 3.25156159283991e-20 | 3.85179986287816e-17 |
| GO:0007159 | leukocyte cell-cell adhesion | 7.89953519280749e-20 | 7.79815782449979e-17 |
| GO:0042110 | T cell activation | 1.32272139425595e-19 | 1.11921125973972e-16 |
| GO:0070663 | regulation of leukocyte proliferation | 2.94480356273144e-19 | 2.18025893775729e-16 |
| GO:0032943 | mononuclear cell proliferation | 4.53695297324811e-19 | 2.98581916228317e-16 |
| GO:0032944 | regulation of mononuclear cell proliferation | 5.92466859301897e-19 | 3.50918120764514e-16 |
| GO:0046651 | lymphocyte proliferation | 8.12430847894113e-19 | 4.37457082916076e-16 |
| GO:0050670 | regulation of lymphocyte proliferation | 1.41734527641908e-18 | 6.99578006019183e-16 |
| GO:1903039 | positive regulation of leukocyte cell-cell adhesion | 2.95661860842203e-18 | 1.34708092443721e-15 |
| GO:0042098 | T cell proliferation | 2.1230050032022e-17 | 8.47775493492529e-15 |
| GO:1903037 | regulation of leukocyte cell-cell adhesion | 2.14699179510179e-17 | 8.47775493492529e-15 |
| GO:0001819 | positive regulation of cytokine production | 2.49455561279002e-17 | 9.23453305909707e-15 |
| GO:0071346 | cellular response to interferon-gamma | 3.76029581234342e-17 | 1.31013129979471e-14 |
| GO:0050867 | positive regulation of cell activation | 6.30257509507081e-17 | 2.07389734933913e-14 |
| GO:0045785 | positive regulation of cell adhesion | 7.38573742499244e-17 | 2.3024064614858e-14 |
| GO:0034341 | response to interferon-gamma | 1.58528598169896e-16 | 4.69482443480148e-14 |
| GO:1902105 | regulation of leukocyte differentiation | 2.34115732771376e-16 | 6.60317850097553e-14 |
| GO:0050863 | regulation of T cell activation | 3.90372410427708e-16 | 1.05098899407423e-13 |
| GO:0050870 | positive regulation of T cell activation | 5.4759278738299e-16 | 1.4101704694215e-13 |
| GO:0002696 | positive regulation of leukocyte activation | 1.8407663654474e-15 | 4.54285799272707e-13 |
| GO:0022409 | positive regulation of cell-cell adhesion | 4.68815910257158e-15 | 1.11071865458126e-12 |
| GO:0007249 | I-kappaB kinase/NF-kappaB signaling | 4.99600148143403e-15 | 1.1381275682513e-12 |
| GO:1902107 | positive regulation of leukocyte differentiation | 5.68865841384996e-15 | 1.20335442090119e-12 |
| GO:1903708 | positive regulation of hemopoiesis | 5.68865841384996e-15 | 1.20335442090119e-12 |
| GO:0032946 | positive regulation of mononuclear cell proliferation | 9.0105505316081e-15 | 1.8403272689212e-12 |
| GO:0060333 | interferon-gamma-mediated signaling pathway | 1.04996812035856e-14 | 2.07298705896124e-12 |
| GO:0042129 | regulation of T cell proliferation | 1.80955170079618e-14 | 3.45741120123089e-12 |

| | | | |
|---|---|---|---|
| GO:0022407 | regulation of cell-cell adhesion | 3.05665329883226e-14 | 5.55435852501408e-12 |
| GO:0050671 | positive regulation of lymphocyte proliferation | 3.09461136798015e-14 | 5.55435852501408e-12 |
| GO:0070665 | positive regulation of leukocyte proliferation | 8.16664884451953e-14 | 1.42267826782615e-11 |
| GO:0002697 | regulation of immune effector process | 1.14314395917265e-13 | 1.93452619147989e-11 |
| GO:1903131 | mononuclear cell differentiation | 1.89502506671047e-13 | 3.11784263059059e-11 |
| GO:0042102 | positive regulation of T cell proliferation | 2.61772041932091e-13 | 4.19047514692913e-11 |
| GO:0043122 | regulation of I-kappaB kinase/NF-kappaB signaling | 8.42875177669645e-13 | 1.31377623087824e-10 |
| GO:0002221 | pattern recognition receptor signaling pathway | 1.82666003055339e-12 | 2.77418137460711e-10 |
| GO:0002683 | negative regulation of immune system process | 2.5789777704367e-12 | 3.75288265797246e-10 |
| GO:0043123 | positive regulation of I-kappaB kinase/NF-kappaB signaling | 2.5978083568609e-12 | 3.75288265797246e-10 |
| GO:0002224 | toll-like receptor signaling pathway | 4.00146314394573e-12 | 5.64301576228347e-10 |
| GO:0051251 | positive regulation of lymphocyte activation | 4.5270918792736e-12 | 6.23580586068314e-10 |
| GO:0002819 | regulation of adaptive immune response | 7.16126483138208e-12 | 9.64003899915365e-10 |
| GO:1903557 | positive regulation of tumor necrosis factor superfamily cytokine production | 7.91486840417187e-12 | 1.04177256795356e-09 |
| GO:0071706 | tumor necrosis factor superfamily cytokine production | 1.27584701395252e-11 | 1.64279170948712e-09 |
| GO:0032760 | positive regulation of tumor necrosis factor production | 1.40793038475822e-11 | 1.74151166662743e-09 |
| GO:0046634 | regulation of alpha-beta T cell activation | 1.41132128985508e-11 | 1.74151166662743e-09 |
| GO:0030098 | lymphocyte differentiation | 1.60976335945233e-11 | 1.94584252612982e-09 |
| GO:0002237 | response to molecule of bacterial origin | 1.77845113859007e-11 | 2.1067532187738e-09 |
| GO:1903555 | regulation of tumor necrosis factor superfamily cytokine production | 2.27200413988865e-11 | 2.63864323932558e-09 |
| GO:0042063 | gliogenesis | 4.21853222933616e-11 | 4.80507046045348e-09 |
| GO:0032640 | tumor necrosis factor production | 5.12401772093999e-11 | 5.72633150209954e-09 |
| GO:0002573 | myeloid leukocyte differentiation | 9.27442259849265e-11 | 1.01726676020133e-08 |
| GO:0046631 | alpha-beta T cell activation | 1.11145092512274e-10 | 1.19453157123955e-08 |
| GO:0002685 | regulation of leukocyte migration | 1.13239438864348e-10 | 1.19453157123955e-08 |
| GO:0032680 | regulation of tumor necrosis factor production | 1.14955764917533e-10 | 1.19453157123955e-08 |
| GO:0002822 | regulation of adaptive immune response based on somatic recombination of immune receptors built from immunoglobulin superfamily domains | 1.27935442016217e-10 | 1.30414123780628e-08 |
| GO:0046635 | positive regulation of alpha-beta T cell activation | 1.31404899503929e-10 | 1.30414123780628e-08 |
| GO:0050866 | negative regulation of cell activation | 1.32109529408031e-10 | 1.30414123780628e-08 |

| | | | |
|---|---|---|---|
| GO:0002699 | positive regulation of immune effector process | 1.77712378945827e-10 | 1.7255580663871e-08 |
| GO:0032635 | interleukin-6 production | 1.81848109031955e-10 | 1.73723604805849e-08 |
| GO:1903706 | regulation of hemopoiesis | 1.96961587053037e-10 | 1.85175155573831e-08 |
| GO:0032675 | regulation of interleukin-6 production | 2.56495425230062e-10 | 2.37378500568384e-08 |
| GO:0045619 | regulation of lymphocyte differentiation | 2.72820753140144e-10 | 2.48602664746011e-08 |
| GO:0030217 | T cell differentiation | 3.10671936314492e-10 | 2.77649114342353e-08 |
| GO:0019882 | antigen processing and presentation | 3.14072103004181e-10 | 2.77649114342353e-08 |
| GO:0002695 | negative regulation of leukocyte activation | 3.6068533660015e-10 | 3.14167536570984e-08 |
| GO:0032609 | interferon-gamma production | 4.04728283232637e-10 | 3.47421104577813e-08 |
| GO:0042116 | macrophage activation | 4.13971944046911e-10 | 3.50279403512836e-08 |
| GO:0046633 | alpha-beta T cell proliferation | 6.4495937181605e-10 | 5.38041459051614e-08 |
| GO:0032496 | response to lipopolysaccharide | 6.74527188493481e-10 | 5.54892296867623e-08 |
| GO:0002703 | regulation of leukocyte mediated immunity | 7.51450581850734e-10 | 6.09704355657794e-08 |
| GO:0032755 | positive regulation of interleukin-6 production | 1.26978522315222e-09 | 1.01634295631495e-07 |
| GO:0045621 | positive regulation of lymphocyte differentiation | 1.33950515679899e-09 | 1.05785187249606e-07 |
| GO:0032649 | regulation of interferon-gamma production | 1.72952876525788e-09 | 1.34789458902926e-07 |
| GO:0002755 | MyD88-dependent toll-like receptor signaling pathway | 1.77100666109683e-09 | 1.36229512385409e-07 |
| GO:0001774 | microglial cell activation | 2.14138779746229e-09 | 1.62608204158579e-07 |
| GO:0045582 | positive regulation of T cell differentiation | 2.38294351010367e-09 | 1.78660435573975e-07 |
| GO:0045580 | regulation of T cell differentiation | 2.51109668434932e-09 | 1.85915320767513e-07 |
| GO:0051250 | negative regulation of lymphocyte activation | 3.41166243920669e-09 | 2.49472550955817e-07 |
| GO:0046640 | regulation of alpha-beta T cell proliferation | 8.78248735450346e-09 | 6.3437405610639e-07 |
| GO:0032663 | regulation of interleukin-2 production | 9.28300190109869e-09 | 6.61899246784713e-07 |
| GO:0060326 | cell chemotaxis | 9.38705668241024e-09 | 6.61899246784713e-07 |
| GO:0048002 | antigen processing and presentation of peptide antigen | 1.10525882773665e-08 | 7.7017035725696e-07 |
| GO:0007162 | negative regulation of cell adhesion | 1.2375898061523e-08 | 8.52354002539547e-07 |
| GO:0098581 | detection of external biotic stimulus | 1.35366291901002e-08 | 9.21579938999579e-07 |
| GO:0050764 | regulation of phagocytosis | 1.37765632772185e-08 | 9.27256639670059e-07 |
| GO:0032729 | positive regulation of interferon-gamma production | 1.56789317025717e-08 | 1.04344171319475e-06 |

| | | | |
|---|---|---|---|
| GO:0071216 | cellular response to biotic stimulus | 1.60234924878366e-08 | 1.05452384450507e-06 |
| GO:0050729 | positive regulation of inflammatory response | 1.95337891396726e-08 | 1.26290269525714e-06 |
| GO:0032623 | interleukin-2 production | 1.96162498672391e-08 | 1.26290269525714e-06 |
| GO:0010001 | glial cell differentiation | 2.13638657258311e-08 | 1.36062555585051e-06 |
| GO:0030198 | extracellular matrix organization | 2.17917240083264e-08 | 1.37311043937572e-06 |
| GO:0043062 | extracellular structure organization | 2.44336254159008e-08 | 1.52337224566716e-06 |
| GO:0002460 | adaptive immune response based on somatic recombination of immune receptors built from immunoglobulin superfamily domains | 2.52086618324238e-08 | 1.55532191701506e-06 |
| GO:0046394 | carboxylic acid biosynthetic process | 2.62295750372633e-08 | 1.60162652521351e-06 |
| GO:0045229 | external encapsulating structure organization | 3.06597579841462e-08 | 1.82715839576169e-06 |
| GO:0032677 | regulation of interleukin-8 production | 3.09429164806969e-08 | 1.82715839576169e-06 |
| GO:0016053 | organic acid biosynthetic process | 3.09590906141486e-08 | 1.82715839576169e-06 |
| GO:0050766 | positive regulation of phagocytosis | 3.11570146837635e-08 | 1.82715839576169e-06 |
| GO:0002763 | positive regulation of myeloid leukocyte differentiation | 3.50309180012952e-08 | 2.01444783807448e-06 |
| GO:0031663 | lipopolysaccharide-mediated signaling pathway | 3.50309180012952e-08 | 2.01444783807448e-06 |
| GO:0032637 | interleukin-8 production | 3.72663064139475e-08 | 2.12238781624818e-06 |
| GO:0002698 | negative regulation of immune effector process | 3.9158285084611e-08 | 2.20890021482049e-06 |
| GO:0002821 | positive regulation of adaptive immune response | 4.77975330845704e-08 | 2.67079989113123e-06 |
| GO:0007272 | ensheathment of neurons | 4.87996243688093e-08 | 2.67629791793016e-06 |
| GO:0008366 | axon ensheathment | 4.87996243688093e-08 | 2.67629791793016e-06 |
| GO:0071219 | cellular response to molecule of bacterial origin | 4.96342238479657e-08 | 2.69709640230735e-06 |
| GO:0002824 | positive regulation of adaptive immune response based on somatic recombination of immune receptors built from immunoglobulin superfamily domains | 5.1866652352571e-08 | 2.79278347167526e-06 |
| GO:0009595 | detection of biotic stimulus | 7.17350764915123e-08 | 3.82780953206511e-06 |
| GO:0002761 | regulation of myeloid leukocyte differentiation | 9.79233463625319e-08 | 5.1785712545114e-06 |
| GO:0042552 | myelination | 1.06670110338381e-07 | 5.59121295163034e-06 |
| GO:0002478 | antigen processing and presentation of exogenous peptide antigen | 1.10645971464263e-07 | 5.74873762265642e-06 |
| GO:0001818 | negative regulation of cytokine production | 1.21820324252573e-07 | 6.27427635259124e-06 |
| GO:0032757 | positive regulation of interleukin-8 production | 1.28681271582844e-07 | 6.57051009987228e-06 |
| GO:0071222 | cellular response to lipopolysaccharide | 1.43335723185008e-07 | 7.25621784978465e-06 |

| | | | |
|---|---|---|---|
| GO:0019884 | antigen processing and presentation of exogenous antigen | 1.52104578694666e-07 | 7.63487643736023e-06 |
| GO:0070664 | negative regulation of leukocyte proliferation | 1.79439822199438e-07 | 8.93127787300228e-06 |
| GO:0046638 | positive regulation of alpha-beta T cell differentiation | 1.98585157168294e-07 | 9.80183238256505e-06 |
| GO:0007015 | actin filament organization | 2.07798213934988e-07 | 1.00958334311851e-05 |
| GO:0046641 | positive regulation of alpha-beta T cell proliferation | 2.07950646396181e-07 | 1.00958334311851e-05 |
| GO:0002687 | positive regulation of leukocyte migration | 2.10019832555812e-07 | 1.01133940506347e-05 |
| GO:0002286 | T cell activation involved in immune response | 2.45061249050141e-07 | 1.17056272429354e-05 |
| GO:0002718 | regulation of cytokine production involved in immune response | 2.4844119846628e-07 | 1.17721377481262e-05 |
| GO:0031349 | positive regulation of defense response | 2.88954946071226e-07 | 1.35516070183305e-05 |
| GO:0002285 | lymphocyte activation involved in immune response | 2.90571347514431e-07 | 1.35516070183305e-05 |
| GO:0050868 | negative regulation of T cell activation | 4.05322502933988e-07 | 1.87556655068595e-05 |
| GO:0050852 | T cell receptor signaling pathway | 4.2188136826412e-07 | 1.937056855991e-05 |
| GO:0002705 | positive regulation of leukocyte mediated immunity | 4.27118913736713e-07 | 1.94601948158658e-05 |
| GO:0050672 | negative regulation of lymphocyte proliferation | 4.47026376068117e-07 | 2.0211734545431e-05 |
| GO:0031099 | regeneration | 4.64317320183553e-07 | 2.0834480965509e-05 |
| GO:0030888 | regulation of B cell proliferation | 4.8526648210482e-07 | 2.16107772444124e-05 |
| GO:0009615 | response to virus | 4.93190629662607e-07 | 2.17997619365046e-05 |
| GO:0032612 | interleukin-1 production | 5.20979323357925e-07 | 2.28574854240666e-05 |
| GO:0002291 | T cell activation via T cell receptor contact with antigen bound to MHC molecule on antigen presenting cell | 5.43310239052168e-07 | 2.36619598963676e-05 |
| GO:2001185 | regulation of CD8-positive, alpha-beta T cell activation | 5.69056069173258e-07 | 2.46023291803884e-05 |
| GO:0032945 | negative regulation of mononuclear cell proliferation | 5.79414309886351e-07 | 2.48686301264989e-05 |
| GO:0048709 | oligodendrocyte differentiation | 6.18720028240861e-07 | 2.63645951602203e-05 |
| GO:0036037 | CD8-positive, alpha-beta T cell activation | 8.8827872636807e-07 | 3.75805349734148e-05 |
| GO:1901222 | regulation of NIK/NF-kappaB signaling | 9.47017246717908e-07 | 3.9781440796526e-05 |
| GO:0034612 | response to tumor necrosis factor | 1.39563927208624e-06 | 5.78067930669009e-05 |
| GO:0070371 | ERK1 and ERK2 cascade | 1.39563927208624e-06 | 5.78067930669009e-05 |
| GO:0043372 | positive regulation of CD4-positive, alpha-beta T cell differentiation | 1.44061253079337e-06 | 5.88484993521027e-05 |
| GO:0032611 | interleukin-1 beta production | 1.44066054466569e-06 | 5.88484993521027e-05 |
| GO:0030595 | leukocyte chemotaxis | 1.51141060409745e-06 | 6.13156507401998e-05 |

| | | | |
|---|---|---|---|
| GO:0050777 | negative regulation of immune response | 1.58408062976615e-06 | 6.38265957149992e-05 |
| GO:0030316 | osteoclast differentiation | 1.74706200010455e-06 | 6.95658355100821e-05 |
| GO:0043087 | regulation of GTPase activity | 1.75001004406589e-06 | 6.95658355100821e-05 |
| GO:1902563 | regulation of neutrophil activation | 1.80719927500233e-06 | 7.13602753722587e-05 |
| GO:0002706 | regulation of lymphocyte mediated immunity | 1.82315521240663e-06 | 7.15135650535394e-05 |
| GO:0051090 | regulation of DNA-binding transcription factor activity | 1.89193333383574e-06 | 7.37231653704547e-05 |
| GO:0071356 | cellular response to tumor necrosis factor | 2.08365009186578e-06 | 8.06631339485034e-05 |
| GO:0048017 | inositol lipid-mediated signaling | 2.1454737970421e-06 | 8.19963337469959e-05 |
| GO:0007265 | Ras protein signal transduction | 2.14577608151011e-06 | 8.19963337469959e-05 |
| GO:0002367 | cytokine production involved in immune response | 2.18492604888748e-06 | 8.295716017667e-05 |
| GO:0006909 | phagocytosis | 2.25612215235731e-06 | 8.5114722983518e-05 |
| GO:0016042 | lipid catabolic process | 2.63613265529976e-06 | 9.88216058059525e-05 |
| GO:0030225 | macrophage differentiation | 2.70978358966366e-06 | 0.000100893276145883 |
| GO:0030099 | myeloid cell differentiation | 2.72546415386481e-06 | 0.000100893276145883 |
| GO:0046637 | regulation of alpha-beta T cell differentiation | 2.777318909225e-06 | 0.000100920612879385 |
| GO:1901224 | positive regulation of NIK/NF-kappaB signaling | 2.777318909225e-06 | 0.000100920612879385 |
| GO:2000514 | regulation of CD4-positive, alpha-beta T cell activation | 2.777318909225e-06 | 0.000100920612879385 |
| GO:0002886 | regulation of myeloid leukocyte mediated immunity | 2.88408148163047e-06 | 0.000104161064729861 |
| GO:0030866 | cortical actin cytoskeleton organization | 2.93773508302337e-06 | 0.000105455787253015 |
| GO:0002831 | regulation of response to biotic stimulus | 2.98395751833643e-06 | 0.000106469761331968 |
| GO:0002709 | regulation of T cell mediated immunity | 3.07543552207644e-06 | 0.000109076674235082 |
| GO:0021782 | glial cell development | 3.20473975908893e-06 | 0.000112986152339784 |
| GO:0046683 | response to organophosphorus | 3.31644387247714e-06 | 0.000116232526962616 |
| GO:0043300 | regulation of leukocyte degranulation | 3.45517244583202e-06 | 0.000120382272921547 |
| GO:0002429 | immune response-activating cell surface receptor signaling pathway | 3.65849876710076e-06 | 0.000125984233706615 |
| GO:0002757 | immune response-activating signal transduction | 3.65849876710076e-06 | 0.000125984233706615 |
| GO:0051607 | defense response to virus | 3.71963401979461e-06 | 0.000126617197122089 |
| GO:0140546 | defense response to symbiont | 3.71963401979461e-06 | 0.000126617197122089 |
| GO:0014068 | positive regulation of phosphatidylinositol 3-kinase signaling | 3.86747460426152e-06 | 0.000130897440463091 |

| | | | |
|---|---|---|---|
| GO:0032743 | positive regulation of interleukin-2 production | 3.91553858136967e-06 | 0.000131771221690071 |
| GO:0050727 | regulation of inflammatory response | 4.19692151151021e-06 | 0.000140442746399294 |
| GO:2000516 | positive regulation of CD4-positive, alpha-beta T cell activation | 4.3114062974334e-06 | 0.000143463255616281 |
| GO:0045639 | positive regulation of myeloid cell differentiation | 4.40976492940067e-06 | 0.000145916411602459 |
| GO:1903038 | negative regulation of leukocyte cell-cell adhesion | 4.61354557124718e-06 | 0.000151811280102761 |
| GO:0043547 | positive regulation of GTPase activity | 4.90579957861993e-06 | 0.00015927644723292 |
| GO:0032490 | detection of molecule of bacterial origin | 4.91893350471218e-06 | 0.00015927644723292 |
| GO:0022408 | negative regulation of cell-cell adhesion | 4.92836078963593e-06 | 0.00015927644723292 |
| GO:0042590 | antigen processing and presentation of exogenous peptide antigen via MHC class I | 4.94797675010254e-06 | 0.00015927644723292 |
| GO:0032732 | positive regulation of interleukin-1 production | 5.01895430716358e-06 | 0.000160687926277459 |
| GO:0042554 | superoxide anion generation | 5.22783727145401e-06 | 0.000166475699778613 |
| GO:0051056 | regulation of small GTPase mediated signal transduction | 5.49018638564525e-06 | 0.000173895047926079 |
| GO:0071674 | mononuclear cell migration | 5.65636459114165e-06 | 0.000178205571666666 |
| GO:0045123 | cellular extravasation | 6.05064056732819e-06 | 0.000187053525264154 |
| GO:0002483 | antigen processing and presentation of endogenous peptide antigen | 6.06352808555082e-06 | 0.000187053525264154 |
| GO:0034138 | toll-like receptor 3 signaling pathway | 6.06352808555082e-06 | 0.000187053525264154 |
| GO:0098543 | detection of other organism | 6.06352808555082e-06 | 0.000187053525264154 |
| GO:0002456 | T cell mediated immunity | 6.55267423235681e-06 | 0.000201095800405437 |
| GO:0051092 | positive regulation of NF-kappaB transcription factor activity | 6.64544547239007e-06 | 0.000202891616149311 |
| GO:0002576 | platelet degranulation | 6.71614113123458e-06 | 0.000203998481642577 |
| GO:0032722 | positive regulation of chemokine production | 6.9262352554364e-06 | 0.000209306588867091 |
| GO:0097529 | myeloid leukocyte migration | 6.98117840559559e-06 | 0.000209896039067729 |
| GO:0002474 | antigen processing and presentation of peptide antigen via MHC class I | 7.69775219089013e-06 | 0.000230271647609304 |
| GO:0050851 | antigen receptor-mediated signaling pathway | 8.21336529176333e-06 | 0.000244461118709117 |
| GO:0072593 | reactive oxygen species metabolic process | 8.3633160905443e-06 | 0.000247679606021469 |
| GO:0010038 | response to metal ion | 8.57329624869725e-06 | 0.000251384325153633 |
| GO:0032956 | regulation of actin cytoskeleton organization | 8.57329624869725e-06 | 0.000251384325153633 |
| GO:0032731 | positive regulation of interleukin-1 beta production | 9.51500370081421e-06 | 0.000277622497142476 |
| GO:0006636 | unsaturated fatty acid biosynthetic process | 9.69183214392082e-06 | 0.000280023033114356 |

| | | | |
|---|---|---|---|
| GO:0043370 | regulation of CD4-positive, alpha-beta T cell differentiation | 9.69183214392082e-06 | 0.000280023033114356 |
| GO:0006633 | fatty acid biosynthetic process | 9.75636171875489e-06 | 0.000280519079903812 |
| GO:0032642 | regulation of chemokine production | 1.0462462123277e-05 | 0.00029936793795251 |
| GO:0048771 | tissue remodeling | 1.05246979709311e-05 | 0.000299700894624159 |
| GO:0002577 | regulation of antigen processing and presentation | 1.09829802634581e-05 | 0.000310428692316354 |
| GO:0032602 | chemokine production | 1.10077738599523e-05 | 0.000310428692316354 |
| GO:0048732 | gland development | 1.10586618400727e-05 | 0.000310428692316354 |
| GO:2000379 | positive regulation of reactive oxygen species metabolic process | 1.13760781013188e-05 | 0.00031702553812797 |
| GO:1905523 | positive regulation of macrophage migration | 1.14007157895083e-05 | 0.00031702553812797 |
| GO:0046632 | alpha-beta T cell differentiation | 1.15802640088121e-05 | 0.000320513568804645 |
| GO:0002711 | positive regulation of T cell mediated immunity | 1.29984538941761e-05 | 0.000356969962546856 |
| GO:1903053 | regulation of extracellular matrix organization | 1.30179827638225e-05 | 0.000356969962546856 |
| GO:0072330 | monocarboxylic acid biosynthetic process | 1.44893050943779e-05 | 0.000395484580986178 |
| GO:0031343 | positive regulation of cell killing | 1.52529931929778e-05 | 0.000414419626981686 |
| GO:0071260 | cellular response to mechanical stimulus | 1.57281066587869e-05 | 0.000425377058173493 |
| GO:0045807 | positive regulation of endocytosis | 1.62894758935653e-05 | 0.000438557116898125 |
| GO:0030865 | cortical cytoskeleton organization | 1.64236169538888e-05 | 0.000440167797365987 |
| GO:0002888 | positive regulation of myeloid leukocyte mediated immunity | 1.69815322069787e-05 | 0.000451038633461591 |
| GO:0016045 | detection of bacterium | 1.69815322069787e-05 | 0.000451038633461591 |
| GO:1905517 | macrophage migration | 1.72757329064017e-05 | 0.000455363884471937 |
| GO:0034142 | toll-like receptor 4 signaling pathway | 1.72981384444008e-05 | 0.000455363884471937 |
| GO:0048015 | phosphatidylinositol-mediated signaling | 1.76095294092424e-05 | 0.000461509923411252 |
| GO:0022010 | central nervous system myelination | 1.89913135074965e-05 | 0.000493357675021499 |
| GO:0032291 | axon ensheathment in central nervous system | 1.89913135074965e-05 | 0.000493357675021499 |
| GO:0002479 | antigen processing and presentation of exogenous peptide antigen via MHC class I, TAP-dependent | 1.97024307841756e-05 | 0.000507598260076025 |
| GO:0031341 | regulation of cell killing | 1.97108897210005e-05 | 0.000507598260076025 |
| GO:0050730 | regulation of peptidyl-tyrosine phosphorylation | 2.15638187246739e-05 | 0.000552911248078975 |
| GO:0006801 | superoxide metabolic process | 2.23104059235444e-05 | 0.00056958850984498 |
| GO:1905521 | regulation of macrophage migration | 2.26656639725088e-05 | 0.000576174797035063 |

| | | | |
|---|---|---|---|
| GO:0051591 | response to cAMP | 2.33350722010351e-05 | 0.000590656549772355 |
| GO:0071675 | regulation of mononuclear cell migration | 2.37003154222356e-05 | 0.000597348801046389 |
| GO:0045651 | positive regulation of macrophage differentiation | 2.45744062348249e-05 | 0.000616755119190118 |
| GO:0006643 | membrane lipid metabolic process | 2.48773116711635e-05 | 0.000619110575749166 |
| GO:0008154 | actin polymerization or depolymerization | 2.48773116711635e-05 | 0.000619110575749166 |
| GO:0007229 | integrin-mediated signaling pathway | 2.85129220874446e-05 | 0.000705030229364593 |
| GO:0035710 | CD4-positive, alpha-beta T cell activation | 2.85678296551582e-05 | 0.000705030229364593 |
| GO:0019883 | antigen processing and presentation of endogenous antigen | 2.89107446911909e-05 | 0.000710532534464414 |
| GO:0002281 | macrophage activation involved in immune response | 3.07913637054474e-05 | 0.000735456002754019 |
| GO:0032930 | positive regulation of superoxide anion generation | 3.07913637054474e-05 | 0.000735456002754019 |
| GO:0070372 | regulation of ERK1 and ERK2 cascade | 3.10510920822397e-05 | 0.000735456002754019 |
| GO:0031589 | cell-substrate adhesion | 3.11835061829084e-05 | 0.000735456002754019 |
| GO:0097191 | extrinsic apoptotic signaling pathway | 3.13843860088568e-05 | 0.000735456002754019 |
| GO:0014074 | response to purine-containing compound | 3.13925326195148e-05 | 0.000735456002754019 |
| GO:0048260 | positive regulation of receptor-mediated endocytosis | 3.14043783813478e-05 | 0.000735456002754019 |
| GO:0046598 | positive regulation of viral entry into host cell | 3.15081116619695e-05 | 0.000735456002754019 |
| GO:0075294 | positive regulation by symbiont of entry into host | 3.15081116619695e-05 | 0.000735456002754019 |
| GO:0098883 | synapse pruning | 3.15081116619695e-05 | 0.000735456002754019 |
| GO:2000392 | regulation of lamellipodium morphogenesis | 3.15081116619695e-05 | 0.000735456002754019 |
| GO:0030890 | positive regulation of B cell proliferation | 3.15154226220897e-05 | 0.000735456002754019 |
| GO:1903306 | negative regulation of regulated secretory pathway | 3.15390553266117e-05 | 0.000735456002754019 |
| GO:0050920 | regulation of chemotaxis | 3.19039885287028e-05 | 0.000741048329629437 |
| GO:0002688 | regulation of leukocyte chemotaxis | 3.24189432270605e-05 | 0.000747149419197975 |
| GO:0032652 | regulation of interleukin-1 production | 3.24189432270605e-05 | 0.000747149419197975 |
| GO:0051924 | regulation of calcium ion transport | 3.25842937207378e-05 | 0.000748049502743915 |
| GO:0001959 | regulation of cytokine-mediated signaling pathway | 3.31023264320181e-05 | 0.00075700802879 09 |
| GO:0002449 | lymphocyte mediated immunity | 3.40025171150873e-05 | 0.000774603495664086 |
| GO:0045920 | negative regulation of exocytosis | 3.65402582055127e-05 | 0.00082922585958334 |
| GO:0071496 | cellular response to external stimulus | 3.75507651175833e-05 | 0.000848905274013151 |

| | | | |
|---|---|---|---|
| GO:0050921 | positive regulation of chemotaxis | 3.85153124891963e-05 | 0.000865189370081622 |
| GO:0046456 | icosanoid biosynthetic process | 3.85632270304826e-05 | 0.000865189370081622 |
| GO:0010959 | regulation of metal ion transport | 4.05068631188783e-05 | 0.000905366604728741 |
| GO:0042100 | B cell proliferation | 4.09664902277949e-05 | 0.00091219744969635 |
| GO:0006631 | fatty acid metabolic process | 4.1792939324555e-05 | 0.00092711453040951 |
| GO:0001910 | regulation of leukocyte mediated cytotoxicity | 4.61908235384745e-05 | 0.00102053292514275 |
| GO:0033559 | unsaturated fatty acid metabolic process | 4.63487011418875e-05 | 0.00102053292514275 |
| GO:0032651 | regulation of interleukin-1 beta production | 4.86491159328579e-05 | 0.00106721745803821 |
| GO:0031295 | T cell costimulation | 4.96187667446881e-05 | 0.00108447216025383 |
| GO:0006665 | sphingolipid metabolic process | 5.04375770187316e-05 | 0.00109675401642086 |
| GO:0043302 | positive regulation of leukocyte degranulation | 5.05510461730364e-05 | 0.00109675401642086 |
| GO:0046467 | membrane lipid biosynthetic process | 5.53948200999143e-05 | 0.00119310370709743 |
| GO:1903900 | regulation of viral life cycle | 5.53948200999143e-05 | 0.00119310370709743 |
| GO:0014015 | positive regulation of gliogenesis | 5.66130774119639e-05 | 0.00121492484605457 |
| GO:0032088 | negative regulation of NF-kappaB transcription factor activity | 5.87798884138076e-05 | 0.00125687104359199 |
| GO:0032970 | regulation of actin filament-based process | 5.97951391421173e-05 | 0.00127398060841281 |
| GO:0060759 | regulation of response to cytokine stimulus | 6.37296740428562e-05 | 0.00135294214822881 |
| GO:0062207 | regulation of pattern recognition receptor signaling pathway | 6.83183245999233e-05 | 0.00144517655930481 |
| GO:0034154 | toll-like receptor 7 signaling pathway | 6.87329610821299e-05 | 0.00144527508733823 |
| GO:0043367 | CD4-positive, alpha-beta T cell differentiation | 6.88110036517611e-05 | 0.00144527508733823 |
| GO:0002704 | negative regulation of leukocyte mediated immunity | 6.97689809218824e-05 | 0.0014550763169025 |
| GO:0002720 | positive regulation of cytokine production involved in immune response | 6.97689809218824e-05 | 0.0014550763169025 |
| GO:0043903 | regulation of biological process involved in symbiotic interaction | 7.32211089627537e-05 | 0.00152171448556628 |
| GO:0001913 | T cell mediated cytotoxicity | 7.51369884891691e-05 | 0.00155064941749599 |
| GO:0033628 | regulation of cell adhesion mediated by integrin | 7.51369884891691e-05 | 0.00155064941749599 |
| GO:0032753 | positive regulation of interleukin-4 production | 7.85155678755566e-05 | 0.0016147489879407 |
| GO:0001914 | regulation of T cell mediated cytotoxicity | 7.90662080934029e-05 | 0.00162044688767206 |
| GO:0031294 | lymphocyte costimulation | 8.04035851688064e-05 | 0.00164151258274871 |
| GO:0042113 | B cell activation | 8.06483473881265e-05 | 0.00164151258274871 |

| | | | |
|---|---|---|---|
| GO:1901264 | carbohydrate derivative transport | 8.34511849275143e-05 | 0.00169225852436224 |
| GO:0044282 | small molecule catabolic process | 8.37129406783955e-05 | 0.00169225852436224 |
| GO:0072673 | lamellipodium morphogenesis | 8.67776008433031e-05 | 0.00174231772811825 |
| GO:0090594 | inflammatory response to wounding | 8.67776008433031e-05 | 0.00174231772811825 |
| GO:0032928 | regulation of superoxide anion generation | 8.74619538996331e-05 | 0.00174423283820716 |
| GO:0150146 | cell junction disassembly | 8.74619538996331e-05 | 0.00174423283820716 |
| GO:0001909 | leukocyte mediated cytotoxicity | 9.11121950145982e-05 | 0.00181093131232035 |
| GO:0001916 | positive regulation of T cell mediated cytotoxicity | 9.60689835952581e-05 | 0.00190306551784185 |
| GO:0032330 | regulation of chondrocyte differentiation | 9.78538213614761e-05 | 0.00193196061308008 |
| GO:0006066 | alcohol metabolic process | 9.94758848854248e-05 | 0.00195548774219396 |
| GO:0050792 | regulation of viral process | 9.97057737873671e-05 | 0.00195548774219396 |
| GO:0048525 | negative regulation of viral process | 0.000101122234774069 | 0.00197672276094658 |
| GO:0050731 | positive regulation of peptidyl-tyrosine phosphorylation | 0.000101503411520018 | 0.00197764706063509 |
| GO:0002700 | regulation of production of molecular mediator of immune response | 0.000102673313969967 | 0.00199388209391513 |
| GO:0045622 | regulation of T-helper cell differentiation | 0.000107266480153849 | 0.00207627242467729 |
| GO:0002294 | CD4-positive, alpha-beta T cell differentiation involved in immune response | 0.00011021323454588 | 0.00211659840778397 |
| GO:0042130 | negative regulation of T cell proliferation | 0.00011021323454588 | 0.00211659840778397 |
| GO:0014066 | regulation of phosphatidylinositol 3-kinase signaling | 0.000110421899038536 | 0.00211659840778397 |
| GO:0002708 | positive regulation of lymphocyte mediated immunity | 0.000111252266625895 | 0.00212563604911347 |
| GO:0001912 | positive regulation of leukocyte mediated cytotoxicity | 0.00011410111139166 | 0.00216609262092315 |
| GO:0031529 | ruffle organization | 0.00011410111139166 | 0.00216609262092315 |
| GO:0071887 | leukocyte apoptotic process | 0.000115428396516888 | 0.00218428879415185 |
| GO:0033627 | cell adhesion mediated by integrin | 0.000116327919136112 | 0.00219430020714393 |
| GO:2001236 | regulation of extrinsic apoptotic signaling pathway | 0.000122980885338402 | 0.00231243105987097 |
| GO:0046686 | response to cadmium ion | 0.000126855528107578 | 0.00237773826892779 |
| GO:0032479 | regulation of type I interferon production | 0.000127389841130657 | 0.00238022091172519 |
| GO:0110053 | regulation of actin filament organization | 0.000129071456175583 | 0.00240405734254082 |
| GO:0097530 | granulocyte migration | 0.000133615324537574 | 0.00248088892550487 |
| GO:0062197 | cellular response to chemical stress | 0.000135302217155813 | 0.00249509244636835 |

| | | | |
|---|---|---|---|
| GO:0002495 | antigen processing and presentation of peptide antigen via MHC class II | 0.000135715772896846 | 0.00249509244636835 |
| GO:0002287 | alpha-beta T cell activation involved in immune response | 0.000136065314904099 | 0.00249509244636835 |
| GO:0002293 | alpha-beta T cell differentiation involved in immune response | 0.000136065314904099 | 0.00249509244636835 |
| GO:0045577 | regulation of B cell differentiation | 0.000137355903026674 | 0.00251098460995985 |
| GO:0033630 | positive regulation of cell adhesion mediated by integrin | 0.000138814934193735 | 0.00252984878532151 |
| GO:0002292 | T cell differentiation involved in immune response | 0.000141952526234805 | 0.00257909451806366 |
| GO:0032735 | positive regulation of interleukin-12 production | 0.00014373921155803 | 0.00260356987785386 |
| GO:0034109 | homotypic cell-cell adhesion | 0.000145302258453907 | 0.00262385755128808 |
| GO:0032606 | type I interferon production | 0.000146650857920772 | 0.00264016118986241 |
| GO:0019885 | antigen processing and presentation of endogenous peptide antigen via MHC class I | 0.000148818550742259 | 0.00264700383197117 |
| GO:0031643 | positive regulation of myelination | 0.000148818550742259 | 0.00264700383197117 |
| GO:0034134 | toll-like receptor 2 signaling pathway | 0.000148818550742259 | 0.00264700383197117 |
| GO:0150078 | positive regulation of neuroinflammatory response | 0.000148818550742259 | 0.00264700383197117 |
| GO:0032613 | interleukin-10 production | 0.000157855828095588 | 0.00279934152637776 |
| GO:0002504 | antigen processing and presentation of peptide or polysaccharide antigen via MHC class II | 0.000159109454388162 | 0.00281315014430174 |
| GO:2000106 | regulation of leukocyte apoptotic process | 0.000174349518878946 | 0.00306897773098399 |
| GO:0034162 | toll-like receptor 9 signaling pathway | 0.000174615143566032 | 0.00306897773098399 |
| GO:0030183 | B cell differentiation | 0.000178854247290411 | 0.00313418256420446 |
| GO:0042088 | T-helper 1 type immune response | 0.000190392439722236 | 0.00331221460807792 |
| GO:0046636 | negative regulation of alpha-beta T cell activation | 0.000190392439722236 | 0.00331221460807792 |
| GO:0001516 | prostaglandin biosynthetic process | 0.000192804507678313 | 0.00331221460807792 |
| GO:0046457 | prostanoid biosynthetic process | 0.000192804507678313 | 0.00331221460807792 |
| GO:0032060 | bleb assembly | 0.000192928252538727 | 0.00331221460807792 |
| GO:0043301 | negative regulation of leukocyte degranulation | 0.000192928252538727 | 0.00331221460807792 |
| GO:1902946 | protein localization to early endosome | 0.000192928252538727 | 0.00331221460807792 |
| GO:0038061 | NIK/NF-kappaB signaling | 0.000194396284790501 | 0.00332777223934721 |
| GO:0045730 | respiratory burst | 0.000196651246795143 | 0.00335667243448885 |
| GO:0030278 | regulation of ossification | 0.000205207692340213 | 0.00348265089321228 |
| GO:1903509 | liposaccharide metabolic process | 0.000205207692340213 | 0.00348265089321228 |

| | | | |
|---|---|---|---|
| GO:0045649 | regulation of macrophage differentiation | 0.000213082850332513 | 0.00360597063576992 |
| GO:0031960 | response to corticosteroid | 0.000221620608002532 | 0.00373976883532478 |
| GO:0042093 | T-helper cell differentiation | 0.000240182375658685 | 0.00404147787223406 |
| GO:0035589 | G protein-coupled purinergic nucleotide receptor signaling pathway | 0.000246190048335844 | 0.00413083188751617 |
| GO:0031641 | regulation of myelination | 0.000249450110522734 | 0.00417370905261626 |
| GO:0002825 | regulation of T-helper 1 type immune response | 0.000251350524583149 | 0.0041818796547921 |
| GO:0034123 | positive regulation of toll-like receptor signaling pathway | 0.000251350524583149 | 0.0041818796547921 |
| GO:0051091 | positive regulation of DNA-binding transcription factor activity | 0.000254977444374896 | 0.00423034006451684 |
| GO:0071320 | cellular response to cAMP | 0.000259027274625839 | 0.00428552666929844 |
| GO:0019058 | viral life cycle | 0.000263450917572971 | 0.00434657321667049 |
| GO:0033209 | tumor necrosis factor-mediated signaling pathway | 0.000266039009745497 | 0.00435373002329559 |
| GO:0032673 | regulation of interleukin-4 production | 0.000266089864668749 | 0.00435373002329559 |
| GO:0035590 | purinergic nucleotide receptor signaling pathway | 0.000266089864668749 | 0.00435373002329559 |
| GO:0043030 | regulation of macrophage activation | 0.000280424312752306 | 0.00457562866234686 |
| GO:0046486 | glycerolipid metabolic process | 0.000284586106976363 | 0.00463077887807966 |
| GO:0046849 | bone remodeling | 0.000288850609736578 | 0.00468729359306781 |
| GO:0034446 | substrate adhesion-dependent cell spreading | 0.000292403763283139 | 0.00473198767739353 |
| GO:0070265 | necrotic cell death | 0.00029380386927767 | 0.00474169023903513 |
| GO:0007040 | lysosome organization | 0.000300194912791069 | 0.00481452310586111 |
| GO:0080171 | lytic vacuole organization | 0.000300194912791069 | 0.00481452310586111 |
| GO:0034121 | regulation of toll-like receptor signaling pathway | 0.000300755284343848 | 0.00481452310586111 |
| GO:0006672 | ceramide metabolic process | 0.000309706692018685 | 0.0049444548162444 |
| GO:0032331 | negative regulation of chondrocyte differentiation | 0.000317567581577789 | 0.00505632469270387 |
| GO:0031334 | positive regulation of protein-containing complex assembly | 0.000320842196821307 | 0.00509356869104934 |
| GO:0045454 | cell redox homeostasis | 0.000323480564025545 | 0.00509356869104934 |
| GO:0000768 | syncytium formation by plasma membrane fusion | 0.000324206550147831 | 0.00509356869104934 |
| GO:0006968 | cellular defense response | 0.000324206550147831 | 0.00509356869104934 |
| GO:0140253 | cell-cell fusion | 0.000324206550147831 | 0.00509356869104934 |
| GO:0048839 | inner ear development | 0.00033217986432702 | 0.00520502999049985 |

| | | | |
|---|---|---|---|
| GO:0030148 | sphingolipid biosynthetic process | 0.000338212276546833 | 0.00528557074930578 |
| GO:0032615 | interleukin-12 production | 0.000345554132274786 | 0.00535789823419779 |
| GO:0032653 | regulation of interleukin-10 production | 0.000345554132274786 | 0.00535789823419779 |
| GO:0032655 | regulation of interleukin-12 production | 0.000345554132274786 | 0.00535789823419779 |
| GO:0032703 | negative regulation of interleukin-2 production | 0.00035440245424269 | 0.00546259085718899 |
| GO:0061037 | negative regulation of cartilage development | 0.00035440245424269 | 0.00546259085718899 |
| GO:0001885 | endothelial cell development | 0.000357498579285353 | 0.00546259085718899 |
| GO:0061035 | regulation of cartilage development | 0.000357498579285353 | 0.00546259085718899 |
| GO:0072678 | T cell migration | 0.000357498579285353 | 0.00546259085718899 |
| GO:0031532 | actin cytoskeleton reorganization | 0.000359729081266215 | 0.00546259085718899 |
| GO:0002724 | regulation of T cell cytokine production | 0.000361528890092535 | 0.00546259085718899 |
| GO:0032633 | interleukin-4 production | 0.000361528890092535 | 0.00546259085718899 |
| GO:0045589 | regulation of regulatory T cell differentiation | 0.000361528890092535 | 0.00546259085718899 |
| GO:0050869 | negative regulation of B cell activation | 0.000361528890092535 | 0.00546259085718899 |
| GO:0007398 | ectoderm development | 0.000381563125541419 | 0.00573603652939549 |
| GO:1903975 | regulation of glial cell migration | 0.000381563125541419 | 0.00573603652939549 |
| GO:0006690 | icosanoid metabolic process | 0.000385818773004998 | 0.00576832199768899 |
| GO:0045657 | positive regulation of monocyte differentiation | 0.000386632421590837 | 0.00576832199768899 |
| GO:2001269 | positive regulation of cysteine-type endopeptidase activity involved in apoptotic signaling pathway | 0.000386632421590837 | 0.00576832199768899 |
| GO:0002690 | positive regulation of leukocyte chemotaxis | 0.000398930416260598 | 0.00593684637063197 |
| GO:0007599 | hemostasis | 0.000400527749033627 | 0.00594567884091773 |
| GO:2000377 | regulation of reactive oxygen species metabolic process | 0.000410690048674476 | 0.00608129289574731 |
| GO:0150076 | neuroinflammatory response | 0.000415423958716674 | 0.00613605014333881 |
| GO:0038065 | collagen-activated signaling pathway | 0.000419784362747145 | 0.00616968431898595 |
| GO:0060099 | regulation of phagocytosis, engulfment | 0.000419784362747145 | 0.00616968431898595 |
| GO:0050817 | coagulation | 0.000431298474505678 | 0.00632171630910003 |
| GO:0033077 | T cell differentiation in thymus | 0.000433330545584098 | 0.00632171630910003 |
| GO:0042246 | tissue regeneration | 0.000433330545584098 | 0.00632171630910003 |
| GO:0008360 | regulation of cell shape | 0.000443457491269675 | 0.00645355951054124 |

| | | | |
|---|---|---|---|
| GO:0001503 | ossification | 0.000444581810878511 | 0.00645406388684662 |
| GO:0006664 | glycolipid metabolic process | 0.000449089315221534 | 0.00648769759526133 |
| GO:0022612 | gland morphogenesis | 0.000449089315221534 | 0.00648769759526133 |
| GO:0006816 | calcium ion transport | 0.000453061729551175 | 0.00652915966941997 |
| GO:0050864 | regulation of B cell activation | 0.000454808390456247 | 0.00653842256473872 |
| GO:0007204 | positive regulation of cytosolic calcium ion concentration | 0.000456318557726008 | 0.00654424895256936 |
| GO:0051928 | positive regulation of calcium ion transport | 0.000477276288024734 | 0.00681182519029036 |
| GO:0072676 | lymphocyte migration | 0.000477276288024734 | 0.00681182519029036 |
| GO:0036336 | dendritic cell migration | 0.000490367438793972 | 0.00698184216340553 |
| GO:0006949 | syncytium formation | 0.000497635047294356 | 0.00706832706264861 |
| GO:0018212 | peptidyl-tyrosine modification | 0.000502007501871257 | 0.00711337424302261 |
| GO:0019886 | antigen processing and presentation of exogenous peptide antigen via MHC class II | 0.000513311910154874 | 0.00725619676335875 |
| GO:0070527 | platelet aggregation | 0.000515646786546718 | 0.00727184742075288 |
| GO:0046513 | ceramide biosynthetic process | 0.000521352126028377 | 0.00732610482618883 |
| GO:0007596 | blood coagulation | 0.00052196796161602 | 0.00732610482618883 |
| GO:0014013 | regulation of gliogenesis | 0.000543927235538173 | 0.00761626717752388 |
| GO:0030041 | actin filament polymerization | 0.000561588871355391 | 0.00784502567225939 |
| GO:0051048 | negative regulation of secretion | 0.000564167760325189 | 0.00786250739860258 |
| GO:0016485 | protein processing | 0.000569401907149323 | 0.00791682510808788 |
| GO:0032495 | response to muramyl dipeptide | 0.000577630573302609 | 0.00801242596175961 |
| GO:0051403 | stress-activated MAPK cascade | 0.00058876920667508 | 0.00813865535360903 |
| GO:0150077 | regulation of neuroinflammatory response | 0.000589478836180698 | 0.00813865535360903 |
| GO:0070374 | positive regulation of ERK1 and ERK2 cascade | 0.000593761978574967 | 0.00817872604441751 |
| GO:0009100 | glycoprotein metabolic process | 0.000599282208075895 | 0.00823561141167872 |
| GO:0008202 | steroid metabolic process | 0.000613411222000957 | 0.00841026543498071 |
| GO:0001676 | long-chain fatty acid metabolic process | 0.000620917353279371 | 0.00843403327605299 |
| GO:0071248 | cellular response to metal ion | 0.000620983295573195 | 0.00843403327605299 |
| GO:0034599 | cellular response to oxidative stress | 0.000623913259515427 | 0.00843403327605299 |
| GO:0045670 | regulation of osteoclast differentiation | 0.000624710670558784 | 0.00843403327605299 |

| | | | |
|---|---|---|---|
| GO:0071677 | positive regulation of mononuclear cell migration | 0.000624710670558784 | 0.00843403327605299 |
| GO:0014065 | phosphatidylinositol 3-kinase signaling | 0.000625112376867679 | 0.00843403327605299 |
| GO:0051017 | actin filament bundle assembly | 0.000625112376867679 | 0.00843403327605299 |
| GO:0051897 | positive regulation of protein kinase B signaling | 0.00063021733504254 | 0.00848358471694764 |
| GO:0031579 | membrane raft organization | 0.000653612177100537 | 0.00877855992055891 |
| GO:0050798 | activated T cell proliferation | 0.000666822032367731 | 0.00887615731045286 |
| GO:0035666 | TRIF-dependent toll-like receptor signaling pathway | 0.000666873206677616 | 0.00887615731045286 |
| GO:0050901 | leukocyte tethering or rolling | 0.000666873206677616 | 0.00887615731045286 |
| GO:0051125 | regulation of actin nucleation | 0.000666873206677616 | 0.00887615731045286 |
| GO:0002532 | production of molecular mediator involved in inflammatory response | 0.000668409866154948 | 0.00887666286375731 |
| GO:0045591 | positive regulation of regulatory T cell differentiation | 0.000678789622005689 | 0.0089343798469771 |
| GO:0071361 | cellular response to ethanol | 0.000678789622005689 | 0.0089343798469771 |
| GO:1901201 | regulation of extracellular matrix assembly | 0.000678789622005689 | 0.0089343798469771 |
| GO:1905153 | regulation of membrane invagination | 0.000678789622005689 | 0.0089343798469771 |
| GO:1904064 | positive regulation of cation transmembrane transport | 0.000681089886109108 | 0.00894477914728214 |
| GO:0034340 | response to type I interferon | 0.000685224908218622 | 0.00895935349090265 |
| GO:1902106 | negative regulation of leukocyte differentiation | 0.000685224908218622 | 0.00895935349090265 |
| GO:0018108 | peptidyl-tyrosine phosphorylation | 0.000694728537820608 | 0.00906360601213978 |
| GO:0007033 | vacuole organization | 0.000698995212899964 | 0.00909137764199477 |
| GO:0033004 | negative regulation of mast cell activation | 0.000704531882099544 | 0.00909137764199477 |
| GO:0035672 | oligopeptide transmembrane transport | 0.000704531882099544 | 0.00909137764199477 |
| GO:0038063 | collagen-activated tyrosine kinase receptor signaling pathway | 0.000704531882099544 | 0.00909137764199477 |
| GO:1905668 | positive regulation of protein localization to endosome | 0.000704531882099544 | 0.00909137764199477 |
| GO:1902903 | regulation of supramolecular fiber organization | 0.000743839758925792 | 0.00954688369649884 |
| GO:0019722 | calcium-mediated signaling | 0.000744666599321706 | 0.00954688369649884 |
| GO:1901136 | carbohydrate derivative catabolic process | 0.000744666599321706 | 0.00954688369649884 |
| GO:0051384 | response to glucocorticoid | 0.000750602984245328 | 0.00959142193051144 |
| GO:0007520 | myoblast fusion | 0.000754618034715234 | 0.00959142193051144 |
| GO:0046596 | regulation of viral entry into host cell | 0.000754618034715234 | 0.00959142193051144 |

| | | | |
|---|---|---|---|
| GO:1905314 | semi-lunar valve development | 0.000754618034715234 | 0.00959142193051144 |
| GO:0043433 | negative regulation of DNA-binding transcription factor activity | 0.000774289186752628 | 0.00982037441784972 |
| GO:0019318 | hexose metabolic process | 0.000809067410297633 | 0.0102395433145147 |
| GO:0051047 | positive regulation of secretion | 0.000818570075043174 | 0.0103377197323683 |
| GO:0019079 | viral genome replication | 0.000825814429805667 | 0.0104053427871482 |
| GO:0097028 | dendritic cell differentiation | 0.000834237132800248 | 0.0104053427871482 |
| GO:0002369 | T cell cytokine production | 0.000834541428474412 | 0.0104053427871482 |
| GO:0003176 | aortic valve development | 0.000834541428474412 | 0.0104053427871482 |
| GO:0006691 | leukotriene metabolic process | 0.000834541428474412 | 0.0104053427871482 |
| GO:0045066 | regulatory T cell differentiation | 0.000834541428474412 | 0.0104053427871482 |
| GO:0046395 | carboxylic acid catabolic process | 0.000836222044011912 | 0.0104053427871482 |
| GO:0016054 | organic acid catabolic process | 0.000848850008886252 | 0.0105182815954671 |
| GO:0045637 | regulation of myeloid cell differentiation | 0.000848850008886252 | 0.0105182815954671 |
| GO:0046209 | nitric oxide metabolic process | 0.000857813564980124 | 0.0106071602199943 |
| GO:0002230 | positive regulation of defense response to virus by host | 0.000892620206201093 | 0.0110145614194356 |
| GO:0046470 | phosphatidylcholine metabolic process | 0.000903687018666666 | 0.0111249249288825 |
| GO:0035821 | modulation of process of other organism | 0.000906806870621711 | 0.0111249249288825 |
| GO:0002719 | negative regulation of cytokine production involved in immune response | 0.000907198841912923 | 0.0111249249288825 |
| GO:0045687 | positive regulation of glial cell differentiation | 0.000956685204392232 | 0.0116615259764105 |
| GO:0062208 | positive regulation of pattern recognition receptor signaling pathway | 0.000956685204392232 | 0.0116615259764105 |
| GO:0006022 | aminoglycan metabolic process | 0.000956863350419639 | 0.0116615259764105 |
| GO:0016125 | sterol metabolic process | 0.000963913583352263 | 0.0117233268053295 |
| GO:0061572 | actin filament bundle organization | 0.000967164268157631 | 0.011738758115364 |
| GO:2000116 | regulation of cysteine-type endopeptidase activity | 0.000970181547250559 | 0.0117512991909306 |
| GO:0022604 | regulation of cell morphogenesis | 0.000982030292593895 | 0.0118705416796605 |
| GO:0032147 | activation of protein kinase activity | 0.000990095329015545 | 0.0119436550585724 |
| GO:2001057 | reactive nitrogen species metabolic process | 0.00100745866487764 | 0.0121284099025818 |
| GO:0071621 | granulocyte chemotaxis | 0.00102462982849711 | 0.0122914087655616 |
| GO:0060284 | regulation of cell development | 0.00102824498785819 | 0.0122914087655616 |

| | | | |
|---|---|---|---|
| GO:0071241 | cellular response to inorganic substance | 0.00102867023729891 | 0.0122914087655616 |
| GO:0006692 | prostanoid metabolic process | 0.00103552472970036 | 0.0122914087655616 |
| GO:0006693 | prostaglandin metabolic process | 0.00103552472970036 | 0.0122914087655616 |
| GO:0052372 | modulation by symbiont of entry into host | 0.00103552472970036 | 0.0122914087655616 |
| GO:0061028 | establishment of endothelial barrier | 0.00103552472970036 | 0.0122914087655616 |
| GO:0048708 | astrocyte differentiation | 0.00104850593099267 | 0.0124206012585392 |
| GO:0048713 | regulation of oligodendrocyte differentiation | 0.00107616314053842 | 0.0126591329933516 |
| GO:0030199 | collagen fibril organization | 0.00107719112420213 | 0.0126591329933516 |
| GO:0045071 | negative regulation of viral genome replication | 0.00107719112420213 | 0.0126591329933516 |
| GO:0061756 | leukocyte adhesion to vascular endothelial cell | 0.00107719112420213 | 0.0126591329933516 |
| GO:0031098 | stress-activated protein kinase signaling cascade | 0.00107994878262088 | 0.0126664091870563 |
| GO:0045576 | mast cell activation | 0.00108883536696755 | 0.0127453989694641 |
| GO:0070304 | positive regulation of stress-activated protein kinase signaling cascade | 0.00112533638729194 | 0.0131466813055822 |
| GO:0045088 | regulation of innate immune response | 0.001166528429185 | 0.0135036975680187 |
| GO:0090322 | regulation of superoxide metabolic process | 0.00117740672507943 | 0.0135036975680187 |
| GO:2000515 | negative regulation of CD4-positive, alpha-beta T cell activation | 0.00117740672507943 | 0.0135036975680187 |
| GO:0001776 | leukocyte homeostasis | 0.0011788011967687 | 0.0135036975680187 |
| GO:1903707 | negative regulation of hemopoiesis | 0.0011829278930598 | 0.0135036975680187 |
| GO:1902905 | positive regulation of supramolecular fiber organization | 0.00118636487289768 | 0.0135036975680187 |
| GO:0001771 | immunological synapse formation | 0.00119237444336887 | 0.0135036975680187 |
| GO:0006857 | oligopeptide transport | 0.00119237444336887 | 0.0135036975680187 |
| GO:0036109 | alpha-linolenic acid metabolic process | 0.00119237444336887 | 0.0135036975680187 |
| GO:0060100 | positive regulation of phagocytosis, engulfment | 0.00119237444336887 | 0.0135036975680187 |
| GO:0072683 | T cell extravasation | 0.00119237444336887 | 0.0135036975680187 |
| GO:1903054 | negative regulation of extracellular matrix organization | 0.00119237444336887 | 0.0135036975680187 |
| GO:1903977 | positive regulation of glial cell migration | 0.00119237444336887 | 0.0135036975680187 |
| GO:1905155 | positive regulation of membrane invagination | 0.00119237444336887 | 0.0135036975680187 |
| GO:1905666 | regulation of protein localization to endosome | 0.00119237444336887 | 0.0135036975680187 |
| GO:2001198 | regulation of dendritic cell differentiation | 0.00119237444336887 | 0.0135036975680187 |

| | | | |
|---|---|---|---|
| GO:0014003 | oligodendrocyte development | 0.00120179667808256 | 0.0135327789435039 |
| GO:0046006 | regulation of activated T cell proliferation | 0.00120179667808256 | 0.0135327789435039 |
| GO:0097178 | ruffle assembly | 0.00120179667808256 | 0.0135327789435039 |
| GO:0001911 | negative regulation of leukocyte mediated cytotoxicity | 0.00121029274732487 | 0.0135768256484947 |
| GO:0045624 | positive regulation of T-helper cell differentiation | 0.00121029274732487 | 0.0135768256484947 |
| GO:0097305 | response to alcohol | 0.00125110181472026 | 0.0140080832676523 |
| GO:0009395 | phospholipid catabolic process | 0.00127582022382052 | 0.0142450478613143 |
| GO:0006687 | glycosphingolipid metabolic process | 0.00127948091545149 | 0.0142450478613143 |
| GO:0050848 | regulation of calcium-mediated signaling | 0.00127948091545149 | 0.0142450478613143 |
| GO:0002702 | positive regulation of production of molecular mediator of immune response | 0.00128425637623642 | 0.014271389336676 |
| GO:0045471 | response to ethanol | 0.00130049115293242 | 0.01442473614011 |
| GO:0080164 | regulation of nitric oxide metabolic process | 0.0013058533227248 | 0.0144571387485962 |
| GO:0006694 | steroid biosynthetic process | 0.00136421433831273 | 0.0150470047035872 |
| GO:0046578 | regulation of Ras protein signal transduction | 0.00136421433831273 | 0.0150470047035872 |
| GO:0043010 | camera-type eye development | 0.00140861912112531 | 0.015507901588151 |
| GO:0002832 | negative regulation of response to biotic stimulus | 0.0014677724041456 | 0.0161291576062234 |
| GO:0045124 | regulation of bone resorption | 0.00149668644784478 | 0.0164164330196011 |
| GO:0045685 | regulation of glial cell differentiation | 0.00151275131219707 | 0.016539334317789 |
| GO:0006979 | response to oxidative stress | 0.00151347614388682 | 0.016539334317789 |
| GO:0030851 | granulocyte differentiation | 0.001532136218552 | 0.0167124177209641 |
| GO:0051235 | maintenance of location | 0.00154405404254554 | 0.016811456055142 |
| GO:0030100 | regulation of endocytosis | 0.00154935050046197 | 0.0168322521852857 |
| GO:0008347 | glial cell migration | 0.00156074299551668 | 0.0168322521852857 |
| GO:2000179 | positive regulation of neural precursor cell proliferation | 0.00156074299551668 | 0.0168322521852857 |
| GO:0002643 | regulation of tolerance induction | 0.00156301514467451 | 0.0168322521852857 |
| GO:0034375 | high-density lipoprotein particle remodeling | 0.00156301514467451 | 0.0168322521852857 |
| GO:0045953 | negative regulation of natural killer cell mediated cytotoxicity | 0.00156301514467451 | 0.0168322521852857 |
| GO:0043280 | positive regulation of cysteine-type endopeptidase activity involved in apoptotic process | 0.00165031489192438 | 0.0177293675895796 |
| GO:0002507 | tolerance induction | 0.00165230641726286 | 0.0177293675895796 |

| | | | |
|---|---|---|---|
| GO:0008064 | regulation of actin polymerization or depolymerization | 0.00167956696018285 | 0.017989285904454 |
| GO:1903055 | positive regulation of extracellular matrix organization | 0.00168641283572326 | 0.0180300058230846 |
| GO:1904407 | positive regulation of nitric oxide metabolic process | 0.00173279758110325 | 0.0184925406718461 |
| GO:0007160 | cell-matrix adhesion | 0.00180207985831751 | 0.0191973363324004 |
| GO:0030168 | platelet activation | 0.00180817215760729 | 0.0192276547387935 |
| GO:0060337 | type I interferon signaling pathway | 0.00182215549765201 | 0.0193416254705966 |
| GO:0031331 | positive regulation of cellular catabolic process | 0.00183887155976143 | 0.0194668273998558 |
| GO:0030832 | regulation of actin filament length | 0.00184052394798569 | 0.0194668273998558 |
| GO:0002707 | negative regulation of lymphocyte mediated immunity | 0.00184871130638119 | 0.019494410678144 |
| GO:2000401 | regulation of lymphocyte migration | 0.00184971446920765 | 0.019494410678144 |
| GO:0001906 | cell killing | 0.00188590437235893 | 0.0198338405931501 |
| GO:0046850 | regulation of bone remodeling | 0.00189640180258262 | 0.0198338405931501 |
| GO:0097300 | programmed necrotic cell death | 0.00189640180258262 | 0.0198338405931501 |
| GO:0001921 | positive regulation of receptor recycling | 0.00190201273964363 | 0.0198338405931501 |
| GO:0010935 | regulation of macrophage cytokine production | 0.00190201273964363 | 0.0198338405931501 |
| GO:0043374 | CD8-positive, alpha-beta T cell differentiation | 0.00190201273964363 | 0.0198338405931501 |
| GO:0048880 | sensory system development | 0.0019220775099571 | 0.0199373788763744 |
| GO:0048545 | response to steroid hormone | 0.00192540616533617 | 0.0199373788763744 |
| GO:0071214 | cellular response to abiotic stimulus | 0.00192540616533617 | 0.0199373788763744 |
| GO:0104004 | cellular response to environmental stimulus | 0.00192540616533617 | 0.0199373788763744 |
| GO:0002691 | regulation of cellular extravasation | 0.00196880588891513 | 0.0203157443903211 |
| GO:0002756 | MyD88-independent toll-like receptor signaling pathway | 0.00196880588891513 | 0.0203157443903211 |
| GO:0060395 | SMAD protein signal transduction | 0.00197254865036719 | 0.020318966358478 |
| GO:0032874 | positive regulation of stress-activated MAPK cascade | 0.0020068263294366 | 0.0206361672730086 |
| GO:2001056 | positive regulation of cysteine-type endopeptidase activity | 0.00202285212637072 | 0.0207649101291053 |
| GO:0009636 | response to toxic substance | 0.00203297026398914 | 0.02081161279599 |
| GO:1990266 | neutrophil migration | 0.00203442914213713 | 0.02081161279599 |
| GO:0090527 | actin filament reorganization | 0.00204002536683836 | 0.0208328797375579 |
| GO:0006766 | vitamin metabolic process | 0.00204814779298305 | 0.0208600505528359 |

| | | | |
|---|---|---|---|
| GO:0003158 | endothelium development | 0.00204972976899383 | 0.0208600505528359 |
| GO:0009612 | response to mechanical stimulus | 0.0020724464763592 | 0.0210190076703348 |
| GO:0043583 | ear development | 0.0020724464763592 | 0.0210190076703348 |
| GO:0071357 | cellular response to type I interferon | 0.00207889971913494 | 0.0210484154468996 |
| GO:0019216 | regulation of lipid metabolic process | 0.00210035038704209 | 0.0212293094581063 |
| GO:0052548 | regulation of endopeptidase activity | 0.00212124262827191 | 0.0214039524484745 |
| GO:0045069 | regulation of viral genome replication | 0.00213306347762413 | 0.02148662411219 |
| GO:0150063 | visual system development | 0.00215669401212258 | 0.0216877735718201 |
| GO:2001233 | regulation of apoptotic signaling pathway | 0.00225056189658443 | 0.0225067082524719 |
| GO:0002716 | negative regulation of natural killer cell mediated immunity | 0.00225219548916882 | 0.0225067082524719 |
| GO:0010829 | negative regulation of glucose transmembrane transport | 0.00225219548916882 | 0.0225067082524719 |
| GO:0031102 | neuron projection regeneration | 0.00225713062670409 | 0.0225067082524719 |
| GO:0031638 | zymogen activation | 0.00225713062670409 | 0.0225067082524719 |
| GO:0043303 | mast cell degranulation | 0.00226584869720946 | 0.0225556669471793 |
| GO:0001889 | liver development | 0.00227864080750015 | 0.022637877341815 |
| GO:0048678 | response to axon injury | 0.00228839025789354 | 0.022637877341815 |
| GO:0002820 | negative regulation of adaptive immune response | 0.00228939532800053 | 0.022637877341815 |
| GO:0032608 | interferon-beta production | 0.00228939532800053 | 0.022637877341815 |
| GO:0002407 | dendritic cell chemotaxis | 0.00229911985868525 | 0.0226961448716545 |
| GO:1903532 | positive regulation of secretion by cell | 0.00230826565254976 | 0.0227485149085727 |
| GO:0046579 | positive regulation of Ras protein signal transduction | 0.00243672703250351 | 0.0239348826094831 |
| GO:0070227 | lymphocyte apoptotic process | 0.00243672703250351 | 0.0239348826094831 |
| GO:0008625 | extrinsic apoptotic signaling pathway via death domain receptors | 0.00245349761168386 | 0.0240259307871312 |
| GO:0048872 | homeostasis of number of cells | 0.00245410908766071 | 0.0240259307871312 |
| GO:0010950 | positive regulation of endopeptidase activity | 0.00247734710356824 | 0.0242034440458365 |
| GO:0005996 | monosaccharide metabolic process | 0.00248041373220037 | 0.0242034440458365 |
| GO:0014902 | myotube differentiation | 0.00249528912486794 | 0.0243085484976855 |
| GO:1903531 | negative regulation of secretion by cell | 0.00252899220002796 | 0.024596421676134 |
| GO:0045824 | negative regulation of innate immune response | 0.00257028404949205 | 0.0249570367625269 |

| | | | |
|---|---|---|---|
| GO:0030001 | metal ion transport | 0.00261368537324406 | 0.0253369205658344 |
| GO:0043281 | regulation of cysteine-type endopeptidase activity involved in apoptotic process | 0.00266910013597558 | 0.0258141976562171 |
| GO:0045428 | regulation of nitric oxide biosynthetic process | 0.00267841102576491 | 0.0258141976562171 |
| GO:1902041 | regulation of extrinsic apoptotic signaling pathway via death domain receptors | 0.00267841102576491 | 0.0258141976562171 |
| GO:0032689 | negative regulation of interferon-gamma production | 0.00268414933887089 | 0.0258141976562171 |
| GO:0051817 | modulation of process of other organism involved in symbiotic interaction | 0.00268471142262869 | 0.0258141976562171 |
| GO:0042493 | response to drug | 0.00273678354391641 | 0.0262505736208984 |
| GO:1902743 | regulation of lamellipodium organization | 0.00274680761667849 | 0.0262505736208984 |
| GO:0002279 | mast cell activation involved in immune response | 0.00275668694786405 | 0.0262505736208984 |
| GO:0002448 | mast cell mediated immunity | 0.00275668694786405 | 0.0262505736208984 |
| GO:0002823 | negative regulation of adaptive immune response based on somatic recombination of immune receptors built from immunoglobulin superfamily domains | 0.00275668694786405 | 0.0262505736208984 |
| GO:0045581 | negative regulation of T cell differentiation | 0.00275668694786405 | 0.0262505736208984 |
| GO:0052547 | regulation of peptidase activity | 0.00279532481824382 | 0.0265573606867265 |
| GO:0046718 | viral entry into host cell | 0.0028023552978565 | 0.0265573606867265 |
| GO:0061008 | hepaticobiliary system development | 0.0028023552978565 | 0.0265573606867265 |
| GO:0002828 | regulation of type 2 immune response | 0.00282159570795797 | 0.0266969830323244 |
| GO:0120254 | olefinic compound metabolic process | 0.00282917647216302 | 0.0267229314520303 |
| GO:0002753 | cytoplasmic pattern recognition receptor signaling pathway | 0.00283336163293518 | 0.0267229314520303 |
| GO:0002827 | positive regulation of T-helper 1 type immune response | 0.00289016499090796 | 0.0268735435496826 |
| GO:0010934 | macrophage cytokine production | 0.00289016499090796 | 0.0268735435496826 |
| GO:0030852 | regulation of granulocyte differentiation | 0.00289016499090796 | 0.0268735435496826 |
| GO:0032933 | SREBP signaling pathway | 0.00289016499090796 | 0.0268735435496826 |
| GO:0034638 | phosphatidylcholine catabolic process | 0.00289016499090796 | 0.0268735435496826 |
| GO:0045064 | T-helper 2 cell differentiation | 0.00289016499090796 | 0.0268735435496826 |
| GO:0045579 | positive regulation of B cell differentiation | 0.00289016499090796 | 0.0268735435496826 |
| GO:0097202 | activation of cysteine-type endopeptidase activity | 0.00289016499090796 | 0.0268735435496826 |
| GO:2001044 | regulation of integrin-mediated signaling pathway | 0.00289016499090796 | 0.0268735435496826 |
| GO:0050854 | regulation of antigen receptor-mediated signaling pathway | 0.00300940139102473 | 0.0279383768636982 |

| | | | |
|---|---|---|---|
| GO:0048259 | regulation of receptor-mediated endocytosis | 0.00310486915317159 | 0.0287795618063151 |
| GO:0042092 | type 2 immune response | 0.0031412201056129 | 0.0289171155569467 |
| GO:0002064 | epithelial cell development | 0.00314413489366642 | 0.0289171155569467 |
| GO:0034764 | positive regulation of transmembrane transport | 0.00314413489366642 | 0.0289171155569467 |
| GO:0034767 | positive regulation of ion transmembrane transport | 0.00314413489366642 | 0.0289171155569467 |
| GO:0002726 | positive regulation of T cell cytokine production | 0.00315388429001985 | 0.0289171155569467 |
| GO:0007252 | I-kappaB phosphorylation | 0.00315388429001985 | 0.0289171155569467 |
| GO:0043691 | reverse cholesterol transport | 0.00315388429001985 | 0.0289171155569467 |
| GO:0034113 | heterotypic cell-cell adhesion | 0.00316219492192061 | 0.0289485015804263 |
| GO:0034103 | regulation of tissue remodeling | 0.00321583666525824 | 0.029367080956296 |
| GO:0030324 | lung development | 0.00321783480341653 | 0.029367080956296 |
| GO:0006650 | glycerophospholipid metabolic process | 0.00323958310463187 | 0.029520078044207 |
| GO:0043406 | positive regulation of MAP kinase activity | 0.00325274666864858 | 0.0295425629669371 |
| GO:0044242 | cellular lipid catabolic process | 0.00325274666864858 | 0.0295425629669371 |
| GO:0010952 | positive regulation of peptidase activity | 0.00326534488098767 | 0.0295425629669371 |
| GO:0030834 | regulation of actin filament depolymerization | 0.00327619821185392 | 0.0295425629669371 |
| GO:0070228 | regulation of lymphocyte apoptotic process | 0.00327619821185392 | 0.0295425629669371 |
| GO:0019932 | second-messenger-mediated signaling | 0.00327984654500599 | 0.0295425629669371 |
| GO:0006809 | nitric oxide biosynthetic process | 0.0032819527996361 | 0.0295425629669371 |
| GO:0031100 | animal organ regeneration | 0.0032819527996361 | 0.0295425629669371 |
| GO:0032535 | regulation of cellular component size | 0.00329434299845325 | 0.0296090949618188 |
| GO:0014009 | glial cell proliferation | 0.00333040768725196 | 0.0298426697906102 |
| GO:2001238 | positive regulation of extrinsic apoptotic signaling pathway | 0.00333040768725196 | 0.0298426697906102 |
| GO:1901654 | response to ketone | 0.00338692781524383 | 0.0302600033782279 |
| GO:0002645 | positive regulation of tolerance induction | 0.00341785282121129 | 0.0302600033782279 |
| GO:0019371 | cyclooxygenase pathway | 0.00341785282121129 | 0.0302600033782279 |
| GO:0032493 | response to bacterial lipoprotein | 0.00341785282121129 | 0.0302600033782279 |
| GO:0032957 | inositol trisphosphate metabolic process | 0.00341785282121129 | 0.0302600033782279 |
| GO:0045628 | regulation of T-helper 2 cell differentiation | 0.00341785282121129 | 0.0302600033782279 |

| | | | |
|---|---|---|---|
| GO:0046643 | regulation of gamma-delta T cell activation | 0.00341785282121129 | 0.0302600033782279 |
| GO:0051132 | NK T cell activation | 0.00341785282121129 | 0.0302600033782279 |
| GO:0008630 | intrinsic apoptotic signaling pathway in response to DNA damage | 0.0034321281539559 | 0.030341037396837 |
| GO:0009101 | glycoprotein biosynthetic process | 0.00344287426752802 | 0.0303906770291632 |
| GO:0045669 | positive regulation of osteoblast differentiation | 0.00350841691282469 | 0.0309231449027688 |
| GO:0003180 | aortic valve morphogenesis | 0.0036107220495858 | 0.0317304253704698 |
| GO:0097421 | liver regeneration | 0.0036107220495858 | 0.0317304253704698 |
| GO:0051604 | protein maturation | 0.00363591139871488 | 0.0319044492067974 |
| GO:0050767 | regulation of neurogenesis | 0.00365708632061279 | 0.0320427844334165 |
| GO:0051651 | maintenance of location in cell | 0.00368966676702749 | 0.0322804966929155 |
| GO:2000403 | positive regulation of lymphocyte migration | 0.00390605601860343 | 0.034123259289363 |
| GO:0001501 | skeletal system development | 0.00395669787721055 | 0.034514759244062 |
| GO:0070231 | T cell apoptotic process | 0.00399676037809458 | 0.0348129584109621 |
| GO:0031342 | negative regulation of cell killing | 0.00403626644708607 | 0.0350026444598694 |
| GO:0036152 | phosphatidylethanolamine acyl-chain remodeling | 0.00403626644708607 | 0.0350026444598694 |
| GO:0051016 | barbed-end actin filament capping | 0.00403626644708607 | 0.0350026444598694 |
| GO:0006026 | aminoglycan catabolic process | 0.00407322793187025 | 0.035271533684894 |
| GO:0051960 | regulation of nervous system development | 0.00412785004895899 | 0.035672932490006 |
| GO:0010810 | regulation of cell-substrate adhesion | 0.00413162783861964 | 0.035672932490006 |
| GO:0060541 | respiratory system development | 0.00414743175362762 | 0.0357572609559481 |
| GO:0046890 | regulation of lipid biosynthetic process | 0.00415935680789819 | 0.0358079511238096 |
| GO:0002693 | positive regulation of cellular extravasation | 0.00421699021224933 | 0.0360421833003647 |
| GO:0002830 | positive regulation of type 2 immune response | 0.00421699021224933 | 0.0360421833003647 |
| GO:0045779 | negative regulation of bone resorption | 0.00421699021224933 | 0.0360421833003647 |
| GO:0071501 | cellular response to sterol depletion | 0.00421699021224933 | 0.0360421833003647 |
| GO:0071800 | podosome assembly | 0.00421699021224933 | 0.0360421833003647 |
| GO:0045655 | regulation of monocyte differentiation | 0.00430699095172959 | 0.0367583680217498 |
| GO:0045453 | bone resorption | 0.00434430215054448 | 0.0369798401010366 |
| GO:0032231 | regulation of actin filament bundle assembly | 0.00434542777483057 | 0.0369798401010366 |

| | | | |
|---|---|---|---|
| GO:0051701 | biological process involved in interaction with host | 0.00445705059648964 | 0.0378753381391795 |
| GO:0043491 | protein kinase B signaling | 0.00453046555155482 | 0.0384440508049559 |
| GO:1901889 | negative regulation of cell junction assembly | 0.00456288891569698 | 0.0386637926290032 |
| GO:0051058 | negative regulation of small GTPase mediated signal transduction | 0.00458343286554954 | 0.0387823898037856 |
| GO:0030323 | respiratory tube development | 0.00460455977785458 | 0.0389055742713733 |
| GO:0051480 | regulation of cytosolic calcium ion concentration | 0.00461795350914881 | 0.0389631604482741 |
| GO:0002833 | positive regulation of response to biotic stimulus | 0.00468441746088668 | 0.0394677163880964 |
| GO:0050688 | regulation of defense response to virus | 0.00471003193668327 | 0.0396271578991122 |
| GO:0097581 | lamellipodium organization | 0.00472010798000012 | 0.0396556022206251 |
| GO:0007566 | embryo implantation | 0.00476602902522444 | 0.0399846882668617 |
| GO:0030279 | negative regulation of ossification | 0.0048108593704158 | 0.0401334085224969 |
| GO:0032733 | positive regulation of interleukin-10 production | 0.0048108593704158 | 0.0401334085224969 |
| GO:0045429 | positive regulation of nitric oxide biosynthetic process | 0.0048108593704158 | 0.0401334085224969 |
| GO:0048246 | macrophage chemotaxis | 0.0048108593704158 | 0.0401334085224969 |
| GO:0052126 | movement in host environment | 0.00484899437251899 | 0.0403946465097468 |
| GO:0002062 | chondrocyte differentiation | 0.00487250840876023 | 0.0404199822200096 |
| GO:0007200 | phospholipase C-activating G protein-coupled receptor signaling pathway | 0.00487250840876023 | 0.0404199822200096 |
| GO:2001237 | negative regulation of extrinsic apoptotic signaling pathway | 0.00487250840876023 | 0.0404199822200096 |
| GO:0044409 | entry into host | 0.00489123219799096 | 0.0405185570751055 |
| GO:0010720 | positive regulation of cell development | 0.00496669628658561 | 0.0410862319908472 |
| GO:0030500 | regulation of bone mineralization | 0.004990554977422 | 0.0412260211035851 |
| GO:0043112 | receptor metabolic process | 0.00502063497884813 | 0.0414167423115842 |
| GO:0010803 | regulation of tumor necrosis factor-mediated signaling pathway | 0.00505704148806928 | 0.0416590496993524 |
| GO:0032102 | negative regulation of response to external stimulus | 0.00517853315681938 | 0.0426006276220016 |
| GO:0002675 | positive regulation of acute inflammatory response | 0.0052156952666685 | 0.0427874834688055 |
| GO:0045672 | positive regulation of osteoclast differentiation | 0.0052156952666685 | 0.0427874834688055 |
| GO:1901605 | alpha-amino acid metabolic process | 0.00528964450681489 | 0.0431608543113772 |
| GO:0071900 | regulation of protein serine/threonine kinase activity | 0.00530781909425687 | 0.0431608543113772 |
| GO:0000187 | activation of MAPK activity | 0.00535232966796554 | 0.0431608543113772 |

| | | | |
|---|---|---|---|
| GO:0002863 | positive regulation of inflammatory response to antigenic stimulus | 0.00535593920629111 | 0.0431608543113772 |
| GO:0016554 | cytidine to uridine editing | 0.00535593920629111 | 0.0431608543113772 |
| GO:0032494 | response to peptidoglycan | 0.00535593920629111 | 0.0431608543113772 |
| GO:0035739 | CD4-positive, alpha-beta T cell proliferation | 0.00535593920629111 | 0.0431608543113772 |
| GO:0046185 | aldehyde catabolic process | 0.00535593920629111 | 0.0431608543113772 |
| GO:0046642 | negative regulation of alpha-beta T cell proliferation | 0.00535593920629111 | 0.0431608543113772 |
| GO:0048266 | behavioral response to pain | 0.00535593920629111 | 0.0431608543113772 |
| GO:0061517 | macrophage proliferation | 0.00535593920629111 | 0.0431608543113772 |
| GO:0071801 | regulation of podosome assembly | 0.00535593920629111 | 0.0431608543113772 |
| GO:2000561 | regulation of CD4-positive, alpha-beta T cell proliferation | 0.00535593920629111 | 0.0431608543113772 |
| GO:0001894 | tissue homeostasis | 0.00536542336804726 | 0.043167343908802 |
| GO:0002260 | lymphocyte homeostasis | 0.00537860878012761 | 0.043167343908802 |
| GO:1900024 | regulation of substrate adhesion-dependent cell spreading | 0.00537860878012761 | 0.043167343908802 |
| GO:0006644 | phospholipid metabolic process | 0.00539723752511127 | 0.0432582379719 |
| GO:1990868 | response to chemokine | 0.00541669583741496 | 0.0432970167948837 |
| GO:1990869 | cellular response to chemokine | 0.00541669583741496 | 0.0432970167948837 |
| GO:0030038 | contractile actin filament bundle assembly | 0.00545121314543012 | 0.0434556331902861 |
| GO:0043149 | stress fiber assembly | 0.00545121314543012 | 0.0434556331902861 |
| GO:0043277 | apoptotic cell clearance | 0.00564899124757577 | 0.0449113760528742 |
| GO:0071715 | icosanoid transport | 0.00564899124757577 | 0.0449113760528742 |
| GO:0051057 | positive regulation of small GTPase mediated signal transduction | 0.00569931949401965 | 0.0450703226063007 |
| GO:0034368 | protein-lipid complex remodeling | 0.00569942117712632 | 0.0450703226063007 |
| GO:0034369 | plasma lipoprotein particle remodeling | 0.00569942117712632 | 0.0450703226063007 |
| GO:0055094 | response to lipoprotein particle | 0.00569942117712632 | 0.0450703226063007 |
| GO:0006907 | pinocytosis | 0.00575211678045179 | 0.0453658957265193 |
| GO:1904996 | positive regulation of leukocyte adhesion to vascular endothelial cell | 0.00575211678045179 | 0.0453658957265193 |
| GO:0015748 | organophosphate ester transport | 0.00578513594319112 | 0.0455656385525545 |
| GO:0048247 | lymphocyte chemotaxis | 0.0058610792504635 | 0.0461024865876431 |
| GO:0002701 | negative regulation of production of molecular mediator of immune response | 0.00587225998742013 | 0.0461291722884475 |

| | | | |
|---|---|---|---|
| GO:0046330 | positive regulation of JNK cascade | 0.00589106914733952 | 0.0462156325293933 |
| GO:0010759 | positive regulation of macrophage chemotaxis | 0.00594456521608163 | 0.0463895385702918 |
| GO:0030889 | negative regulation of B cell proliferation | 0.00594456521608163 | 0.0463895385702918 |
| GO:0043217 | myelin maintenance | 0.00594456521608163 | 0.0463895385702918 |
| GO:2001267 | regulation of cysteine-type endopeptidase activity involved in apoptotic signaling pathway | 0.00594456521608163 | 0.0463895385702918 |
| GO:0045446 | endothelial cell differentiation | 0.00599106097342605 | 0.046690860717898 |
| GO:0062013 | positive regulation of small molecule metabolic process | 0.00620365800222348 | 0.0482835276185882 |
| GO:0051051 | negative regulation of transport | 0.00621172514694652 | 0.0482835276185882 |
| GO:2001259 | positive regulation of cation channel activity | 0.00622582757989111 | 0.0483297205186043 |
| GO:2001234 | negative regulation of apoptotic signaling pathway | 0.00642864119705051 | 0.0498387981807987 |

**Online Resource 5 Significantly enriched GO Molecular Function (MF) pathways of upregulated genes in TSC patients tissue**

| **ID** | **Description** | **pvalue** | **p.adjust** |
|---|---|---|---|
| GO:0140375 | immune receptor activity | 4.07185839582541e-09 | 4.3894633506998e-06 |
| GO:0030246 | carbohydrate binding | 1.44485229317616e-07 | 7.78775386021951e-05 |
| GO:0019955 | cytokine binding | 4.4140516446089e-07 | 0.00015861158909628 |
| GO:0038024 | cargo receptor activity | 3.57705682633559e-06 | 0.000964016814697441 |
| GO:0032395 | MHC class II receptor activity | 1.79349875956133e-05 | 0.00386678332561424 |
| GO:0004896 | cytokine receptor activity | 3.97017322369383e-05 | 0.00672337722410337 |
| GO:0023023 | MHC protein complex binding | 4.74054357896313e-05 | 0.00672337722410337 |
| GO:0016798 | hydrolase activity, acting on glycosyl bonds | 4.98951927577245e-05 | 0.00672337722410337 |
| GO:0004620 | phospholipase activity | 5.88254558664445e-05 | 0.00674063038522638 |
| GO:0005178 | integrin binding | 6.54331815732763e-05 | 0.00674063038522638 |
| GO:0016298 | lipase activity | 6.87819427063916e-05 | 0.00674063038522638 |
| GO:0035325 | Toll-like receptor binding | 9.67247550402919e-05 | 0.00868910716111956 |
| GO:0005149 | interleukin-1 receptor binding | 0.000215884971924427 | 0.0158322521862987 |
| GO:0023026 | MHC class II protein complex binding | 0.000215884971924427 | 0.0158322521862987 |
| GO:0030695 | GTPase regulator activity | 0.000226198829338703 | 0.0158322521862987 |
| GO:1901981 | phosphatidylinositol phosphate binding | 0.000234987045436715 | 0.0158322521862987 |
| GO:0052740 | 1-acyl-2-lysophosphatidylserine acylhydrolase activity | 0.000259081783815633 | 0.0164288331148972 |
| GO:0017124 | SH3 domain binding | 0.000307919869085699 | 0.0184409788263547 |
| GO:0044548 | S100 protein binding | 0.000342753824939306 | 0.0184744311642286 |
| GO:0045028 | G protein-coupled purinergic nucleotide receptor activity | 0.000342753824939306 | 0.0184744311642286 |
| GO:0005044 | scavenger receptor activity | 0.000396208053871929 | 0.020338680098759 |
| GO:0042605 | peptide antigen binding | 0.00042197803333555 | 0.0206211012078031 |
| GO:0038187 | pattern recognition receptor activity | 0.000472123819765255 | 0.0206211012078031 |
| GO:0035091 | phosphatidylinositol binding | 0.000488993342544647 | 0.0206211012078031 |
| GO:0016175 | superoxide-generating NAD(P)H oxidase activity | 0.00051648398201362 | 0.0206211012078031 |
| GO:0042608 | T cell receptor binding | 0.00051648398201362 | 0.0206211012078031 |

| | | | |
|---|---|---|---|
| GO:0052739 | phosphatidylserine 1-acylhydrolase activity | 0.00051648398201362 | 0.0206211012078031 |
| GO:0005543 | phospholipid binding | 0.000561617749953876 | 0.0210789409527151 |
| GO:0019956 | chemokine binding | 0.000570174480771412 | 0.0210789409527151 |
| GO:0008081 | phosphoric diester hydrolase activity | 0.000586612456940123 | 0.0210789409527151 |
| GO:0005126 | cytokine receptor binding | 0.000628383820483608 | 0.0218515405961719 |
| GO:0003779 | actin binding | 0.000678828567363725 | 0.0219974736415149 |
| GO:0019865 | immunoglobulin binding | 0.000681645892491063 | 0.0219974736415149 |
| GO:0001540 | amyloid-beta binding | 0.000693797869955016 | 0.0219974736415149 |
| GO:0050664 | oxidoreductase activity, acting on NAD(P)H, oxygen as acceptor | 0.000935030806828316 | 0.0272685190752939 |
| GO:0008970 | phospholipase A1 activity | 0.000936243208986949 | 0.0272685190752939 |
| GO:0016004 | phospholipase activator activity | 0.000936243208986949 | 0.0272685190752939 |
| GO:0030247 | polysaccharide binding | 0.000961227945140229 | 0.0272685190752939 |
| GO:0001637 | G protein-coupled chemoattractant receptor activity | 0.00132697907889172 | 0.0357620861761318 |
| GO:0004950 | chemokine receptor activity | 0.00132697907889172 | 0.0357620861761318 |
| GO:0030169 | low-density lipoprotein particle binding | 0.00143843289126104 | 0.0378202599214487 |
| GO:0004602 | glutathione peroxidase activity | 0.00170915467360924 | 0.0438683032893039 |
| GO:0016614 | oxidoreductase activity, acting on CH-OH group of donors | 0.00198664122158299 | 0.049804633415499 |

**Online Resource 6 Significantly enriched GO Cellular Components (CC) pathways of upregulated genes in TSC patients tissue**

| **ID** | **Description** | **pvalue** | **p.adjust** |
|---|---|---|---|
| GO:0030667 | secretory granule membrane | 1.04702468773677e-15 | 6.51249355772272e-13 |
| GO:0045335 | phagocytic vesicle | 6.97887731854525e-14 | 2.17043084606757e-11 |
| GO:0042611 | MHC protein complex | 6.63626924599969e-13 | 1.03690663924726e-10 |
| GO:0005765 | lysosomal membrane | 8.33526237337026e-13 | 1.03690663924726e-10 |
| GO:0098852 | lytic vacuole membrane | 8.33526237337026e-13 | 1.03690663924726e-10 |
| GO:0005766 | primary lysosome | 3.84650978364361e-12 | 3.41789869346618e-10 |
| GO:0042582 | azurophil granule | 3.84650978364361e-12 | 3.41789869346618e-10 |
| GO:0005774 | vacuolar membrane | 7.57866287808744e-12 | 5.89241038771299e-10 |
| GO:0030139 | endocytic vesicle | 9.47059698682447e-12 | 6.5452348064498e-10 |
| GO:0030055 | cell-substrate junction | 8.53433963318989e-11 | 5.30835925184411e-09 |
| GO:0070820 | tertiary granule | 1.2829950366789e-10 | 7.25475375285704e-09 |
| GO:0005925 | focal adhesion | 4.3981631644794e-10 | 2.20836894725157e-08 |
| GO:0062023 | collagen-containing extracellular matrix | 4.6155621084036e-10 | 2.20836894725157e-08 |
| GO:0042581 | specific granule | 6.17700912559207e-10 | 2.74435691151305e-08 |
| GO:0005775 | vacuolar lumen | 9.16826703336206e-10 | 3.80177472983413e-08 |
| GO:0042613 | MHC class II protein complex | 2.48410961139636e-09 | 9.65697611430334e-08 |
| GO:0034774 | secretory granule lumen | 4.14029973121224e-09 | 1.47381168885477e-07 |
| GO:0071556 | integral component of lumenal side of endoplasmic reticulum membrane | 4.50199712029593e-09 | 1.47381168885477e-07 |
| GO:0098553 | lumenal side of endoplasmic reticulum membrane | 4.50199712029593e-09 | 1.47381168885477e-07 |
| GO:0060205 | cytoplasmic vesicle lumen | 7.11216710828294e-09 | 2.21188397067599e-07 |
| GO:0031983 | vesicle lumen | 9.27474669663636e-09 | 2.74709164062277e-07 |
| GO:0070821 | tertiary granule membrane | 1.26609871007225e-08 | 3.57960635302246e-07 |
| GO:0045121 | membrane raft | 2.65846781941985e-08 | 6.88986243199644e-07 |

| | | | |
|---|---|---|---|
| GO:0098857 | membrane microdomain | 2.65846781941985e-08 | 6.88986243199644e-07 |
| GO:0035579 | specific granule membrane | 3.32389187925041e-08 | 8.26984299557503e-07 |
| GO:0030670 | phagocytic vesicle membrane | 4.4848397539295e-08 | 1.07291166420929e-06 |
| GO:0005769 | early endosome | 7.1082569406625e-08 | 1.63753178410818e-06 |
| GO:0005788 | endoplasmic reticulum lumen | 8.73629853139938e-08 | 1.94070631661801e-06 |
| GO:0030666 | endocytic vesicle membrane | 1.1192556628314e-07 | 2.40061042165907e-06 |
| GO:0098576 | lumenal side of membrane | 2.33842559889498e-07 | 4.84833574170893e-06 |
| GO:0030176 | integral component of endoplasmic reticulum membrane | 3.2118871018652e-07 | 6.4444960560005e-06 |
| GO:0009897 | external side of plasma membrane | 8.97184422251851e-07 | 1.74390222075204e-05 |
| GO:0035577 | azurophil granule membrane | 1.08180327948104e-06 | 2.03903527223395e-05 |
| GO:0031227 | intrinsic component of endoplasmic reticulum membrane | 1.24725612803937e-06 | 2.28174503423672e-05 |
| GO:0043202 | lysosomal lumen | 1.59234541704593e-06 | 2.82982528400734e-05 |
| GO:0035578 | azurophil granule lumen | 1.78284665997456e-06 | 3.08036284028939e-05 |
| GO:0045178 | basal part of cell | 4.31295248309411e-06 | 7.25042282293117e-05 |
| GO:0009925 | basal plasma membrane | 1.22924002666287e-05 | 0.00020120718331166 |
| GO:0005884 | actin filament | 1.48713376105644e-05 | 0.000237178769071052 |
| GO:0030027 | lamellipodium | 3.91691348427422e-05 | 0.000609080046804641 |
| GO:0001772 | immunological synapse | 5.00384665325744e-05 | 0.00075912015081125 |
| GO:0012507 | ER to Golgi transport vesicle membrane | 5.32930984668173e-05 | 0.000789245410627627 |
| GO:0001726 | ruffle | 7.84575981001642e-05 | 0.0011348982794954 |
| GO:0016323 | basolateral plasma membrane | 0.000100694441172749 | 0.00142345323657841 |
| GO:0042383 | sarcolemma | 0.000149662956164924 | 0.0020496100652436 |
| GO:0031252 | cell leading edge | 0.00015157887942316 | 0.0020496100652436 |
| GO:0032432 | actin filament bundle | 0.000168684537840943 | 0.00223237835185248 |
| GO:0031091 | platelet alpha granule | 0.00017371475551348 | 0.00225105370686218 |
| GO:0042641 | actomyosin | 0.000204590116310109 | 0.00254635746357644 |
| GO:0055037 | recycling endosome | 0.000204691114435405 | 0.00254635746357644 |
| GO:0008305 | integrin complex | 0.000216631274718151 | 0.00264205201715078 |
| GO:0098636 | protein complex involved in cell adhesion | 0.000222603176686623 | 0.00266267645959768 |
| GO:0101002 | ficolin-1-rich granule | 0.000251312156120166 | 0.00294936153031591 |
| GO:0001725 | stress fiber | 0.000280889083379422 | 0.0031766001793091 |
| GO:0097517 | contractile actin filament bundle | 0.000280889083379422 | 0.0031766001793091 |
| GO:0002102 | podosome | 0.000394169385871354 | 0.00437809567878539 |
| GO:0031901 | early endosome membrane | 0.000409580095270067 | 0.00446945296943827 |
| GO:0045177 | apical part of cell | 0.000538956988541735 | 0.00577984908401654 |
| GO:0005802 | trans-Golgi network | 0.000619212272355883 | 0.00652796666788745 |
| GO:0042470 | melanosome | 0.000666259339470768 | 0.0067936608057511 |
| GO:0048770 | pigment granule | 0.000666259339470768 | 0.0067936608057511 |
| GO:0030134 | COPII-coated ER to Golgi transport vesicle | 0.000750577660587178 | 0.0074649573784107 |
| GO:0043020 | NADPH oxidase complex | 0.000756096969195939 | 0.0074649573784107 |
| GO:0030175 | filopodium | 0.00107613705512479 | 0.010458707004494 |
| GO:0031301 | integral component of organelle membrane | 0.00109482167109357 | 0.0104766012218492 |
| GO:0005912 | adherens junction | 0.00119005320972773 | 0.0112153499462219 |
| GO:0001931 | uropod | 0.00127802418630292 | 0.0115207397663829 |
| GO:0031254 | cell trailing edge | 0.00127802418630292 | 0.0115207397663829 |
| GO:0071203 | WASH complex | 0.00127802418630292 | 0.0115207397663829 |
| GO:0001891 | phagocytic cup | 0.00135623286310097 | 0.0120510977264115 |

| | | | |
|---|---|---|---|
| GO:0005911 | cell-cell junction | 0.00146601808565117 | 0.0128431443559863 |
| GO:0101003 | ficolin-1-rich granule membrane | 0.00149066853450633 | 0.012877719839763 |
| GO:0030662 | coated vesicle membrane | 0.00157656541535097 | 0.0134332012102508 |
| GO:0043034 | costamere | 0.0016882760590991 | 0.0141906447129681 |
| GO:0055038 | recycling endosome membrane | 0.0017404669136791 | 0.0144342722707787 |
| GO:0030669 | clathrin-coated endocytic vesicle membrane | 0.00192614016882858 | 0.0157639366448866 |
| GO:0032588 | trans-Golgi network membrane | 0.00213008270031145 | 0.0172066420726457 |
| GO:0097197 | tetraspanin-enriched microdomain | 0.00216404493533801 | 0.0172568711510287 |
| GO:0016324 | apical plasma membrane | 0.00220956330191973 | 0.0173968148581528 |
| GO:0098858 | actin-based cell projection | 0.00243074522996339 | 0.0188990441629653 |
| GO:0030665 | clathrin-coated vesicle membrane | 0.00266710185013595 | 0.0204807080343773 |
| GO:0044853 | plasma membrane raft | 0.00293469245312199 | 0.0222607159249009 |
| GO:0005902 | microvillus | 0.00299579895213815 | 0.0224504451593968 |
| GO:0033270 | paranode region of axon | 0.00362119960061825 | 0.0268141208521971 |
| GO:0005581 | collagen trimer | 0.00421213734163727 | 0.0308229344293928 |
| GO:1904724 | tertiary granule lumen | 0.00434811942974872 | 0.0312440363151238 |
| GO:0005604 | basement membrane | 0.00437014655854625 | 0.0312440363151238 |
| GO:0030133 | transport vesicle | 0.00443313893923877 | 0.0313342320478013 |
| GO:0005767 | secondary lysosome | 0.00450294165179782 | 0.0314699967125646 |
| GO:0035580 | specific granule lumen | 0.00488381149879041 | 0.0337525639138626 |
| GO:0031300 | intrinsic component of organelle membrane | 0.00679028827115368 | 0.0464127396116219 |

**Online Resource 7 Significantly enriched GO Biological Processes (BP) pathways of downregulated genes in TSC patients tissue**

| ID | Description | pvalue | p.adjust |
|---|---|---|---|
| GO:0050804 | modulation of chemical synaptic transmission | 1.33599654194654e-08 | 3.24089753725737e-05 |
| GO:0099177 | regulation of trans-synaptic signaling | 1.43339121506297e-08 | 3.24089753725737e-05 |
| GO:0023061 | signal release | 3.93986177799222e-07 | 0.00059386849866936 |
| GO:0006836 | neurotransmitter transport | 1.54135721862852e-06 | 0.00174250433565954 |
| GO:0032409 | regulation of transporter activity | 4.20081751079842e-06 | 0.00306025815119743 |
| GO:0022898 | regulation of transmembrane transporter activity | 4.43197492673336e-06 | 0.00306025815119743 |
| GO:0060079 | excitatory postsynaptic potential | 5.61923940961924e-06 | 0.00306025815119743 |
| GO:0032412 | regulation of ion transmembrane transporter activity | 6.15417805730906e-06 | 0.00306025815119743 |
| GO:0007269 | neurotransmitter secretion | 7.23561336284971e-06 | 0.00306025815119743 |
| GO:0099643 | signal release from synapse | 7.23561336284971e-06 | 0.00306025815119743 |
| GO:0035249 | synaptic transmission, glutamatergic | 7.44423698875978e-06 | 0.00306025815119743 |
| GO:0051966 | regulation of synaptic transmission, glutamatergic | 1.02966592628816e-05 | 0.00360028660868988 |
| GO:0099565 | chemical synaptic transmission, postsynaptic | 1.03502268715101e-05 | 0.00360028660868988 |
| GO:0046888 | negative regulation of hormone secretion | 1.42502565663603e-05 | 0.00460283287093437 |
| GO:0061337 | cardiac conduction | 1.53291797781881e-05 | 0.00462123673046443 |
| GO:0031644 | regulation of nervous system process | 1.84528899875828e-05 | 0.00521524803274058 |
| GO:0001505 | regulation of neurotransmitter levels | 1.963413405247e-05 | 0.00522267965795703 |
| GO:0008016 | regulation of heart contraction | 2.36989180337864e-05 | 0.00595369485271012 |

| | | | |
|---|---|---|---|
| GO:0098815 | modulation of excitatory postsynaptic potential | 3.4571694839448e-05 | 0.00822806337178862 |
| GO:2000311 | regulation of AMPA receptor activity | 3.96183218159035e-05 | 0.00895770256257579 |
| GO:1904062 | regulation of cation transmembrane transport | 4.77072684132743e-05 | 0.00982352380347624 |
| GO:2001257 | regulation of cation channel activity | 4.77924643247406e-05 | 0.00982352380347624 |
| GO:2000463 | positive regulation of excitatory postsynaptic potential | 5.17351797089088e-05 | 0.00984284637271247 |
| GO:0042391 | regulation of membrane potential | 5.40146686302315e-05 | 0.00984284637271247 |
| GO:0099601 | regulation of neurotransmitter receptor activity | 5.44164439004449e-05 | 0.00984284637271247 |
| GO:0060078 | regulation of postsynaptic membrane potential | 6.22187282424914e-05 | 0.0108212726581748 |
| GO:0009914 | hormone transport | 6.74038069166979e-05 | 0.0112888894399003 |
| GO:0035637 | multicellular organismal signaling | 0.00010238243112039 | 0.016534762625943 |
| GO:0010975 | regulation of neuron projection development | 0.000107195771815004 | 0.0167151475912913 |
| GO:0050808 | synapse organization | 0.000118585078806478 | 0.01769222571133 |
| GO:0051668 | localization within membrane | 0.00012128681933906 | 0.01769222571133 |
| GO:0019935 | cyclic-nucleotide-mediated signaling | 0.0001383672836761 | 0.0195530267744789 |
| GO:0060047 | heart contraction | 0.000184001309363777 | 0.0252137551800909 |
| GO:0035418 | protein localization to synapse | 0.000218382075304397 | 0.0290448160154848 |
| GO:0046879 | hormone secretion | 0.000246739402799447 | 0.0318787308416885 |
| GO:0048568 | embryonic organ development | 0.000263943342381169 | 0.0331542165068791 |
| GO:0000381 | regulation of alternative mRNA splicing, via spliceosome | 0.000282060029807531 | 0.0344723095889096 |
| GO:0003015 | heart process | 0.000310901361397677 | 0.0359828172496826 |
| GO:0050773 | regulation of dendrite development | 0.000314981016157931 | 0.0359828172496826 |
| GO:0010469 | regulation of signaling receptor activity | 0.000318291174256369 | 0.0359828172496826 |
| GO:1903522 | regulation of blood circulation | 0.000327075348708414 | 0.0360740177282792 |
| GO:0001704 | formation of primary germ layer | 0.000440030653083436 | 0.0473766336486499 |

**Online Resource 8 Significantly enriched GO Molecular Functions (MF) pathways of downregulated genes in TSC patients tissue**

| **ID** | **Description** | **pvalue** | **p.adjust** |
|---|---|---|---|
| GO:0005245 | voltage-gated calcium channel activity | 9.60333889213829e-07 | 0.000770187779149491 |
| GO:0022843 | voltage-gated cation channel activity | 3.1644816813178e-06 | 0.00126895715420844 |
| GO:0005262 | calcium channel activity | 7.95845254838171e-05 | 0.0212755964793404 |
| GO:0072349 | modified amino acid transmembrane transporter activity | 0.000135483981204829 | 0.0271645382315683 |
| GO:0005261 | cation channel activity | 0.000207146770224205 | 0.0332263419439625 |
| GO:0015085 | calcium ion transmembrane transporter activity | 0.000331984333309968 | 0.0355652933112278 |
| GO:0005104 | fibroblast growth factor receptor binding | 0.000385260254393058 | 0.0355652933112278 |
| GO:0005244 | voltage-gated ion channel activity | 0.000399111770325499 | 0.0355652933112278 |
| GO:0022832 | voltage-gated channel activity | 0.000399111770325499 | 0.0355652933112278 |

**Online Resource 9 Significantly enriched GO Cellular Components (CC) pathways of downregulated genes in TSC patients tissue**

| ID | Description | pvalue | p.adjust |
|---|---|---|---|
| GO:0098984 | neuron to neuron synapse | 4.61687428684333e-07 | 0.000202434933791588 |
| GO:0099572 | postsynaptic specialization | 7.30812035348692e-07 | 0.000202434933791588 |
| GO:0014069 | postsynaptic density | 1.59805187332176e-06 | 0.000295106912606751 |
| GO:0032279 | asymmetric synapse | 2.23167155524426e-06 | 0.000309086510401329 |
| GO:0098978 | glutamatergic synapse | 9.25085120384402e-06 | 0.00102499431338592 |
| GO:0043197 | dendritic spine | 1.68031748671255e-05 | 0.00126451093950171 |
| GO:0034703 | cation channel complex | 1.68761777380091e-05 | 0.00126451093950171 |
| GO:0044309 | neuron spine | 1.82600857689778e-05 | 0.00126451093950171 |
| GO:0043025 | neuronal cell body | 8.96707573240998e-05 | 0.0054792239500706 |
| GO:0097060 | synaptic membrane | 9.89029593875559e-05 | 0.0054792239500706 |
| GO:1990351 | transporter complex | 0.000127387353855138 | 0.00641569036688604 |
| GO:1902495 | transmembrane transporter complex | 0.000144078865110255 | 0.00654100586844213 |
| GO:0098793 | presynapse | 0.000153489307382216 | 0.00654100586844213 |
| GO:0005891 | voltage-gated calcium channel complex | 0.000204830233652694 | 0.00810542496025662 |
| GO:0044305 | calyx of Held | 0.000256601893067274 | 0.00900229112734998 |
| GO:0034704 | calcium channel complex | 0.000261081375360611 | 0.00900229112734998 |
| GO:0034702 | ion channel complex | 0.000276243590550451 | 0.00900229112734998 |
| GO:0098878 | neurotransmitter receptor complex | 0.000598202674308234 | 0.0184113489759312 |
| GO:1990454 | L-type voltage-gated calcium channel complex | 0.000844344748760604 | 0.0246193153059671 |
| GO:0098831 | presynaptic active zone cytoplasmic component | 0.00118260464885611 | 0.0327581487733143 |
| GO:0098798 | mitochondrial protein-containing complex | 0.00149083494522769 | 0.0393296456979114 |
| GO:0031234 | extrinsic component of cytoplasmic side of plasma membrane | 0.00161782054350234 | 0.0404399980119315 |
| GO:0019898 | extrinsic component of membrane | 0.00167891688497189 | 0.0404399980119315 |

**Online Resource 10 $Ca^{2+}$ signaling-related genes of interest identified by gene symbol and ENSEMBL ID.**

| Protein | Gene Symbol | Ensembl ID |
|---|---|---|
| **General $Ca^{2+}$ Homeostasis** | | |
| PMCA1-4 | *ATP2B1* | ENSG00000070961 |
| | *ATP2B2* | ENSG00000157087 |
| | *ATP2B3* | ENSG00000067842 |
| | *ATP2B4* | ENSG00000058668 |
| SERCA1-3 | *ATP2A1* | ENSG00000196296 |
| | *ATP2A2* | ENSG00000174437 |
| | *ATP2A3* | ENSG00000074370 |
| NCX1-3 | *SLC8A1* | ENSG00000183023 |
| | *SLC8A2* | ENSG00000118160 |
| | *SLC8A3* | ENSG00000100678 |
| NHE1 | *SLC9A1* | ENSG00000090020 |
| **$Ca^{2+}$ channels, metabotropic and SOCE** | | |
| $IP_3R1$-3 | *ITPR1* | ENSG00000150995 |
| | *ITPR2* | ENSG00000123104 |
| | *ITPR3* | ENSG00000096433 |
| RyR1-3 | *RYR1* | ENSG00000196218 |
| | *RYR2* | ENSG00000198626 |

| | | |
|---|---|---|
| | *RYR3* | ENSG00000198838 |
| STIM1-2 | *STIM1* | ENSG00000167323 |
| | *STIM2* | ENSG00000109689 |
| ORAI1-3 | *ORAI1* | ENSG00000276045 |
| | *ORAI2* | ENSG00000160991 |
| | *ORAI3* | ENSG00000175938 |
| TRPC1,3,4 (Ca2+ pearmeable) | *TRPC1* | ENSG00000144935 |
| | *TRPC3* | ENSG00000138741 |
| | *TRPC4* | ENSG00000133107 |
| **ER $Ca^{2+}$ homeostasis** | | |
| Calreticulins | *CALR* | ENSG00000179218 |
| | *CALR3* | ENSG00000269058 |
| Calnexin | *CANX* | ENSG00000127022 |
| BiP/GRP78 | *HSPA5* | ENSG00000044574 |
| **Mitochondrial $Ca^{2+}$ homeostasis** | | |
| VDAC1 | *VDAC1* | ENSG00000213585 |
| GRP75 | *HSPA9* | ENSG00000113013 |
| MCU complex | *MCU* | ENSG00000156026 |

**Online Resource 11 UMAP plot of scRNA-seq data with 7 clusters.** Each point represents an individual cell, and the color scheme distinguishes cell types. The spatial arrangement reflects the underlying gene expression patterns, revealing distinct clusters corresponding to cell populations in both control and TSC.

**Online Resource 12 Significantly differentially expressed proteins in TSC primary culture astrocytes**

| **Peak Name** | **p-value** | **Log (Fold Change)** | **neglog** |
|---|---|---|---|
| Q9HA92 | 4.0768e-08 | 2 | 7.38968059368076 |
| Q9UGT4 | 9.3034e-08 | 2 | 7.03135830613554 |
| P29972 | 2.4036e-05 | 2 | 4.6191378046588 |
| Q9UMS6 | 0.00081 | 2 | 3.09151498112135 |
| Q8IVN3 | 0.03096 | 2 | 1.50919904798915 |
| Q15847 | 0.00463 | 1.85967144273677 | 2.33441900898205 |
| P26447 | 4.7431e-10 | 1.79374709559554 | 9.32393771890909 |
| P15090 | 0.00048 | 1.63086071796027 | 3.31875876262441 |
| P35749 | 0.00801 | 1.51030413157874 | 2.09636748391576 |
| P50238 | 0.03218 | 1.49913169641163 | 1.49241396023699 |
| P07197 | 0.01783 | 1.41984679683655 | 1.74884865682465 |
| Q14BN4 | 0.0011 | 1.29912048033143 | 2.95860731484178 |
| O14683 | 0.00434 | 1.26078658508732 | 2.36251027048749 |
| Q9UBY9 | 5.3537e-06 | 1.23989816803744 | 5.27134596859827 |
| P48307 | 0.00323 | 1.20940684416246 | 2.4907974776689 |
| P48960 | 7.3829e-05 | 1.13678848823982 | 4.13177301397525 |
| O95810 | 2.7803e-07 | 1.1265108000119 | 6.5559083402995 |
| Q99685 | 0.00715 | 1.07996350821451 | 2.14569395819892 |
| P05413 | 1.4041e-05 | 1.04181012187158 | 4.85260196065235 |
| Q12929 | 9e-04 | 1.03621382765784 | 3.04575749056068 |
| Q96A00 | 0.0175 | 0.957540386073922 | 1.75696195131371 |
| P01889 | 0.00244 | 0.851850602528915 | 2.61261017366127 |
| P00813 | 0.00045 | 0.811751931702616 | 3.34678748622466 |
| P27658 | 0.001 | 0.784366700075886 | 3 |

| | | | |
|---|---|---|---|
| P23497 | 0.00034 | 0.782350500062995 | 3.46852108295774 |
| Q9BUP0 | 0.01198 | 0.761639383490376 | 1.92154318194671 |
| P61769 | 0.03644 | 0.726828416163769 | 1.43842163169904 |
| Q6NZI2 | 0.00128 | 0.714333708312105 | 2.89279003035213 |
| Q16799 | 0.0014 | 0.69953233326808 | 2.85387196432176 |
| Q9BUH8 | 0.00075 | 0.696842794749936 | 3.1249387366083 |
| P15559 | 0.04805 | 0.649921546448693 | 1.31830660799544 |
| Q9H098 | 0.02471 | 0.640384743172495 | 1.60712725459792 |
| Q969G5 | 0.00164 | 0.635237936108073 | 2.7851561519523 |
| Q9NZN4 | 0.00972 | 0.615743802044935 | 2.01233373507373 |
| P0C7U3 | 0.01906 | 0.611819532782861 | 1.71987710369769 |
| P09497-2 | 0.0012 | 0.600995887422807 | 2.92081875395238 |
| P42224 | 0.015 | 0.59821125267767 | 1.82390874094432 |
| P29536 | 0.00141 | 0.588249982733639 | 2.85078088734462 |
| P55201 | 0.00063 | 0.566225841136549 | 3.20065945054642 |
| P27105 | 0.00031 | 0.561052491427051 | 3.50863830616573 |
| Q14847 | 1.9871e-05 | 0.560898972111135 | 4.70178027664691 |
| P21589 | 0.00112 | 0.545629295425041 | 2.95078197732982 |
| P05204 | 0.02879 | 0.545374763844125 | 1.54075833512192 |
| Q9NZN3 | 0.00041 | 0.511555039191916 | 3.38721614328026 |
| Q9BWT7 | 0.00635 | 0.50923199903341 | 2.19722627470802 |
| Q96B97 | 0.01516 | 0.494565101812481 | 1.81930079870397 |
| Q6UVK1 | 0.00026 | 0.491017854511005 | 3.58502665202918 |
| Q9BRF8 | 0.03575 | 0.47067652154677 | 1.4467239538629 |
| P02461 | 0.01605 | 0.470338473311634 | 1.79452496325911 |
| P06396 | 0.01413 | 0.469111810027899 | 1.84985783815144 |
| O95602 | 0.00231 | 0.430953528099113 | 2.63638802010786 |
| P17813 | 0.01111 | 0.398113789562407 | 1.95428594105913 |
| O00151 | 0.00181 | 0.396387712899563 | 2.74232142513082 |
| Q8IVM0 | 0.00271 | 0.394397543363471 | 2.56703070912559 |
| P04439 | 0.00054 | 0.387531946090577 | 3.26760624017703 |
| Q9Y639 | 0.01659 | 0.384000053664212 | 1.78015361397564 |
| Q14764 | 0.03932 | 0.36829883038091 | 1.4053864908399 |
| P02545 | 1.297e-06 | 0.367316739933413 | 5.88706002391592 |
| O94875 | 0.02251 | 0.36435933769562 | 1.64762450499948 |
| Q15746 | 0.0081 | 0.362568374038302 | 2.09151498112135 |
| Q13683-3 | 0.02927 | 0.360570644843012 | 1.53357727756621 |
| P15144 | 0.00942 | 0.359824553618525 | 2.02594909720712 |
| P39060 | 0.00515 | 0.359508000199637 | 2.28819277095881 |
| Q03135 | 0.04532 | 0.358215528433426 | 1.34371009880864 |
| P18065 | 0.00764 | 0.347943682753973 | 2.11690664142431 |
| Q04446 | 0.0402 | 0.346875110801855 | 1.39577394691553 |
| Q8WWX9 | 0.0021 | 0.343769052570045 | 2.67778070526608 |
| O95817 | 0.00902 | 0.326847371296255 | 2.04479346245806 |
| P06703 | 0.0285 | 0.325676512668286 | 1.54515513999149 |
| Q00577 | 0.02059 | 0.321273104079878 | 1.68634365338197 |
| Q9BRK5 | 0.04426 | 0.320961227410534 | 1.35398859040876 |
| Q96Q80-4 | 0.03416 | 0.316485627023464 | 1.46648213798303 |
| P63313 | 0.04833 | 0.315776640740411 | 1.31578320486112 |
| P21926 | 0.01065 | 0.309502976094745 | 1.97265039222524 |
| Q09666 | 0.00121 | 0.295265623648132 | 2.91721462968355 |

| | | | |
|---|---|---|---|
| Q9Y6G9 | 0.00664 | 0.294394675816786 | 2.17783192063198 |
| P04792 | 0.00284 | 0.292146946742978 | 2.54668165995296 |
| P54652 | 0.02473 | 0.289171854155962 | 1.6067758836387 |
| P02042 | 0.03874 | 0.287361728628042 | 1.41184038361691 |
| P42892 | 0.04906 | 0.273128040607493 | 1.30927245612963 |
| Q9UGH3 | 0.02049 | 0.270324248134186 | 1.68845804159881 |
| P09382 | 0.01487 | 0.265625478995363 | 1.82768903147805 |
| P46976 | 0.04896 | 0.254247387546831 | 1.3101585908625 |
| Q8NBV4 | 0.0029 | 0.23366424837032 | 2.53760200210104 |
| P48637 | 0.01871 | 0.226455722366603 | 1.72792621249999 |
| Q99575 | 0.03574 | 0.210170918378437 | 1.44684545183037 |
| Q9Y281 | 0.0022 | 0.209735645427755 | 2.65757731917779 |
| Q16270 | 0.03615 | 0.208957213556279 | 1.44189169836945 |
| Q92882 | 8e-04 | 0.20611168606755 | 3.09691001300806 |
| Q9UL46 | 0.00058 | 0.203116707758466 | 3.23657200643706 |
| Q8IWE2 | 0.04625 | 0.189007254388208 | 1.33488826292495 |
| P26885 | 0.03413 | 0.181982835247576 | 1.46686371172136 |
| P13987 | 0.02139 | 0.17755717383353 | 1.66978921542847 |
| Q92743 | 0.04028 | 0.177270012312993 | 1.39491053811842 |
| Q15404 | 0.00465 | 0.176223125936187 | 2.33254704711005 |
| P06576 | 0.01908 | 0.172207143339814 | 1.71942162963192 |
| Q9UBC2 | 0.01696 | 0.172011067204336 | 1.77057415207931 |
| O60664 | 0.03996 | 0.162991431825751 | 1.39837452044606 |
| Q06323 | 0.00087 | 0.139203382398955 | 3.06048074738138 |
| Q13642-1 | 0.02384 | 0.134456754575769 | 1.6226937489318 |
| Q15942 | 0.01034 | 0.124031452408835 | 1.98547946124208 |
| O75947 | 0.01535 | 0.114652423917978 | 1.81389162018679 |
| P60903 | 0.04904 | 0.106948156143694 | 1.30944953848964 |
| P60660 | 0.03696 | 0.104498887582815 | 1.43226803745193 |
| O15511 | 0.03041 | 0.0903868984750699 | 1.51698357985587 |
| Q99436 | 0.04195 | -0.110563027512219 | 1.37726803483528 |
| Q15370 | 0.03453 | -0.118820543557663 | 1.46180342165055 |
| P63104 | 0.00305 | -0.130896232110636 | 2.51570016065321 |
| P24539 | 0.0171 | -0.13318082381023 | 1.76700388960785 |
| Q15691 | 0.00267 | -0.140750784457149 | 2.57348873863542 |
| P61088 | 3e-04 | -0.141578770633377 | 3.52287874528034 |
| P27824 | 0.03378 | -0.141720076629269 | 1.47134035476501 |
| P62258 | 0.00478 | -0.141746140270261 | 2.32057210338788 |
| O60716 | 0.00157 | -0.141946887328959 | 2.80410034759077 |
| P53990 | 0.03867 | -0.145148570001523 | 1.41262582792693 |
| Q9HC38 | 0.01493 | -0.149159775424219 | 1.82594019227497 |
| O15173 | 0.01125 | -0.154833686006199 | 1.94884747755262 |
| Q7L1Q6 | 0.04141 | -0.159136692686738 | 1.38289476949762 |
| Q9UHX1 | 0.0447 | -0.1618068535371 | 1.34969247686806 |
| P61981 | 0.02521 | -0.165948007430322 | 1.59842715432355 |
| P63241 | 0.01638 | -0.167630119043219 | 1.7856861025756 |
| P35232 | 0.01011 | -0.171978796045288 | 1.995248844409 |
| P13473 | 0.01397 | -0.177953061895383 | 1.85480359388582 |
| P28072 | 0.02046 | -0.182957700482508 | 1.68909437062386 |
| Q13177 | 0.01029 | -0.186180970682587 | 1.98758462523757 |
| Q99623 | 0.02146 | -0.186440232131475 | 1.66837028237007 |

| | | | |
|---|---|---|---|
| P26368 | 0.03907 | -0.189201931753633 | 1.40815658877522 |
| P13667 | 0.03709 | -0.191194613178209 | 1.43074316667139 |
| Q9Y230 | 0.01065 | -0.19211407117217 | 1.97265039222524 |
| P00403 | 0.01864 | -0.192532155505061 | 1.72955409198204 |
| P11233 | 0.03609 | -0.192619343987908 | 1.44261311794049 |
| O75844 | 0.02685 | -0.194798833405802 | 1.57105570996443 |
| P37108 | 0.0222 | -0.194982715929778 | 1.65364702554936 |
| P60468 | 0.02311 | -0.195897275330019 | 1.63620005452089 |
| O15355 | 0.01976 | -0.196233018475853 | 1.70421305974839 |
| Q16643 | 0.00257 | -0.197007444211891 | 2.59006687666871 |
| P20340 | 0.03763 | -0.199345461165932 | 1.42446578168014 |
| P38646 | 0.03236 | -0.201746749718078 | 1.48999148705977 |
| Q07954 | 0.01915 | -0.201761001841849 | 1.71783122169536 |
| Q86Y82 | 0.04257 | -0.202515510864899 | 1.37089634982286 |
| A6NCE7 | 0.00277 | -0.203391975200376 | 2.55752023093555 |
| O60739 | 0.04437 | -0.20749801368562 | 1.35291057128345 |
| P50281 | 0.03786 | -0.208348910765614 | 1.42181939037222 |
| P68104 | 0.02104 | -0.212920189724151 | 1.6769542645183 |
| Q99426 | 0.02169 | -0.219533380793996 | 1.66374044798581 |
| Q86UP2 | 0.0236 | -0.221019109216454 | 1.62708799702989 |
| P26232 | 0.00183 | -0.222861588081444 | 2.73754891026957 |
| P46779 | 0.02785 | -0.224649910153521 | 1.55517480049025 |
| Q9UNF0 | 0.00215 | -0.227798193697239 | 2.66756154008439 |
| Q9UNM6 | 0.02202 | -0.228668471374707 | 1.65718268536427 |
| P18085 | 0.01888 | -0.229545323987439 | 1.72399801003795 |
| Q99729-2 | 0.00268 | -0.229579599662652 | 2.57186520597121 |
| Q9H3N1 | 0.01791 | -0.230765147203873 | 1.74690441415097 |
| P15586 | 0.03941 | -0.231876157464593 | 1.4043935651344 |
| Q07666 | 0.01944 | -0.235853318151874 | 1.71130373940974 |
| P07858 | 0.03468 | -0.239704216193581 | 1.45992091119583 |
| P27348 | 4.1859e-05 | -0.24221367706832 | 4.37821115104916 |
| Q96KP4 | 0.00112 | -0.244909752689134 | 2.95078197732982 |
| O43242 | 0.00707 | -0.246619929899444 | 2.1505805862031 |
| P60983 | 0.01993 | -0.24777320552711 | 1.70049270129951 |
| O94905 | 0.01079 | -0.247927708839235 | 1.96697855531709 |
| P09211 | 0.00013 | -0.252448229004565 | 3.88605664769316 |
| Q06830 | 4e-04 | -0.253185203944578 | 3.39794000867204 |
| P17174 | 0.00805 | -0.254090450162694 | 2.09420411963213 |
| Q12765 | 0.00553 | -0.25446295490093 | 2.2572748686953 |
| Q14108 | 0.02656 | -0.259004325243594 | 1.57577192930402 |
| P51149 | 0.01867 | -0.259737442831016 | 1.72885568205092 |
| Q96FQ6 | 0.03117 | -0.26080075183913 | 1.50626319772316 |
| P40429 | 0.00406 | -0.263174881796053 | 2.39147396642281 |
| Q6IAA8 | 0.0036 | -0.263321191147381 | 2.44369749923271 |
| O00264 | 0.00109 | -0.263886761139938 | 2.96257350205938 |
| O43237 | 0.01653 | -0.265504475580668 | 1.78172714642855 |
| Q99439 | 0.02608 | -0.266844284638918 | 1.58369241294012 |
| P62256 | 0.01657 | -0.270435202679708 | 1.78067749158066 |
| P51665 | 0.01491 | -0.273216848438125 | 1.82652235654701 |
| P60953 | 0.00905 | -0.27386523073174 | 2.0433514207948 |
| P06756 | 0.02061 | -0.273944175687789 | 1.68592200822079 |

| | | | |
|---|---|---|---|
| Q14019 | 0.03829 | -0.274682917657263 | 1.41691463365231 |
| P61916 | 0.03531 | -0.279197218384775 | 1.4521022824369 |
| P18124 | 0.00925 | -0.284432657859781 | 2.03385826726097 |
| P11766 | 0.00259 | -0.288179347662035 | 2.58670023591875 |
| P62877 | 0.00451 | -0.289433594863826 | 2.34582345812204 |
| Q9H0R8-2 | 0.00988 | -0.28947349240992 | 2.00524305541237 |
| Q9HDC9 | 0.00085 | -0.289666658273303 | 3.07058107428571 |
| Q9NZA1 | 4e-04 | -0.292319033114065 | 3.39794000867204 |
| P49591 | 0.02124 | -0.298248411068964 | 1.67284548759057 |
| Q96IZ0 | 0.01217 | -0.298268988804781 | 1.91470942176993 |
| P48723 | 0.00065 | -0.30178303486807 | 3.18708664335714 |
| P54577 | 0.00889 | -0.303975763973758 | 2.05109823902979 |
| P10619 | 0.01383 | -0.305421018199567 | 1.85917781989069 |
| P30626 | 0.00011 | -0.305445328043638 | 3.95860731484178 |
| Q13263 | 0.00434 | -0.307104074825646 | 2.36251027048749 |
| P61204 | 0.036 | -0.307293192159587 | 1.44369749923271 |
| O43681 | 0.00046 | -0.309085524732653 | 3.33724216831843 |
| P35221 | 2.9626e-06 | -0.313674271950337 | 5.52832698149991 |
| Q16881 | 0.0154 | -0.314462119586921 | 1.81247927916354 |
| P57058 | 0.00155 | -0.314741976268442 | 2.80966830182971 |
| P11279 | 0.00885 | -0.314900294847161 | 2.05305672930217 |
| P32119 | 0.00045 | -0.32154962999293 | 3.34678748622466 |
| Q15417 | 0.02116 | -0.322487177693221 | 1.67448433663685 |
| Q96A72 | 0.01341 | -0.322733679174237 | 1.8725712221484 |
| O95197 | 0.01833 | -0.324474409687647 | 1.73683753503778 |
| Q15388 | 0.00334 | -0.324846167086982 | 2.47625353318844 |
| P48147 | 0.0414 | -0.326028134843669 | 1.3829996588791 |
| P84085 | 0.00031 | -0.326664889961416 | 3.50863830616573 |
| Q5JWF2 | 0.00359 | -0.33071230064526 | 2.44490555142168 |
| O94760 | 0.0245 | -0.33886422768855 | 1.61083391563547 |
| P20700 | 0.00973 | -0.340247156814433 | 2.01188715973165 |
| Q8NFC6 | 0.00125 | -0.340664500529761 | 2.90308998699194 |
| Q96PU8 | 0.00161 | -0.342612579327422 | 2.79317412396815 |
| Q14192 | 0.00071 | -0.346300008783728 | 3.14874165128092 |
| Q9UMS4 | 0.00149 | -0.347591824987684 | 2.82681373158773 |
| P15121 | 0.04464 | -0.3479504980357 | 1.35027581407048 |
| P11166 | 0.02009 | -0.349848599758398 | 1.69702006325175 |
| Q9UMR2 | 0.01423 | -0.3540767763862 | 1.84679509991572 |
| P52306 | 0.03165 | -0.358395110642719 | 1.49962628564663 |
| Q9UHB6 | 0.00724 | -0.361764625515131 | 2.14026143380285 |
| P49419 | 0.00058 | -0.362837480553171 | 3.23657200643706 |
| P29323 | 0.01289 | -0.364294029122552 | 1.8897470826466 |
| Q9P0K7 | 0.04759 | -0.370310521867872 | 1.32248429520124 |
| Q15393 | 0.03705 | -0.372406081745669 | 1.43121178768465 |
| P15531 | 0.00129 | -0.374487383374263 | 2.88941028970075 |
| Q13151 | 0.03302 | -0.378224846744173 | 1.48122293107323 |
| O60637 | 0.00898 | -0.379078606857665 | 2.0467236633327 |
| Q16658 | 0.02862 | -0.383757418393498 | 1.54333037057624 |
| P30041 | 0.00116 | -0.389043454968126 | 2.93554201077308 |
| P13611 | 0.00917 | -0.390867432055452 | 2.03763066432998 |
| O75525 | 0.03376 | -0.403888486693165 | 1.47159756204638 |

| | | | |
|---|---|---|---|
| O95865 | 0.01203 | -0.429403867368982 | 1.91973437266016 |
| P53041 | 0.01679 | -0.432545905347275 | 1.77494930386195 |
| P46821 | 0.00105 | -0.434588099020568 | 2.97881070093006 |
| P07339 | 0.00764 | -0.454953689325876 | 2.11690664142431 |
| Q8WX93 | 0.02707 | -0.456789997159961 | 1.56751174422949 |
| P07099 | 0.02002 | -0.458748665056213 | 1.6985359268567 |
| P25205 | 0.0203 | -0.471198051388552 | 1.69250396208679 |
| P35052 | 0.00311 | -0.472260010826086 | 2.50723961097316 |
| O96019 | 0.00149 | -0.480932253583836 | 2.82681373158773 |
| Q9NVJ2 | 0.00044 | -0.492198970908247 | 3.35654732351381 |
| P20908 | 0.0055 | -0.497533850436111 | 2.25963731050576 |
| Q13885 | 0.00712 | -0.49995797803848 | 2.14752000636314 |
| P32322 | 0.03274 | -0.518753002866988 | 1.48492132492408 |
| P63172 | 0.00737 | -0.521483715255785 | 2.13253251214095 |
| Q00796 | 0.03305 | -0.537061043659015 | 1.48082853617834 |
| Q16527 | 0.00463 | -0.572765672188757 | 2.33441900898205 |
| Q9UKX5 | 0.00343 | -0.580106937918487 | 2.46470587995723 |
| P10253 | 0.04234 | -0.58019806384605 | 1.37324914631661 |
| O00483 | 0.01853 | -0.60853182295702 | 1.7321245806811 |
| O75369 | 0.01175 | -0.621092638750147 | 1.92996213339224 |
| P05090 | 0.03672 | -0.623839866194265 | 1.4350973274708 |
| Q15102 | 0.00188 | -0.644108824574167 | 2.72584215073632 |
| O00469 | 0.02173 | -0.647345136557689 | 1.66294027367948 |
| Q14315 | 0.00455 | -0.658310191879942 | 2.34198860334289 |
| P0C0L4 | 0.00042 | -0.66169133564175 | 3.3767507096021 |
| Q06033 | 0.00011 | -0.661820340599306 | 3.95860731484178 |
| Q8IZA3 | 0.00056 | -0.667666959359367 | 3.2518119729938 |
| Q9UBG0 | 1.2368e-05 | -0.669497816532931 | 4.90770052342575 |
| P24821-4 | 7.8302e-05 | -0.675477553941165 | 4.10622714499351 |
| O15061 | 0.04288 | -0.685820619793169 | 1.36774522331529 |
| Q9BRX8 | 0.03346 | -0.691893560137774 | 1.47547406337362 |
| Q8IV08 | 5.0325e-06 | -0.694828016406243 | 5.29821621643931 |
| P01023 | 0.00136 | -0.702124693665469 | 2.86646109162978 |
| Q9H2A2 | 0.00022 | -0.70273373785165 | 3.65757731917779 |
| Q15582 | 0.00189 | -0.704838679092307 | 2.72353819582676 |
| P78330 | 1.5835e-05 | -0.708738927129822 | 4.80038193229207 |
| P46926 | 0.02122 | -0.708976550323341 | 1.67325462043468 |
| P60891 | 0.01278 | -0.718169234848181 | 1.89346914617762 |
| P08195 | 8.008e-05 | -0.742585688809685 | 4.09647593552874 |
| Q96EM0 | 0.02103 | -0.768275023890034 | 1.67716072731368 |
| P30837 | 0.00335 | -0.775772535979865 | 2.47495519296315 |
| Q14195-2 | 0.00085 | -0.783776274180144 | 3.07058107428571 |
| P08729 | 0.00327 | -0.795731315455881 | 2.48545224733971 |
| P05783 | 0.0269 | -0.799331449087839 | 1.57024771999759 |
| P17661 | 0.02548 | -0.79996230467405 | 1.59380057633669 |
| Q9Y2J2 | 9.5997e-05 | -0.800497931759896 | 4.01774233887505 |
| P02511 | 0.01953 | -0.80792680264585 | 1.70929775671215 |
| O00592 | 0.00929 | -0.808704462619558 | 2.03198428600636 |
| P15311 | 0.00999 | -0.813725279251067 | 2.00043451177402 |
| P53801 | 0.00735 | -0.844825086558271 | 2.13371266091581 |
| P53985 | 0.00076 | -0.85914815260585 | 3.11918640771921 |

| | | | |
|---|---|---|---|
| Q9BVA1 | 0.03104 | -0.885696856941491 | 1.50807828741385 |
| Q04695 | 0.02819 | -0.90170711597345 | 1.5499049241284 |
| P28300 | 0.01037 | -0.914447694627514 | 1.98422124361096 |
| P24821 | 4.0315e-06 | -0.949358757945034 | 5.39453333586463 |
| P78386 | 0.00404 | -0.949393278914015 | 2.39361863488939 |
| Q9Y617 | 0.00285 | -0.955027380443442 | 2.54515513999149 |
| P35580 | 0.02565 | -1.00537631750433 | 1.59091263055217 |
| P04216 | 0.02118 | -1.01398343408003 | 1.67407404422853 |
| P09936 | 0.00044 | -1.13096102441855 | 3.35654732351381 |
| Q01650 | 4.3378e-05 | -1.13704741599924 | 4.3627304755811 |
| P49006 | 0.00599 | -1.14701300798263 | 2.22257317761069 |
| P05091 | 0.00287 | -1.18466795827753 | 2.54211810326601 |
| P54687 | 0.02659 | -1.24866336127977 | 1.57528166266843 |
| P12277 | 0.01265 | -1.27702854407412 | 1.89790947448816 |
| Q9P0M6 | 0.00218 | -1.37703747285697 | 2.66154350639539 |
| P19022 | 0.00139 | -1.41940908819593 | 2.85698519974591 |
| Q96CG8 | 0.03798 | -1.56142849798896 | 1.420445039599 |
| Q6DKJ4 | 0.00523 | -1.65558114832841 | 2.28149831113273 |
| P17301 | 0.00525 | -1.69058533870335 | 2.27984069659404 |
| Q9H4G4 | 2.4026e-05 | -1.71901346283718 | 4.61931852726217 |
| O60462 | 0.00592 | -1.7782800971984 | 2.22767829327708 |
| P40121 | 0.00622 | -1.92626018566119 | 2.20620961530918 |
| P12429 | 0.00039 | -2 | 3.4089353929735 |
| O75487 | 0.00244 | -2 | 2.61261017366127 |
| Q13308 | 0.00358 | -2 | 2.44611697335613 |
| P50479 | 0.00557 | -2 | 2.25414480482627 |
| P09455 | 0.01691 | -2 | 1.77185639240226 |
| P17677 | 0.02068 | -2 | 1.6844494655781 |
| Q56VL3 | 0.04497 | -2.28093688186573 | 1.34707711243206 |
| P21980 | 0.00949 | -2.66725183595481 | 2.02273378757271 |
| O15540 | 0.02078 | -2.94169709266011 | 1.68235445677884 |
| P14136 | 0.00957 | -2.96593461871816 | 2.01908806222316 |

**Online Resource 13 Significantly enriched pathways predicted via Ingenuity Pathway Analysis (IPA)**

| **Ingenuity Canonical Pathways** | **-log(p-value)** | **zScore** | **Ratio** |
|---|---|---|---|
| Neutrophil degranulation | 15,9 | -1,85933936 | 0,0735 |
| Phagosome Maturation | 10,7 | | 0,11 |
| Caveolar-mediated Endocytosis Signaling | 8,43 | | 0,147 |
| Protein Ubiquitination Pathway | 6,99 | | 0,0634 |
| Microautophagy Signaling Pathway | 6,8 | -2,121320344 | 0,0828 |
| Gene and protein expression by JAK-STAT signaling after IL-12 stimulation | 6,35 | 1,133893419 | 0,189 |
| Integrin Signaling | 6,19 | 0 | 0,0676 |
| Integrin cell surface interactions | 5,77 | -0,333333333 | 0,106 |
| Germ Cell-Sertoli Cell Junction Signaling | 5,64 | | 0,0714 |
| Virus Entry via Endocytic Pathways | 5,42 | | 0,084 |
| Actin Cytoskeleton Signaling | 5,41 | -1,897366596 | 0,0581 |
| RHO GTPases activate PAKs | 5,2 | -0,447213595 | 0,238 |
| Vitamin-C Transport | 4,99 | -0,447213595 | 0,217 |
| BAG2 Signaling Pathway | 4,92 | -1 | 0,0976 |

| | | | |
|---|---|---|---|
| Hedgehog 'off' state | 4,77 | -1,889822365 | 0,0804 |
| Signaling by ROBO receptors | 4,65 | -2,333333333 | 0,0776 |
| Syndecan interactions | 4,63 | -1,341640786 | 0,185 |
| Aryl Hydrocarbon Receptor Signaling | 4,61 | | 0,068 |
| Semaphorin Neuronal Repulsive Signaling Pathway | 4,61 | 0 | 0,068 |
| Remodeling of Epithelial Adherens Junctions | 4,61 | | 0,106 |
| PAK Signaling | 4,56 | -2,236067977 | 0,0756 |
| MAPK6/MAPK4 signaling | 4,55 | | 0,087 |
| RHOGDI Signaling | 4,49 | 2,645751311 | 0,055 |
| Ferroptosis Signaling Pathway | 4,34 | -1,666666667 | 0,0709 |
| Regulation of RUNX2 expression and activity | 4,32 | -1,133893419 | 0,0959 |
| Natural Killer Cell Signaling | 4,26 | -2,713602101 | 0,0564 |
| COPI-mediated anterograde transport | 4,26 | -1,414213562 | 0,0792 |
| Signaling by Rho Family GTPases | 4,26 | -3 | 0,0487 |
| Triglyceride metabolism | 4,19 | 1,341640786 | 0,152 |
| Regulation of Apoptosis | 4,18 | -1,632993162 | 0,113 |
| Extracellular matrix organization | 4,11 | -0,707106781 | 0,0755 |
| Regulation of Actin-based Motility by Rho | 4 | -2 | 0,0727 |
| Detoxification of Reactive Oxygen Species | 3,94 | -2,236067977 | 0,135 |
| Metabolism of polyamines | 3,92 | -0,816496581 | 0,102 |
| Assembly of collagen fibrils and other multimeric structures | 3,84 | 0 | 0,0984 |
| Collagen degradation | 3,84 | 0 | 0,0984 |
| ERK/MAPK Signaling | 3,84 | -0,377964473 | 0,0507 |
| L1CAM interactions | 3,66 | -2,828427125 | 0,065 |
| Epithelial Adherens Junction Signaling | 3,63 | -2,333333333 | 0,0573 |
| Regulation of Insulin-like Growth Factor (IGF) transport and uptake by IGFBPs | 3,63 | -0,707106781 | 0,0645 |
| Smooth Muscle Contraction | 3,62 | 0,447213595 | 0,116 |
| MHC class II antigen presentation | 3,59 | -2,121320344 | 0,0635 |
| Cellular response to heat stress | 3,54 | -0,377964473 | 0,0722 |
| Mitotic Metaphase and Anaphase | 3,54 | -1,507556723 | 0,0468 |
| Ephrin Receptor Signaling | 3,48 | -1,632993162 | 0,0498 |
| ABC-family proteins mediated transport | 3,43 | -1,133893419 | 0,0693 |
| Cellular response to hypoxia | 3,35 | -1,632993162 | 0,08 |
| Ethanol Degradation II | 3,34 | -2 | 0,143 |
| RHO GTPases activate PKNs | 3,34 | 0 | 0,143 |
| RAC Signaling | 3,3 | -1,341640786 | 0,0576 |
| Myogenesis | 3,28 | -2 | 0,138 |
| Post-translational protein phosphorylation | 3,28 | -1,133893419 | 0,0654 |
| Paxillin Signaling | 3,26 | -2 | 0,0648 |
| Signaling by VEGF | 3,26 | -1,133893419 | 0,0648 |
| Histamine Degradation | 3,24 | | 0,231 |
| Noradrenaline and Adrenaline Degradation | 3,22 | -2 | 0,133 |
| Xenobiotic Metabolism AHR Signaling Pathway | 3,22 | -1,632993162 | 0,0759 |
| Tumor Microenvironment Pathway | 3,2 | -2,121320344 | 0,05 |
| RHO GTPases activate IQGAPs | 3,16 | -2 | 0,129 |
| Signaling by NOTCH4 | 3,14 | -1,632993162 | 0,0732 |
| FAT10 Signaling Pathway | 3,12 | | 0,0909 |
| HSP90 chaperone cycle for steroid hormone receptors in the presence of ligand | 3,12 | -0,447213595 | 0,0909 |
| Sensory processing of sound by outer hair cells of the cochlea | 3,12 | -0,447213595 | 0,0909 |
| Metabolism of nitric oxide: NOS3 activation and regulation | 3,05 | | 0,2 |

| | | | |
|---|---|---|---|
| Hedgehog 'on' state | 3,03 | -1,632993162 | 0,0698 |
| Hepatic Fibrosis / Hepatic Stellate Cell Activation | 3,03 | | 0,0474 |
| Synthesis of DNA | 3,01 | -1,889822365 | 0,0588 |
| Regulation of Cellular Mechanics by Calpain Protease | 2,98 | | 0,0682 |
| TP53 Regulates Metabolic Genes | 2,98 | -2,449489743 | 0,0682 |
| Fatty Acid α-oxidation | 2,96 | | 0,188 |
| NIK-->noncanonical NF-kB signaling | 2,95 | -1,341640786 | 0,0833 |
| Degradation of beta-catenin by the destruction complex | 2,93 | -1,632993162 | 0,0667 |
| ILK Signaling | 2,92 | 0,333333333 | 0,0457 |
| Actin Nucleation by ARP-WASP Complex | 2,9 | | 0,0659 |
| KEAP1-NFE2L2 pathway | 2,9 | -1,632993162 | 0,0659 |
| ROBO SLIT Signaling Pathway | 2,9 | 1,889822365 | 0,0565 |
| EPH-Ephrin signaling | 2,88 | -0,816496581 | 0,0652 |
| Putrescine Degradation III | 2,88 | | 0,176 |
| Sertoli Cell-Sertoli Cell Junction Signaling | 2,86 | -3,16227766 | 0,0413 |
| Antigen Presentation Pathway | 2,83 | | 0,105 |
| Inhibition of Matrix Metalloproteases | 2,83 | | 0,105 |
| Intra-Golgi and retrograde Golgi-to-ER traffic | 2,83 | -2,333333333 | 0,0443 |
| NFE2L2 regulating anti-oxidant/detoxification enzymes | 2,81 | | 0,167 |
| Hedgehog ligand biogenesis | 2,79 | -1,341640786 | 0,0769 |
| CDC42 Signaling | 2,74 | -1,632993162 | 0,0354 |
| Collagen biosynthesis and modifying enzymes | 2,73 | 0,447213595 | 0,0746 |
| Tryptophan Degradation X (Mammalian, via Tryptamine) | 2,73 | | 0,158 |
| TNFR2 non-canonical NF-kB pathway | 2,7 | -1,341640786 | 0,0735 |
| Ethanol Degradation IV | 2,67 | | 0,15 |
| Autophagy | 2,67 | -1,133893419 | 0,0421 |
| Aggrephagy | 2,62 | -1 | 0,093 |
| DNA Replication Pre-Initiation | 2,61 | -1,632993162 | 0,0577 |
| Fructose metabolism | 2,61 | | 0,333 |
| Collagen chain trimerization | 2,59 | 1 | 0,0909 |
| Retinoid metabolism and transport | 2,59 | -2 | 0,0909 |
| Chaperone Mediated Autophagy | 2,55 | | 0,136 |
| Antioxidant Action of Vitamin C | 2,5 | | 0,055 |
| Glycogen Biosynthesis II (from UDP-D-Glucose) | 2,46 | | 0,286 |
| Thioredoxin Pathway | 2,46 | | 0,286 |
| Dopamine Degradation | 2,44 | | 0,125 |
| Glycogen metabolism | 2,44 | | 0,125 |
| Renal Cell Carcinoma Signaling | 2,4 | | 0,0625 |
| CLEAR Signaling Pathway | 2,39 | -1,264911064 | 0,0356 |
| Huntington's Disease Signaling | 2,39 | | 0,0356 |
| Serine biosynthesis | 2,34 | | 0,25 |
| Mitotic G2-G2/M phases | 2,32 | -2,121320344 | 0,0402 |
| Apoptotic execution phase | 2,32 | 0 | 0,0769 |
| Spliceosomal Cycle | 2,29 | -2 | 0,0755 |
| Role of Osteoclasts in Rheumatoid Arthritis Signaling Pathway | 2,26 | 0,632455532 | 0,0341 |
| Axonal Guidance Signaling | 2,23 | | 0,0283 |
| Regulation of mitotic cell cycle | 2,22 | -1,341640786 | 0,0568 |
| TCR signaling | 2,2 | -1,632993162 | 0,0476 |
| Serotonin Degradation | 2,18 | -2 | 0,0702 |
| Oxidative Ethanol Degradation III | 2,16 | | 0,1 |
| Interleukin-1 family signaling | 2,16 | -1,632993162 | 0,0469 |

| | | | |
|---|---|---|---|
| Semaphorin Signaling in Neurons | 2,13 | | 0,0678 |
| Interferon gamma signaling | 2,1 | 2,236067977 | 0,0532 |
| HGF Signaling | 2,09 | | 0,0451 |
| RHO GTPases activate KTN1 | 2,06 | | 0,182 |
| Transcriptional regulation by RUNX3 | 2,06 | -1,341640786 | 0,0521 |
| Retinoate Biosynthesis I | 2,04 | | 0,0909 |
| LPS/IL-1 Mediated Inhibition of RXR Function | 2,02 | | 0,0357 |
| Semaphorin interactions | 2,01 | -1 | 0,0625 |
| Gap junction trafficking and regulation | 2,01 | | 0,0882 |
| Cell Cycle Checkpoints | 2 | -1,666666667 | 0,0331 |
| Tryptophan catabolism | 1,99 | | 0,167 |
| γ-glutamyl Cycle | 1,99 | | 0,167 |
| S Phase | 1,99 | -1,341640786 | 0,05 |
| Pulmonary Fibrosis Idiopathic Signaling Pathway | 1,97 | -1,264911064 | 0,0309 |
| Class I MHC mediated antigen processing and presentation | 1,96 | -0,904534034 | 0,0293 |
| Osteoarthritis Pathway | 1,93 | | 0,0345 |
| C-type lectin receptors (CLRs) | 1,91 | -1,632993162 | 0,0414 |
| Sertoli Cell-Germ Cell Junction Signaling Pathway (Enhanced) | 1,9 | 0 | 0,034 |
| Glucocorticoid Receptor Signaling | 1,89 | | 0,0257 |
| Sensory processing of sound by inner hair cells of the cochlea | 1,89 | -1 | 0,058 |
| Leukocyte Extravasation Signaling | 1,88 | -2,645751311 | 0,0366 |
| Sorbitol Degradation I | 1,88 | | 1 |
| Selenoamino acid metabolism | 1,87 | -2,236067977 | 0,0467 |
| Dilated Cardiomyopathy Signaling Pathway | 1,85 | 0 | 0,04 |
| PTEN Regulation | 1,85 | -1,632993162 | 0,04 |
| Translocation of SLC2A4 (GLUT4) to the plasma membrane | 1,85 | -2 | 0,0563 |
| Phenylalanine Degradation IV (Mammalian, via Side Chain) | 1,85 | | 0,143 |
| Ephrin B Signaling | 1,83 | | 0,0556 |
| PTEN Signaling | 1,83 | | 0,0397 |
| Transcriptional regulation by RUNX1 | 1,82 | -1,632993162 | 0,0395 |
| Neuroprotective Role of THOP1 in Alzheimer's Disease | 1,8 | | 0,0446 |
| TCF dependent signaling in response to WNT | 1,79 | -1,133893419 | 0,0352 |
| SRP-dependent cotranslational protein targeting to membrane | 1,77 | -2,236067977 | 0,0439 |
| Coronavirus Replication Pathway | 1,76 | | 0,0714 |
| Neuregulin Signaling | 1,75 | | 0,0435 |
| Adenosine Nucleotides Degradation II | 1,74 | | 0,125 |
| Triacylglycerol Degradation | 1,73 | | 0,0698 |
| Inhibition of ARE-Mediated mRNA Degradation Pathway | 1,71 | | 0,0373 |
| Signaling by MET | 1,7 | 0 | 0,0506 |
| IL-8 Signaling | 1,7 | -1,632993162 | 0,0337 |
| Cachexia Signaling Pathway | 1,7 | -1,264911064 | 0,028 |
| Glycosaminoglycan metabolism | 1,68 | -1 | 0,05 |
| Cell surface interactions at the vascular wall | 1,67 | -1,889822365 | 0,0332 |
| RHOA Signaling | 1,65 | -1 | 0,041 |
| Deubiquitination | 1,64 | -2,121320344 | 0,0304 |
| NRF2-mediated Oxidative Stress Response | 1,64 | -2 | 0,0327 |
| Metabolism of Angiotensinogen to Angiotensins | 1,64 | | 0,111 |
| Aldosterone Signaling in Epithelial Cells | 1,61 | | 0,0353 |
| Signaling by the B Cell Receptor (BCR) | 1,61 | -0,816496581 | 0,0353 |
| Purine Nucleotides Degradation II (Aerobic) | 1,6 | | 0,105 |
| RHO GTPases Activate ROCKs | 1,6 | | 0,105 |

| | | | |
|---|---|---|---|
| RHO GTPases activate CIT | 1,6 | | 0,105 |
| Chaperone Mediated Autophagy Signaling Pathway | 1,59 | -0,301511345 | 0,0258 |
| IL-17A Signaling in Fibroblasts | 1,58 | 1 | 0,0465 |
| Choline Degradation I | 1,58 | | 0,5 |
| Formaldehyde Oxidation II (Glutathione-dependent) | 1,58 | | 0,5 |
| Thyroid Hormone Biosynthesis | 1,58 | | 0,5 |
| 14-3-3-mediated Signaling | 1,57 | | 0,0391 |
| ABRA Signaling Pathway | 1,55 | 1 | 0,0455 |
| Mitotic G1 phase and G1/S transition | 1,54 | -1,341640786 | 0,0382 |
| Xenobiotic Metabolism Signaling | 1,53 | | 0,029 |
| Xenobiotic Metabolism PXR Signaling Pathway | 1,52 | -1,632993162 | 0,0337 |
| CCR3 Signaling in Eosinophils | 1,52 | | 0,0379 |
| Response to elevated platelet cytosolic Ca2+ | 1,52 | -0,447213595 | 0,0379 |
| Factors involved in megakaryocyte development and platelet production | 1,5 | 1 | 0,044 |
| Phagosome Formation | 1,49 | -1,807392228 | 0,0223 |
| Insertion of tail-anchored proteins into the endoplasmic reticulum membrane | 1,48 | | 0,0909 |
| Interconversion of nucleotide di- and triphosphates | 1,48 | | 0,0909 |
| Cell junction organization | 1,48 | -1 | 0,0435 |
| Sirtuin Signaling Pathway | 1,46 | 0 | 0,0281 |
| RHO GTPase cycle | 1,45 | -1,507556723 | 0,0244 |
| Meiotic synapsis | 1,45 | | 0,0545 |
| RHO GTPases Activate Formins | 1,44 | -1,341640786 | 0,036 |
| Eukaryotic Translation Elongation | 1,44 | -2 | 0,0421 |
| Wound Healing Signaling Pathway | 1,42 | 0,377964473 | 0,0294 |
| Glutathione Redox Reactions I | 1,41 | | 0,0833 |
| Aspartate Biosynthesis | 1,41 | | 0,333 |
| Glutamate Degradation II | 1,41 | | 0,333 |
| Glutathione Biosynthesis | 1,41 | | 0,333 |
| L-cysteine Degradation III | 1,41 | | 0,333 |
| Lactose synthesis | 1,41 | | 0,333 |
| N-acetylglucosamine Degradation I | 1,41 | | 0,333 |
| PRPP Biosynthesis I | 1,41 | | 0,333 |
| Serine Biosynthesis | 1,41 | | 0,333 |
| Signaling by PDGF | 1,39 | | 0,0517 |
| Apelin Liver Signaling Pathway | 1,35 | | 0,0769 |
| Glutathione-mediated Detoxification | 1,35 | | 0,0769 |
| ISGylation Signaling Pathway | 1,35 | 1 | 0,0392 |
| Gap Junction Signaling | 1,35 | | 0,0306 |
| Hepatic Fibrosis Signaling Pathway | 1,33 | 0 | 0,0242 |
| Autism Signaling Pathway | 1,33 | -0,707106781 | 0,0264 |
| PI3K/AKT Signaling | 1,33 | | 0,0303 |
| Endometrial Cancer Signaling | 1,32 | | 0,0484 |
| eNOS Signaling | 1,32 | -1 | 0,0333 |
| D-glucuronate Degradation I | 1,29 | | 0,25 |
| L-cysteine Degradation I | 1,29 | | 0,25 |
| N-acetylglucosamine Degradation II | 1,29 | | 0,25 |
| Oxidized GTP and dGTP Detoxification | 1,29 | | 0,25 |
| Phenylethylamine Degradation I | 1,29 | | 0,25 |
| Proline Biosynthesis I | 1,29 | | 0,25 |
| Retinoate Biosynthesis II | 1,29 | | 0,25 |

| | | | |
|---|---|---|---|
| IGF-1 Signaling | 1,29 | | 0,0377 |
| Clathrin-mediated Endocytosis Signaling | 1,26 | | 0,029 |
| HIF1α Signaling | 1,26 | -0,816496581 | 0,029 |
| Fc epsilon receptor (FCERI) signaling | 1,26 | -1,632993162 | 0,0291 |
| Degradation of the extracellular matrix | 1,25 | | 0,0455 |
| RAF/MAP kinase cascade | 1,24 | -1,889822365 | 0,0267 |
| Agrin Interactions at Neuromuscular Junction | 1,22 | | 0,0441 |
| Choline catabolism | 1,2 | | 0,2 |
| Creatine-phosphate Biosynthesis | 1,2 | | 0,2 |
| Formation of xylulose-5-phosphate | 1,2 | | 0,2 |
| Lysine Degradation II | 1,2 | | 0,2 |
| Lysine Degradation V | 1,2 | | 0,2 |
| Proline Biosynthesis II (from Arginine) | 1,2 | | 0,2 |
| Reelin signalling pathway | 1,2 | | 0,2 |
| Superpathway of Serine and Glycine Biosynthesis I | 1,2 | | 0,2 |
| CDK5 Signaling | 1,2 | | 0,0351 |
| Granzyme A Signaling | 1,18 | | 0,0423 |
| Interferon alpha/beta signaling | 1,18 | | 0,0423 |
| Nonsense-Mediated Decay (NMD) | 1,17 | -2 | 0,0342 |
| Neutrophil Extracellular Trap Signaling Pathway | 1,17 | -2,333333333 | 0,0234 |
| Activation of Matrix Metalloproteinases | 1,16 | | 0,0606 |
| Amyloid fiber formation | 1,15 | | 0,0411 |
| Glioma Invasiveness Signaling | 1,15 | | 0,0411 |
| Oxytocin Signaling Pathway | 1,14 | 1,133893419 | 0,0254 |
| HER-2 Signaling in Breast Cancer | 1,12 | 0,447213595 | 0,0267 |
| Selenocysteine Biosynthesis II (Archaea and Eukaryotes) | 1,12 | | 0,167 |
| Serotonin Receptor Signaling | 1,11 | -1,897366596 | 0,0219 |
| GP6 Signaling Pathway | 1,09 | 1 | 0,032 |
| Xenobiotic Metabolism CAR Signaling Pathway | 1,09 | -2,236067977 | 0,0286 |
| Complement System | 1,09 | | 0,0556 |
| Pancreatic Adenocarcinoma Signaling | 1,08 | 0 | 0,0317 |
| D-myo-inositol (1,4,5,6)-Tetrakisphosphate Biosynthesis | 1,08 | 0 | 0,0282 |
| D-myo-inositol (3,4,5,6)-tetrakisphosphate Biosynthesis | 1,08 | 0 | 0,0282 |
| Protein Sorting Signaling Pathway | 1,08 | -1,341640786 | 0,0282 |
| Role of Osteoblasts in Rheumatoid Arthritis Signaling Pathway | 1,08 | -0,816496581 | 0,0261 |
| Atherosclerosis Signaling | 1,06 | | 0,0312 |
| NF-κB Activation by Viruses | 1,06 | | 0,0375 |
| Tight Junction Signaling | 1,06 | | 0,0279 |
| Regulation of eIF4 and p70S6K Signaling | 1,05 | | 0,0276 |
| Clathrin-mediated endocytosis | 1,05 | 0 | 0,031 |
| G Beta Gamma Signaling | 1,05 | -1 | 0,031 |
| tRNA Charging | 1,05 | | 0,0526 |
| Adenine and Adenosine Salvage III | 1,05 | | 0,143 |
| Arginine Degradation VI (Arginase 2 Pathway) | 1,05 | | 0,143 |
| Aspartate Degradation II | 1,05 | | 0,143 |
| Erythrocytes take up oxygen and release carbon dioxide | 1,05 | | 0,143 |
| Purine Ribonucleosides Degradation to Ribose-1-phosphate | 1,05 | | 0,143 |
| IL-6 Signaling | 1,03 | 0 | 0,0305 |
| SNARE Signaling Pathway | 1,02 | -1 | 0,0303 |
| Acute Phase Response Signaling | 1,02 | | 0,0272 |

| | | | |
|---|---|---|---|
| Immunogenic Cell Death Signaling Pathway | 1,01 | | 0,0357 |
| Miro GTPase Cycle | 1 | | 0,125 |
| Superoxide Radicals Degradation | 1 | | 0,125 |
| Intrinsic Prothrombin Activation Pathway | 0,998 | | 0,0488 |
| 3-phosphoinositide Degradation | 0,993 | 0 | 0,0266 |
| Neddylation | 0,984 | -1,632993162 | 0,0245 |
| DAP12 interactions | 0,98 | | 0,0476 |
| Endothelin-1 Signaling | 0,979 | | 0,0263 |
| Assembly and cell surface presentation of NMDA receptors | 0,963 | | 0,0465 |
| Oncostatin M Signaling | 0,963 | | 0,0465 |
| Vasopressin regulates renal water homeostasis via Aquaporins | 0,963 | | 0,0465 |
| Agranulocyte Adhesion and Diapedesis | 0,958 | | 0,0259 |
| D-myo-inositol-5-phosphate Metabolism | 0,958 | 0 | 0,0259 |
| PDGF Signaling | 0,953 | | 0,0337 |
| Interleukin-9 signaling | 0,951 | | 0,111 |
| Leucine Degradation I | 0,951 | | 0,111 |
| Lysine catabolism | 0,951 | | 0,111 |
| Reelin Signaling in Neurons | 0,95 | | 0,0286 |
| Xenobiotic Metabolism General Signaling Pathway | 0,95 | 0 | 0,0286 |
| Elastic fibre formation | 0,947 | | 0,0455 |
| TBC/RABGAPs | 0,947 | | 0,0455 |
| Carboxyterminal post-translational modifications of tubulin | 0,931 | | 0,0444 |
| Crosstalk between Dendritic Cells and Natural Killer Cells | 0,931 | | 0,033 |
| Cellular Effects of Sildenafil (Viagra) | 0,926 | | 0,028 |
| Fcγ Receptor-mediated Phagocytosis in Macrophages and Monocytes | 0,911 | | 0,0323 |
| TEC Kinase Signaling | 0,908 | -1 | 0,0212 |
| Neurotransmitter clearance | 0,908 | | 0,1 |
| Death Receptor Signaling | 0,901 | | 0,0319 |
| Eukaryotic Translation Termination | 0,901 | | 0,0319 |
| Immunoregulatory interactions between a Lymphoid and a non-Lymphoid cell | 0,897 | 1,341640786 | 0,0248 |
| ERBB Signaling | 0,891 | | 0,0316 |
| 3-phosphoinositide Biosynthesis | 0,878 | 0 | 0,0244 |
| Erythrocytes take up carbon dioxide and release oxygen | 0,87 | | 0,0909 |
| Ephrin A Signaling | 0,87 | | 0,0408 |
| Signaling by NOTCH3 | 0,87 | | 0,0408 |
| Protein folding | 0,862 | | 0,0306 |
| Colorectal Cancer Metastasis Signaling | 0,862 | -1 | 0,0226 |
| Netrin-1 signaling | 0,856 | | 0,04 |
| RNA Polymerase I Transcription | 0,856 | | 0,04 |
| Signaling by EGFR | 0,856 | | 0,04 |
| TNFR1 Signaling | 0,856 | | 0,04 |
| Aspartate and asparagine metabolism | 0,835 | | 0,0833 |
| Assembly of RNA Polymerase I Complex | 0,835 | | 0,0833 |
| Glycogen Degradation III | 0,835 | | 0,0833 |
| UDP-N-acetyl-D-galactosamine Biosynthesis II | 0,835 | | 0,0833 |
| Phase I - Functionalization of compounds | 0,834 | | 0,0297 |
| Response of EIF2AK4 (GCN2) to amino acid deficiency | 0,816 | | 0,0291 |
| Transport of inorganic cations/anions and amino acids/oligopeptides | 0,807 | | 0,0288 |
| Guanosine Nucleotides Degradation III | 0,803 | | 0,0769 |

| | | | |
|---|---|---|---|
| Passive transport by Aquaporins | 0,803 | | 0,0769 |
| Processing of SMDT1 | 0,803 | | 0,0769 |
| Trafficking and processing of endosomal TLR | 0,803 | | 0,0769 |
| Cargo recognition for clathrin-mediated endocytosis | 0,798 | | 0,0286 |
| PD-1, PD-L1 cancer immunotherapy pathway | 0,79 | | 0,0283 |
| CSDE1 Signaling Pathway | 0,79 | | 0,0364 |
| E3 ubiquitin ligases ubiquitinate target proteins | 0,79 | | 0,0364 |
| SPINK1 Pancreatic Cancer Pathway | 0,79 | | 0,0364 |
| Signaling by PTK6 | 0,79 | | 0,0364 |
| Aspirin ADME | 0,773 | | 0,0714 |
| Choline Biosynthesis III | 0,773 | | 0,0714 |
| Glutamate and glutamine metabolism | 0,773 | | 0,0714 |
| Urate Biosynthesis/Inosine 5'-phosphate Degradation | 0,773 | | 0,0714 |
| Neurexins and neuroligins | 0,766 | | 0,0351 |
| CNTF Signaling | 0,755 | | 0,0345 |
| CXCR4 Signaling | 0,747 | | 0,0238 |
| Platelet Adhesion to exposed collagen | 0,746 | | 0,0667 |
| Prolactin receptor signaling | 0,746 | | 0,0667 |
| Metabolism of water-soluble vitamins and cofactors | 0,743 | | 0,0339 |
| IL-12 Signaling and Production in Macrophages | 0,734 | 1,341640786 | 0,0217 |
| Superpathway of Inositol Phosphate Compounds | 0,723 | 0 | 0,0216 |
| Glutaryl-CoA Degradation | 0,721 | | 0,0625 |
| Granzyme B Signaling | 0,721 | | 0,0625 |
| Parkinson's Signaling | 0,721 | | 0,0625 |
| Kinesins | 0,721 | | 0,0328 |
| NCAM signaling for neurite out-growth | 0,721 | | 0,0328 |
| Heparan Sulfate Biosynthesis (Late Stages) | 0,71 | | 0,0323 |
| FOXO-mediated transcription of cell cycle genes | 0,697 | | 0,0588 |
| Isoleucine Degradation I | 0,697 | | 0,0588 |
| Protein methylation | 0,697 | | 0,0588 |
| Th1 Pathway | 0,689 | | 0,0252 |
| Mitochondrial protein import | 0,689 | | 0,0312 |
| Phospholipases | 0,689 | | 0,0312 |
| IL-15 Production | 0,682 | | 0,025 |
| p38 MAPK Signaling | 0,682 | | 0,025 |
| AMPK Signaling | 0,679 | -1,341640786 | 0,0207 |
| Macroautophagy | 0,679 | | 0,0308 |
| RAB geranylgeranylation | 0,679 | | 0,0308 |
| Thrombopoietin Signaling | 0,679 | | 0,0308 |
| Branched-chain amino acid catabolism | 0,675 | | 0,0556 |
| Methylglyoxal Degradation III | 0,675 | | 0,0556 |
| Nicotinate metabolism | 0,675 | | 0,0556 |
| Eukaryotic Translation Initiation | 0,668 | | 0,0246 |
| Renin-Angiotensin Signaling | 0,661 | | 0,0244 |
| CTLA4 Signaling in Cytotoxic T Lymphocytes | 0,657 | -1,133893419 | 0,0187 |
| Docosahexaenoic Acid (DHA) Signaling | 0,655 | -0,447213595 | 0,0203 |
| FOXO-mediated transcription | 0,654 | | 0,0526 |
| Synaptogenesis Signaling Pathway | 0,65 | -2,449489743 | 0,0193 |
| Role of JAK1 and JAK3 in γc Cytokine Signaling | 0,65 | | 0,0294 |
| Heparan Sulfate Biosynthesis | 0,641 | | 0,029 |
| Phospholipase C Signaling | 0,636 | 0,447213595 | 0,017 |

| | | | |
|---|---|---|---|
| Inflammasome pathway | 0,635 | | 0,05 |
| Interleukin-12 family signaling | 0,635 | | 0,05 |
| Valine Degradation I | 0,635 | | 0,05 |
| GNRH Signaling | 0,633 | -2 | 0,0213 |
| ISG15 antiviral mechanism | 0,623 | | 0,0282 |
| Production of Nitric Oxide and Reactive Oxygen Species in Macrophages | 0,622 | | 0,0211 |
| Nucleotide salvage | 0,616 | | 0,0476 |
| Synaptic adhesion-like molecules | 0,616 | | 0,0476 |
| The Visual Cycle | 0,616 | | 0,0476 |
| Dendritic Cell Maturation | 0,614 | 1,889822365 | 0,0181 |
| GM-CSF Signaling | 0,614 | | 0,0278 |
| Growth Hormone Signaling | 0,614 | | 0,0278 |
| Myelination Signaling Pathway | 0,61 | -1,632993162 | 0,0187 |
| fMLP Signaling in Neutrophils | 0,609 | | 0,0229 |
| Extra-nuclear estrogen signaling | 0,606 | | 0,0274 |
| Iron homeostasis signaling pathway | 0,603 | | 0,0227 |
| Role of PKR in Interferon Induction and Antiviral Response | 0,603 | | 0,0227 |
| Cellular hexose transport | 0,599 | | 0,0455 |
| NAD Salvage Pathway II | 0,599 | | 0,0455 |
| ERK5 Signaling | 0,597 | | 0,027 |
| Hypoxia Signaling in the Cardiovascular System | 0,597 | | 0,027 |
| trans-Golgi Network Vesicle Budding | 0,597 | | 0,027 |
| IL-7 Signaling Pathway | 0,589 | | 0,0267 |
| Pulmonary Healing Signaling Pathway | 0,588 | -1 | 0,0203 |
| Protein Kinase A Signaling | 0,586 | -1 | 0,0178 |
| Gα12/13 Signaling | 0,585 | | 0,0222 |
| Pyrimidine Deoxyribonucleotides De Novo Biosynthesis I | 0,582 | | 0,0435 |
| Tryptophan Degradation III (Eukaryotic) | 0,582 | | 0,0435 |
| Plasma lipoprotein assembly, remodeling, and clearance | 0,581 | | 0,0263 |
| GDNF Family Ligand-Receptor Interactions | 0,573 | | 0,026 |
| Macropinocytosis Signaling | 0,573 | | 0,026 |
| Growth hormone receptor signaling | 0,567 | | 0,0417 |
| IL-22 Signaling | 0,567 | | 0,0417 |
| Interleukin-6 family signaling | 0,567 | | 0,0417 |
| Sulfur amino acid metabolism | 0,567 | | 0,0417 |
| Angiopoietin Signaling | 0,565 | | 0,0256 |
| Costimulation by the CD28 family | 0,565 | | 0,0256 |
| ID1 Signaling Pathway | 0,564 | -1 | 0,0198 |
| DHCR24 Signaling Pathway | 0,562 | | 0,0216 |
| Mitotic Prometaphase | 0,559 | -1 | 0,0197 |
| Role of Tissue Factor in Cancer | 0,559 | -1 | 0,0197 |
| Gαi Signaling | 0,556 | | 0,0214 |
| Glucose metabolism | 0,55 | | 0,025 |
| Neurotrophin/TRK Signaling | 0,55 | | 0,025 |
| Insulin Secretion Signaling Pathway | 0,549 | -1,341640786 | 0,0185 |
| BEX2 Signaling Pathway | 0,543 | | 0,0247 |
| IL-3 Signaling | 0,543 | | 0,0247 |
| Role of MAPK Signaling in the Pathogenesis of Influenza | 0,543 | | 0,0247 |
| Pathogen Induced Cytokine Storm Signaling Pathway | 0,541 | 0,816496581 | 0,0176 |
| Fertilization | 0,537 | | 0,0385 |

| | | | |
|---|---|---|---|
| Interleukin-20 family signaling | 0,537 | | 0,0385 |
| Role of JAK1, JAK2 and TYK2 in Interferon Signaling | 0,537 | | 0,0385 |
| FLT3 Signaling in Hematopoietic Progenitor Cells | 0,528 | | 0,0241 |
| Effects of PIP2 hydrolysis | 0,524 | | 0,037 |
| Activation of NMDA receptors and postsynaptic events | 0,521 | | 0,0238 |
| JAK/STAT Signaling | 0,521 | | 0,0238 |
| Transcriptional regulation of white adipocyte differentiation | 0,521 | | 0,0238 |
| HIPPO signaling | 0,514 | | 0,0235 |
| Regulation of the Epithelial Mesenchymal Transition in Development Pathway | 0,514 | | 0,0235 |
| Molecular Mechanisms of Cancer | 0,513 | -2,496150883 | 0,0155 |
| Keratinization | 0,512 | -2 | 0,0187 |
| MTOR signalling | 0,51 | | 0,0357 |
| Apelin Adipocyte Signaling Pathway | 0,507 | | 0,0233 |
| Platelet homeostasis | 0,507 | | 0,0233 |
| VEGF Family Ligand-Receptor Interactions | 0,507 | | 0,0233 |
| LPS-stimulated MAPK Signaling | 0,501 | | 0,023 |
| IL-10 Signaling | 0,499 | | 0,0199 |
| EIF2 Signaling | 0,499 | -2 | 0,0184 |
| Energy dependent regulation of mTOR by LKB1-AMPK | 0,498 | | 0,0345 |
| RAB GEFs exchange GTP for GDP on RABs | 0,488 | | 0,0225 |
| Unfolded protein response | 0,488 | | 0,0225 |
| Mitophagy | 0,486 | | 0,0333 |
| Nucleotide catabolism | 0,486 | | 0,0333 |
| Protein ubiquitination | 0,486 | | 0,0333 |
| Regulation of TP53 Activity through Acetylation | 0,486 | | 0,0333 |
| Signaling by CSF3 (G-CSF) | 0,486 | | 0,0333 |
| Thrombin Signaling | 0,479 | | 0,018 |
| Cristae formation | 0,474 | | 0,0323 |
| Signaling by CSF1 (M-CSF) in myeloid cells | 0,474 | | 0,0323 |
| Ceramide Signaling | 0,463 | | 0,0215 |
| G-protein beta:gamma signalling | 0,463 | | 0,0312 |
| Class B/2 (Secretin family receptors) | 0,457 | | 0,0213 |
| Activation of the pre-replicative complex | 0,452 | | 0,0303 |
| Nucleotide Excision Repair | 0,445 | | 0,0208 |
| Prolactin Signaling | 0,445 | | 0,0208 |
| Salvage Pathways of Pyrimidine Ribonucleotides | 0,445 | | 0,0208 |
| TGF-β Signaling | 0,445 | | 0,0208 |
| IL-4 Signaling | 0,445 | 0 | 0,0162 |
| Cargo concentration in the ER | 0,442 | | 0,0294 |
| Superpathway of Methionine Degradation | 0,442 | | 0,0294 |
| Coagulation System | 0,432 | | 0,0286 |
| GPVI-mediated activation cascade | 0,432 | | 0,0286 |
| Metabolism of steroid hormones | 0,432 | | 0,0286 |
| Gαq Signaling | 0,431 | | 0,0181 |
| Glycerophospholipid biosynthesis | 0,429 | | 0,0202 |
| UVA-Induced MAPK Signaling | 0,429 | | 0,0202 |
| Melanocyte Development and Pigmentation Signaling | 0,423 | | 0,02 |
| IL-9 Signaling | 0,422 | | 0,0278 |
| Interferon Signaling | 0,422 | | 0,0278 |
| RHO GTPases Activate WASPs and WAVEs | 0,422 | | 0,0278 |

| | | | |
|---|---|---|---|
| Striated Muscle Contraction | 0,422 | | 0,0278 |
| Th1 and Th2 Activation Pathway | 0,419 | | 0,0178 |
| Sumoylation Pathway | 0,418 | | 0,0198 |
| Erythropoietin Signaling Pathway | 0,415 | | 0,0176 |
| Apoptosis Signaling | 0,413 | | 0,0196 |
| WNT/β-catenin Signaling | 0,411 | | 0,0175 |
| Pancreatic Secretion Signaling Pathway | 0,408 | 0 | 0,0165 |
| Integration of energy metabolism | 0,407 | | 0,0194 |
| Mitotic Prophase | 0,407 | | 0,0194 |
| Cardiac Hypertrophy Signaling (Enhanced) | 0,405 | -0,447213595 | 0,0151 |
| Pyrimidine Ribonucleotides Interconversion | 0,404 | | 0,0263 |
| Granulocyte Adhesion and Diapedesis | 0,399 | | 0,0172 |
| Sperm Motility | 0,398 | | 0,0163 |
| Formation of Fibrin Clot (Clotting Cascade) | 0,396 | | 0,0256 |
| α-Adrenergic Signaling | 0,392 | | 0,0189 |
| Mechanisms of Viral Exit from Host Cells | 0,379 | | 0,0244 |
| Pyrimidine Ribonucleotides De Novo Biosynthesis | 0,379 | | 0,0244 |
| Transport of vitamins, nucleosides, and related molecules | 0,379 | | 0,0244 |
| Dopamine-DARPP32 Feedback in cAMP Signaling | 0,377 | | 0,0167 |
| Interleukin-4 and Interleukin-13 signaling | 0,373 | | 0,0182 |
| Role of MAPK Signaling in Promoting the Pathogenesis of Influenza | 0,373 | | 0,0182 |
| Formation of WDR5-containing histone-modifying complexes | 0,371 | | 0,0238 |
| Retinol Biosynthesis | 0,371 | | 0,0238 |
| Cardiac Hypertrophy Signaling | 0,367 | | 0,0157 |
| Signaling by Retinoic Acid | 0,364 | | 0,0233 |
| Signaling by SCF-KIT | 0,364 | | 0,0233 |
| Binding and Uptake of Ligands by Scavenger Receptors | 0,364 | | 0,0179 |
| Estrogen Receptor Signaling | 0,363 | -2 | 0,0149 |
| Prostate Cancer Signaling | 0,36 | | 0,0177 |
| MicroRNA Biogenesis Signaling Pathway | 0,359 | | 0,0162 |
| Major pathway of rRNA processing in the nucleolus and cytosol | 0,356 | | 0,0161 |
| Regulation of mRNA stability by proteins that bind AU-rich elements | 0,356 | | 0,0227 |
| SUMOylation of transcription cofactors | 0,356 | | 0,0227 |
| Bladder Cancer Signaling | 0,355 | | 0,0175 |
| Systemic Lupus Erythematosus in T Cell Signaling Pathway | 0,35 | 0,816496581 | 0,0147 |
| GPER1 signaling | 0,349 | | 0,0222 |
| iNOS Signaling | 0,349 | | 0,0222 |
| Cholecystokinin/Gastrin-mediated Signaling | 0,343 | | 0,0171 |
| Interleukin-2 family signaling | 0,342 | | 0,0217 |
| Synaptic Long Term Depression | 0,339 | | 0,0157 |
| ESR-mediated signaling | 0,338 | | 0,0169 |
| MSP-RON Signaling in Macrophages Pathway | 0,338 | | 0,0169 |
| Regulation of lipid metabolism by PPARalpha | 0,334 | | 0,0168 |
| Regulation of the Epithelial Mesenchymal Transition by Growth Factors Pathway | 0,33 | | 0,0155 |
| Apelin Muscle Signaling Pathway | 0,329 | | 0,0208 |
| DNA Damage Bypass | 0,329 | | 0,0208 |
| G alpha (z) signalling events | 0,329 | | 0,0208 |
| XBP1(S) activates chaperone genes | 0,329 | | 0,0208 |

| | | | |
|---|---|---|---|
| Adrenomedullin signaling pathway | 0,327 | | 0,0154 |
| Coronavirus Pathogenesis Pathway | 0,327 | | 0,0154 |
| Regulation of the Epithelial-Mesenchymal Transition Pathway | 0,327 | | 0,0154 |
| NGF Signaling | 0,326 | | 0,0165 |
| PFKFB4 Signaling Pathway | 0,322 | | 0,0204 |
| Transcriptional activity of SMAD2/SMAD3:SMAD4 heterotrimer | 0,322 | | 0,0204 |
| LXR/RXR Activation | 0,319 | | 0,0163 |
| Cell Cycle: G2/M DNA Damage Checkpoint Regulation | 0,316 | | 0,02 |
| FAT10 Cancer Signaling Pathway | 0,316 | | 0,02 |
| NR1H2 and NR1H3-mediated signaling | 0,316 | | 0,02 |
| Opioid Signaling Pathway | 0,313 | -2 | 0,0145 |
| UVC-Induced MAPK Signaling | 0,31 | | 0,0196 |
| Cilium Assembly | 0,309 | | 0,0149 |
| B-WICH complex positively regulates rRNA expression | 0,304 | | 0,0192 |
| Melanoma Signaling | 0,304 | | 0,0192 |
| Primary Immunodeficiency Signaling | 0,304 | | 0,0192 |
| Synaptic Long Term Potentiation | 0,3 | | 0,0156 |
| Signaling by TGF-beta Receptor Complex | 0,298 | | 0,0189 |
| Endocannabinoid Developing Neuron Pathway | 0,296 | | 0,0155 |
| mTOR Signaling | 0,294 | | 0,0146 |
| Amino acids regulate mTORC1 | 0,287 | | 0,0182 |
| Intrinsic Pathway for Apoptosis | 0,287 | | 0,0182 |
| The citric acid (TCA) cycle and respiratory electron transport | 0,287 | | 0,0182 |
| Multiple Sclerosis Signaling Pathway | 0,284 | | 0,0143 |
| Processing of Capped Intron-Containing Pre-mRNA | 0,284 | -2 | 0,0139 |
| Complement cascade | 0,283 | | 0,015 |
| Cell Cycle Control of Chromosomal Replication | 0,282 | | 0,0179 |
| DNA Damage/Telomere Stress Induced Senescence | 0,282 | | 0,0179 |
| CGAS-STING Signaling Pathway | 0,279 | | 0,0149 |
| Th2 Pathway | 0,276 | | 0,0148 |
| Insulin Receptor Signaling | 0,273 | | 0,0147 |
| Cancer Drug Resistance by Drug Efflux | 0,272 | | 0,0172 |
| EGF Signaling | 0,272 | | 0,0172 |
| Role of JAK2 in Hormone-like Cytokine Signaling | 0,272 | | 0,0172 |
| Signaling by ERBB4 | 0,272 | | 0,0172 |
| Adipogenesis pathway | 0,27 | | 0,0146 |
| White Adipose Tissue Browning Pathway | 0,27 | | 0,0146 |
| DNA Double Strand Break Response | 0,267 | | 0,0169 |
| Polyamine Regulation in Colon Cancer | 0,267 | | 0,0169 |
| GABA receptor activation | 0,262 | | 0,0167 |
| PCP (Planar Cell Polarity) Pathway | 0,262 | | 0,0167 |
| Visual phototransduction | 0,262 | | 0,0167 |
| Role of Chondrocytes in Rheumatoid Arthritis Signaling Pathway | 0,26 | | 0,0143 |
| Apelin Endothelial Signaling Pathway | 0,257 | | 0,0142 |
| MSP-RON Signaling in Cancer Cells Pathway | 0,257 | | 0,0142 |
| Cytoprotection by HMOX1 | 0,253 | | 0,0161 |
| T Cell Receptor Signaling | 0,252 | 0,447213595 | 0,0131 |
| Cytotoxic T Lymphocyte-mediated Apoptosis of Target Cells | 0,251 | | 0,0135 |
| Role of Osteoblasts, Osteoclasts and Chondrocytes in Rheumatoid Arthritis | 0,249 | | 0,0134 |

| | | | |
|---|---|---|---|
| Activation of IRF by Cytosolic Pattern Recognition Receptors | 0,248 | | 0,0159 |
| SPINK1 General Cancer Pathway | 0,248 | | 0,0159 |
| IL-2 Signaling | 0,244 | | 0,0156 |
| NAD Signaling Pathway | 0,243 | | 0,0137 |
| Pyridoxal 5'-phosphate Salvage Pathway | 0,24 | | 0,0154 |
| RNA Polymerase II Transcription | 0,237 | | 0,0135 |
| ERB2-ERBB3 Signaling | 0,235 | | 0,0152 |
| Eicosanoid Signaling | 0,231 | | 0,0149 |
| Phase II - Conjugation of compounds | 0,231 | | 0,0149 |
| ERBB4 Signaling | 0,227 | | 0,0147 |
| COPII-mediated vesicle transport | 0,212 | | 0,0139 |
| Senescence-Associated Secretory Phenotype (SASP) | 0,209 | | 0,0137 |
| GPCR-Mediated Integration of Enteroendocrine Signaling Exemplified by an L Cell | 0,205 | | 0,0135 |
| Antiproliferative Role of Somatostatin Receptor 2 | 0,198 | | 0,0132 |
| Pre-NOTCH Expression and Processing | 0,198 | | 0,0132 |
| Role of MAPK Signaling in Inhibiting the Pathogenesis of Influenza | 0,198 | | 0,0132 |
| SUMOylation of DNA damage response and repair proteins | 0,198 | | 0,0132 |
| ATM Signaling | 0 | | 0,0101 |
| Activin Inhibin Signaling Pathway | 0 | | 0,00948 |
| Acute Myeloid Leukemia Signaling | 0 | | 0,0109 |
| Adrenergic Receptor Signaling Pathway (Enhanced) | 0 | | 0,00518 |
| Airway Pathology in Chronic Obstructive Pulmonary Disease | 0 | | 0,00885 |
| Allograft Rejection Signaling | 0 | | 0,012 |
| Altered T Cell and B Cell Signaling in Rheumatoid Arthritis | 0 | | 0,00424 |
| Amyotrophic Lateral Sclerosis Signaling | 0 | | 0,00862 |
| Androgen Signaling | 0 | | 0,00599 |
| Apelin Cardiomyocyte Signaling Pathway | 0 | | 0,01 |
| Autoimmune Thyroid Disease Signaling | 0 | | 0,008 |
| B Cell Development | 0 | | 0,00905 |
| B Cell Receptor Signaling | 0 | 0 | 0,0108 |
| BMP signaling pathway | 0 | | 0,0112 |
| Beta-catenin independent WNT signaling | 0 | | 0,0098 |
| Breast Cancer Regulation by Stathmin1 | 0 | 1,341640786 | 0,00853 |
| CCR5 Signaling in Macrophages | 0 | | 0,00338 |
| CD28 Signaling in T Helper Cells | 0 | | 0,00958 |
| CREB Signaling in Neurons | 0 | | 0,00503 |
| Calcium Signaling | 0 | | 0,00948 |
| Calcium-induced T Lymphocyte Apoptosis | 0 | | 0,00791 |
| Cardiac conduction | 0 | | 0,00769 |

| | | | |
|---|---|---|---|
| Cardiac β-adrenergic Signaling | 0 | | 0,0113 |
| Chemokine Signaling | 0 | | 0,013 |
| Chronic Myeloid Leukemia Signaling | 0 | | 0,0036 8 |
| Circadian Rhythm Signaling | 0 | | 0,0076 |
| Class A/1 (Rhodopsin-like receptors) | 0 | | 0,0031 5 |
| Communication between Innate and Adaptive Immune Cells | 0 | | 0,0064 7 |
| Corticotropin Releasing Hormone Signaling | 0 | | 0,0068 |
| Dopamine Receptor Signaling | 0 | | 0,0127 |
| Electron transport, ATP synthesis, and heat production by uncoupling proteins | 0 | | 0,0078 1 |
| Endocannabinoid Neuronal Synapse Pathway | 0 | | 0,0067 6 |
| Estrogen-Dependent Breast Cancer Signaling | 0 | | 0,0123 |
| FAK Signaling | 0 | -0,632455532 | 0,0121 |
| FXR/RXR Activation | 0 | | 0,0113 |
| Factors Promoting Cardiogenesis in Vertebrates | 0 | | 0,0067 1 |
| Fc Epsilon RI Signaling | 0 | | 0,0083 3 |
| Fcgamma receptor (FCGR) dependent phagocytosis | 0 | | 0,0063 7 |
| FcγRIIB Signaling in B Lymphocytes | 0 | | 0,0036 5 |
| G Protein Signaling Mediated by Tubby | 0 | | 0,0037 6 |
| G alpha (i) signalling events | 0 | | 0,0038 6 |
| G alpha (s) signalling events | 0 | | 0,0069 |
| G-Protein Coupled Receptor Signaling | 0 | -0,377964473 | 0,0101 |
| GABA Receptor Signaling | 0 | | 0,0076 9 |
| GABAergic Receptor Signaling Pathway (Enhanced) | 0 | | 0,0072 5 |
| GPCR-Mediated Nutrient Sensing in Enteroendocrine Cells | 0 | | 0,0085 5 |
| Generic Transcription Pathway | 0 | | 0,0023 4 |
| Glioblastoma Multiforme Signaling | 0 | | 0,0118 |
| Glioma Signaling | 0 | | 0,0080 6 |
| Glutaminergic Receptor Signaling Pathway (Enhanced) | 0 | -2 | 0,0125 |
| Graft-versus-Host Disease Signaling | 0 | | 0,0082 6 |
| Gαs Signaling | 0 | | 0,0080 6 |
| HMGB1 Signaling | 0 | | 0,0124 |
| HOTAIR Regulatory Pathway | 0 | | 0,0124 |
| Hereditary Breast Cancer Signaling | 0 | | 0,0070 9 |
| Human Embryonic Stem Cell Pluripotency | 0 | | 0,0050 3 |
| ICOS-ICOSL Signaling in T Helper Cells | 0 | | 0,0066 |
| IL-1 Signaling | 0 | | 0,0104 |

| | | | |
|---|---|---|---|
| IL-13 Signaling Pathway | 0 | | 0,0085 5 |
| IL-15 Signaling | 0 | | 0,0037 5 |
| IL-17 Signaling | 0 | | 0,0055 2 |
| Ion channel transport | 0 | | 0,0109 |
| Kinetochore Metaphase Signaling Pathway | 0 | | 0,0093 5 |
| Lipid Antigen Presentation by CD1 | 0 | | 0,0047 2 |
| Macrophage Alternative Activation Signaling Pathway | 0 | | 0,011 |
| Macrophage Classical Activation Signaling Pathway | 0 | | 0,0055 6 |
| Maturity Onset Diabetes of Young (MODY) Signaling | 0 | | 0,013 |
| Mitochondrial Dysfunction | 0 | 2 | 0,012 |
| Mouse Embryonic Stem Cell Pluripotency | 0 | | 0,0095 2 |
| NAFLD Signaling Pathway | 0 | | 0,0046 1 |
| NER (Nucleotide Excision Repair, Enhanced Pathway) | 0 | | 0,0114 |
| NF-κB Signaling | 0 | | 0,0054 2 |
| NOD1/2 Signaling Pathway | 0 | | 0,0114 |
| NUR77 Signaling in T Lymphocytes | 0 | | 0,0109 |
| Necroptosis Signaling Pathway | 0 | | 0,0131 |
| Netrin Signaling | 0 | | 0,0116 |
| Neuroinflammation Signaling Pathway | 0 | 2 | 0,0129 |
| Neurovascular Coupling Signaling Pathway | 0 | | 0,0091 3 |
| Nitric Oxide Signaling in the Cardiovascular System | 0 | | 0,0087 |
| Non-Small Cell Lung Cancer Signaling | 0 | | 0,0105 |
| OX40 Signaling Pathway | 0 | | 0,0123 |
| Orexin Signaling Pathway | 0 | | 0,0042 7 |
| Ovarian Cancer Signaling | 0 | | 0,0063 3 |
| Oxidative Phosphorylation | 0 | | 0,0093 5 |
| Oxytocin in Brain Signaling Pathway | 0 | | 0,0102 |
| P2Y Purigenic Receptor Signaling Pathway | 0 | | 0,0074 6 |
| PEDF Signaling | 0 | | 0,0116 |
| PI Metabolism | 0 | | 0,0122 |
| PI3K Signaling in B Lymphocytes | 0 | | 0,0061 5 |
| PKCθ Signaling in T Lymphocytes | 0 | | 0,0084 5 |
| PPAR Signaling | 0 | | 0,0096 2 |
| PPARα/RXRα Activation | 0 | | 0,0109 |
| RANK Signaling in Osteoclasts | 0 | | 0,0111 |
| RAR Activation | 0 | -2 | 0,0096 6 |
| Regulation of IL-2 Expression in Activated and Anergic T Lymphocytes | 0 | | 0,0038 2 |

| | | | |
|---|---|---|---|
| Relaxin Signaling | 0 | | 0,0065 4 |
| Role of BRCA1 in DNA Damage Response | 0 | | 0,0125 |
| Role of Hypercytokinemia/hyperchemokinemia in the Pathogenesis of Influenza | 0 | | 0,0125 |
| Role of JAK family kinases in IL-6-type Cytokine Signaling | 0 | | 0,013 |
| Role of Macrophages, Fibroblasts and Endothelial Cells in Rheumatoid Arthritis | 0 | | 0,0062 5 |
| Role of NANOG in Mammalian Embryonic Stem Cell Pluripotency | 0 | | 0,0080 6 |
| Role of NFAT in Cardiac Hypertrophy | 0 | | 0,0090 1 |
| Role of NFAT in Regulation of the Immune Response | 0 | 0 | 0,0070 1 |
| S100 Family Signaling Pathway | 0 | 0 | 0,0107 |
| SAPK/JNK Signaling | 0 | | 0,0066 2 |
| STAT3 Pathway | 0 | | 0,0074 1 |
| Senescence Pathway | 0 | | 0,0033 8 |
| Signaling by NOTCH1 | 0 | | 0,013 |
| Signaling by NTRK1 (TRKA) | 0 | | 0,0123 |
| Sphingolipid metabolism | 0 | | 0,0115 |
| Sphingosine-1-phosphate Signaling | 0 | | 0,0084 |
| Systemic Lupus Erythematosus Signaling | 0 | | 0,0067 6 |
| Systemic Lupus Erythematosus in B Cell Signaling Pathway | 0 | | 0,0044 2 |
| T Cell Exhaustion Signaling Pathway | 0 | | 0,011 |
| T Helper Cell Differentiation | 0 | | 0,0112 |
| TR/RXR Activation | 0 | | 0,0076 9 |
| Telomerase Signaling | 0 | | 0,0093 5 |
| Thyroid Cancer Signaling | 0 | | 0,013 |
| Transcriptional Regulatory Network in Embryonic Stem Cells | 0 | | 0,0061 |
| Transport of bile salts and organic acids, metal ions and amine compounds | 0 | | 0,012 |
| Type I Diabetes Mellitus Signaling | 0 | | 0,0098 7 |
| VEGF Signaling | 0 | | 0,0101 |
| WNK Renal Signaling Pathway | 0 | | 0,01 |
| cAMP-mediated signaling | 0 | | 0,0043 3 |
| p70S6K Signaling | 0 | | 0,0062 5 |

**Online Resource 14 Significantly enriched GO pathways of differentially expressed proteins.**

| **#term ID** | **term description** | **genes mapped** | **enrichment score** | **direction** | **false discovery rate** |
|---|---|---|---|---|---|
| GO:0005509 | Calcium ion binding | 88 | 0.076586 | bottom | 4.61e-07 |
| GO:0140640 | Catalytic activity, acting on a nucleic acid | 69 | 615.908 | top | 4.61e-07 |
| GO:0016462 | Pyrophosphatase activity | 124 | 552.731 | top | 7.46e-06 |

| | | | | | |
|---|---|---|---|---|---|
| GO:0017111 | Nucleoside-triphosphatase activity | 118 | 551.794 | top | 1.08e-05 |
| GO:0140098 | Catalytic activity, acting on RNA | 50 | 606.135 | top | 2.46e-05 |
| GO:0004812 | aminoacyl-tRNA ligase activity | 21 | 671.755 | top | 0.00051 |
| GO:0016874 | Ligase activity | 49 | 57.479 | top | 0.00051 |
| GO:0140101 | Catalytic activity, acting on a tRNA | 23 | 635.538 | top | 0.00058 |
| GO:0005524 | ATP binding | 193 | 344.703 | top | 0.0011 |
| GO:0016884 | Carbon-nitrogen ligase activity, with glutamine as amido-N-donor | 4 | 949.327 | top | 0.0036 |
| GO:0016887 | ATP hydrolysis activity | 67 | 563.654 | top | 0.0037 |
| GO:0004386 | Helicase activity | 27 | 650.903 | top | 0.0092 |

**Online Resource 15 Percentage of responding cells during $Ca^{2+}$ imaging in the cytoplasm of control and TSC astrocytes. a.** The percentage of responding control and TSC astrocytes upon DHPG stimulation. **b.** The percentage of responding control and TSC astrocytes upon glutamate stimulation. **c.** The percentage of responding control and TSC astrocytes upon ATP stimulation. Data are expressed as mean ± SEM; p-value: * p-value ≤ 0.05; ** p-value ≤ 0.01; *** p-value ≤ 0.001; **** p-value ≤ 0.0001. Mann–Whitney U-test.

**Online Resource 16 Electron microscopy analysis of mitochondria morphology in healthy-appearing astrocytes and neurons in TLE control tissue and astrocytes and dysmorphic neurons in TSC tissue. a-c.** Multiple parameters were considered in the analysis to describe mitochondria morphology. TSC astrocytes showed significant reduction in circularity, feret's diameter and integrated density compared to control astrocytes. Data are expressed as mean ± SEM; Non-parametric Kruskal-Wallis H test was performed with correction for multiple comparisons. Significance is represented by p-value: * p-value ≤ 0.05; ** p-value ≤ 0.01; *** p-value ≤ 0.001; **** p-value ≤ 0.0001.
