# Supplementary figures and images for: Astroglial calcium signaling and homeostasis in tuberous sclerosis complex
Source: Acta Neuropathol. 2024 Feb 28;147(1):48. doi: 10.1007/s00401-024-02711-3 (PMC10901927; doi:10.1007/s00401-024-02711-3)

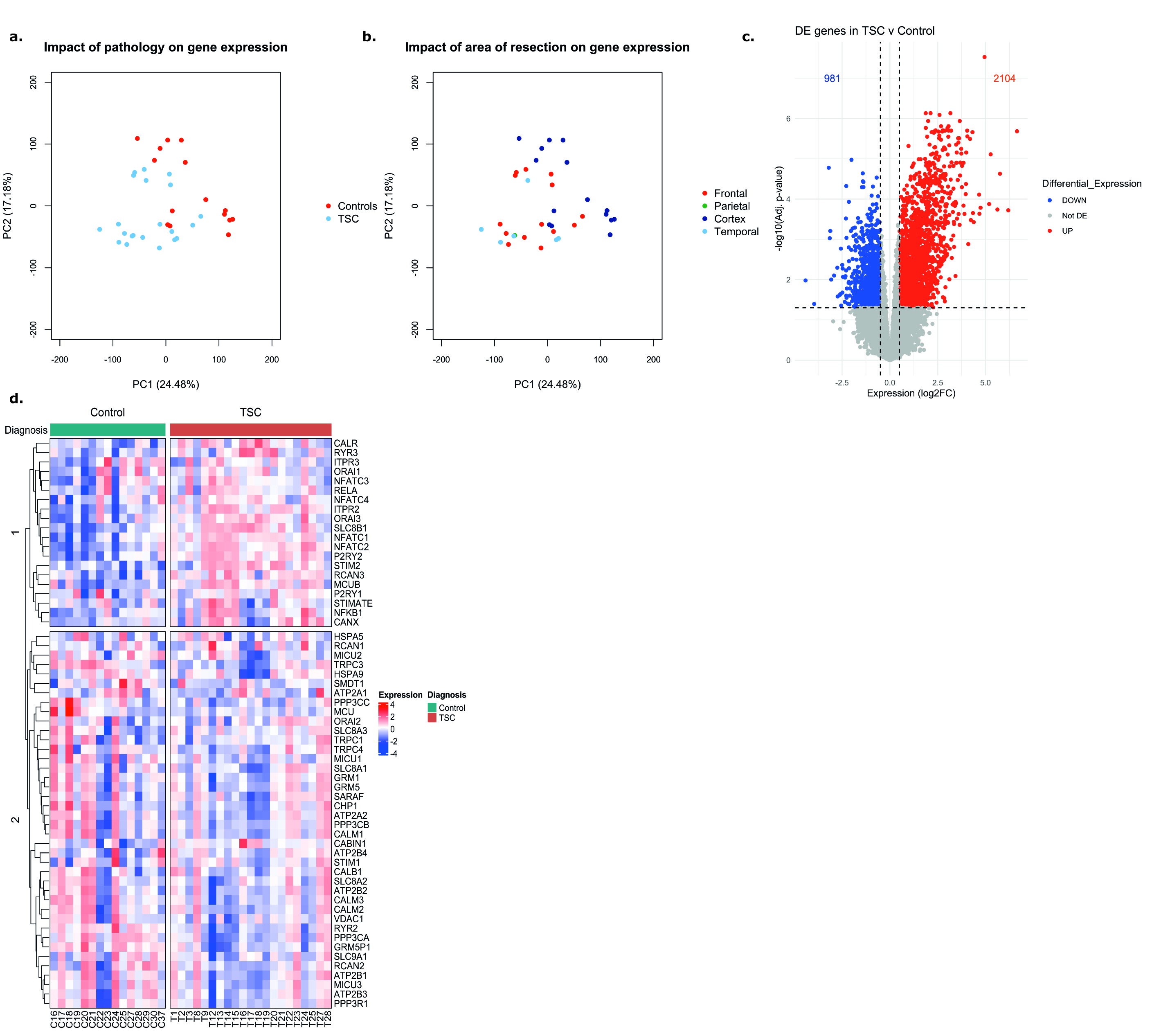

Supplement: Supplementary file 2 — Supplementary file2 (TIF 30433 KB) [file 401_2024_2711_MOESM2_ESM.tif]

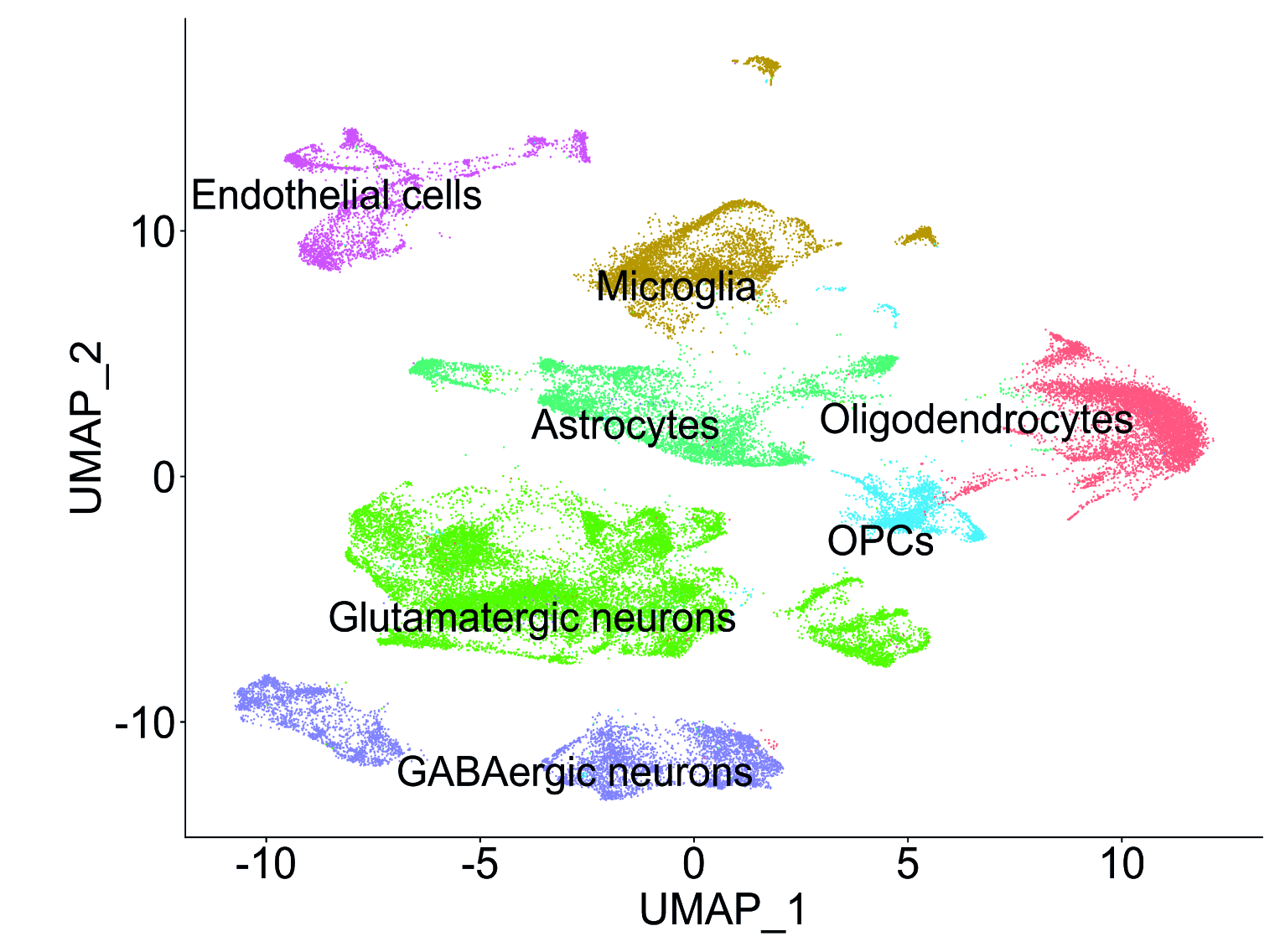

Supplement: Supplementary file 3 — Supplementary file3 (TIF 8179 KB) [file 401_2024_2711_MOESM3_ESM.tif]

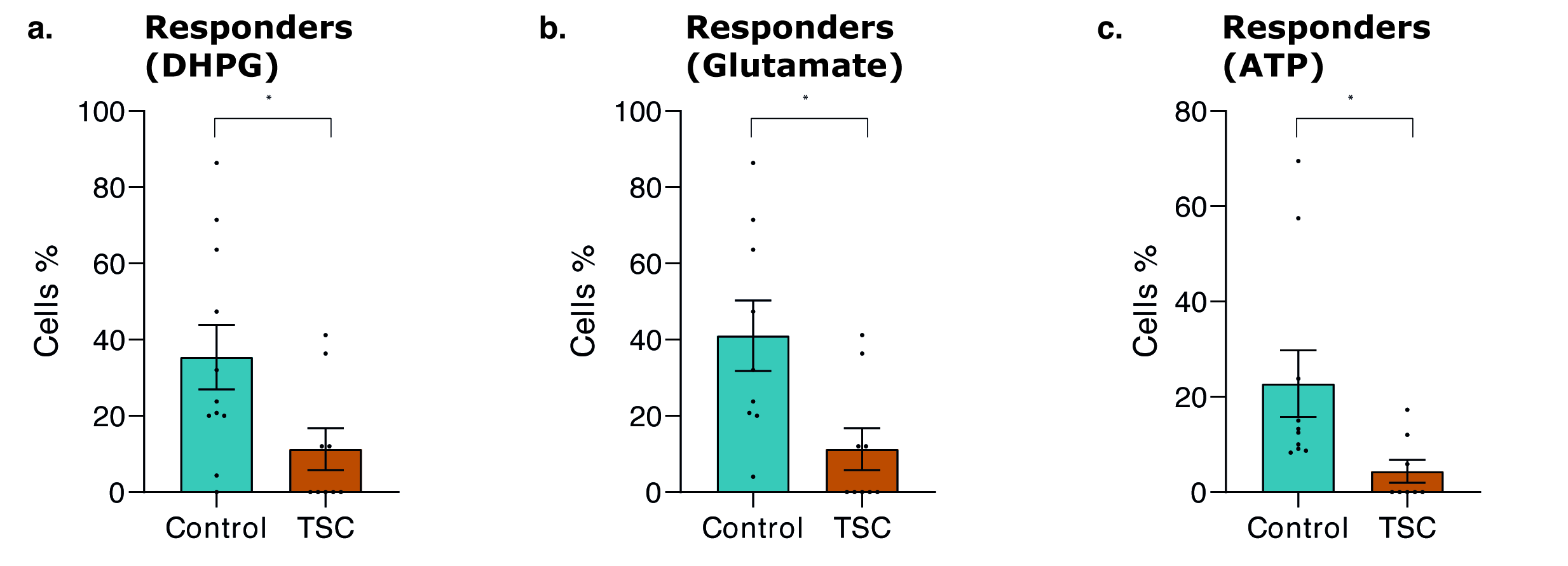

Supplement: Supplementary file 4 — Supplementary file4 (TIF 9692 KB) [file 401_2024_2711_MOESM4_ESM.tif]

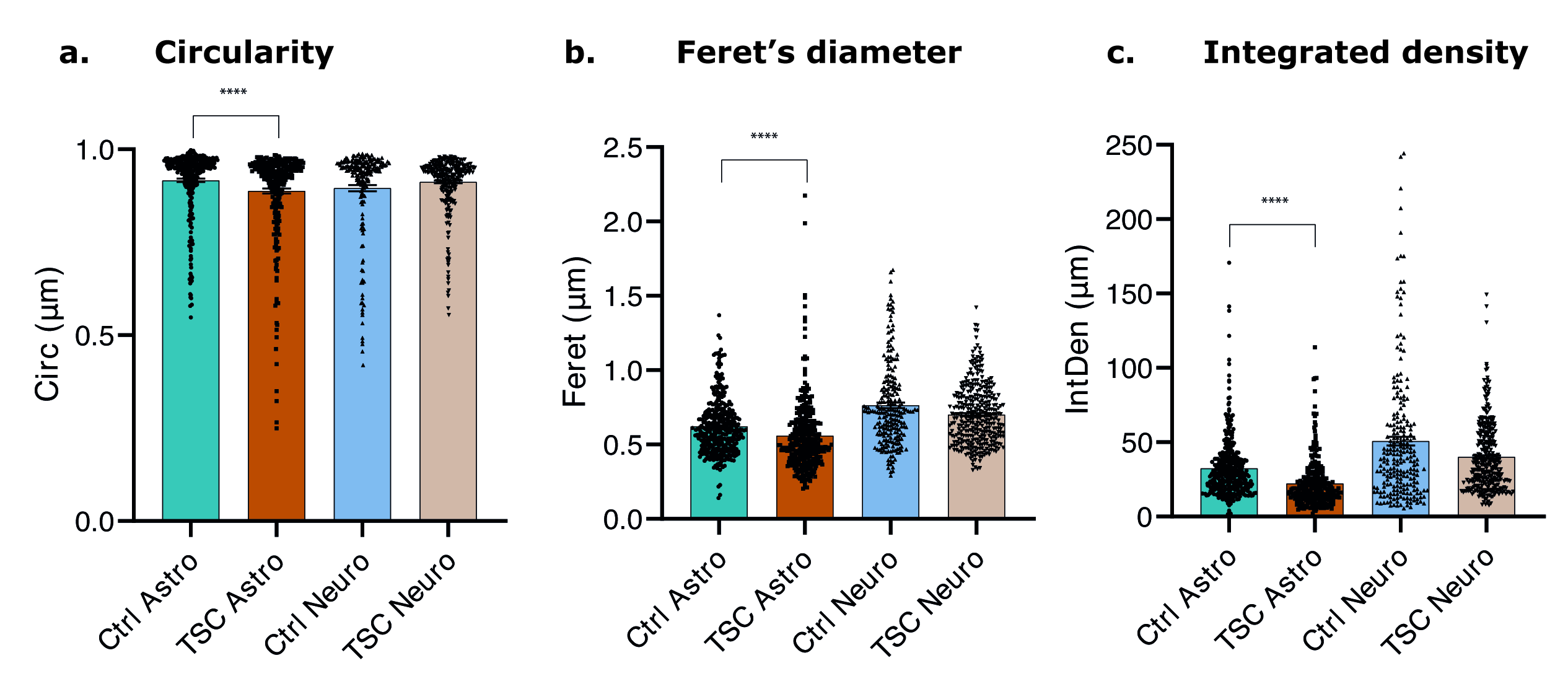

Supplement: Supplementary file 5 — Supplementary file5 (TIF 12495 KB) [file 401_2024_2711_MOESM5_ESM.tif]
